# Supplementary material for: Interventions for treating obstetric fistula: An evidence gap map
Source: PLOS Glob Public Health. 2023 Jan 26;3(1):e0001481. doi: 10.1371/journal.pgph.0001481 (PMC10021774; doi:10.1371/journal.pgph.0001481)
Supplement: S1 Text — (DOCX) [file pgph.0001481.s001.docx]

**S1 Text: Differences between protocol and review**

**Reporting**

- The Consensus-Based Checklist for Reporting of Survey Studies (CROSS) was adopted in place of the Checklist for Reporting Results of Internet E-Surveys (CHERRIES). This is because the CROSS checklist is more up-to-date and has been developed using a validated process.

**Survey methods**

- The survey was distributed via Newcastle University’s Health Economics and Evidence Synthesis Twitter account, Cochrane’s TaskExchange platform and through snowballing via clinical advisors. These were additions to the survey distribution methods originally planned in the protocol, intended to increase the overall reach of the survey.
- Survey respondents were asked whether they believed that any important interventions or outcomes were missing from the list within the survey, but these were not incorporated into the evidence map as originally planned. Instead, they were briefly discussed within the Results. This was a pragmatic decision based on time constraints.

**Evidence map methods**

- Clarification was added to the eligibility criteria regarding what was considered a single-arm study, for transparency.
- In the protocol, cross-sectional studies were named as “cross-sectional surveys”; this wording was altered for clarity within the full review.
- Further clarification surrounding causes of fistula that were excluded and when caesarean section was considered an applicable cause of fistula were added into the eligibility criteria.
- Studies were only included if 80% or more of the participants had fistula due to obstetric causes. This was to ensure that the studies included are roughly generalisable and applicable to the obstetric fistula population, as fewer women with this specific diagnosis would reduce the overall applicability of results.
- A wider range of fistulae were considered eligible for the review than were originally in the protocol. This was to ensure that all types of obstetric fistulae were included within the evidence map and enhance the review’s overall applicability to the population.
- It was specified that if surgery in a study was undertaken either vaginally or abdominally but with no further description, it would not be eligible for the review. This decision was taken after discussion with clinical advisors, who confirmed that the vaginal or abdominal approaches could be applied to numerous surgery types; it would therefore not be possible to code these studies accurately within the evidence map.
- The interventions in the eligibility criteria were narrowed to match the coding tool; this was to ensure that any studies considered eligible for the evidence map would be able to be coded to specific interventions.
- Detail surrounding what types of publication were eligible for the evidence map was added to the eligibility criteria for transparency and clarity.
- The databases searched were altered from the original protocol in response to suggestions by an experienced Information Specialist during the PRESS assessment of the search strategies.
- 20% of full-texts were screened by a second reviewer instead of the 10% originally stated in the protocol; this was to help ensure further accuracy of the screening process.
- Forward and backward citation chaining was undertaken for all included primary studies and systematic reviews instead of only systematic reviews, as stated in the protocol. Due to the limited number of studies included in the evidence map, this was felt to be a reasonable addition in order to minimise the chance of potentially-eligible records being missed.
- Originally, it was planned that only systematic reviews would be critically appraised using the AMSTAR-2 tool. However, due to the limited number of studies included within the evidence map it was decided to critically appraise all included primary studies as well.
- 20% of risk of bias assessments and coding were checked by a second reviewer instead of the 10% pre-stated in the protocol. This was due to the limited number of studies included in the evidence map, to ensure that assessments and coding were accurate.
- The coding for ‘Type of fistula’ was altered in order to capture the range of fistulae that may have been included due to the change in eligibility criteria.
- A filter in the coding tool was added to demonstrate whether studies included at least 80% of women with obstetric fistula, 95% of women with obstetric fistula or whether this was unclear. This was to ensure that a sensitivity analysis was possible to explore the applicability of the overall body of evidence to the core population.
- A filter in the coding tool was added to ascertain whether studies reporting on multiple interventions reported these separately within their results. This was to be able to conduct a sensitivity analysis on specific studies that did not report interventions separately, as these may not present usable data.
- The comparison code was altered in order to clarify the two types of comparison considered eligible for the evidence map.
- The ‘Risk of bias’ code was expanded to reflect how risk of bias assessments were conducted on all primary and secondary studies included in the evidence map.
- A code for additional reports of included studies was included to transparently highlight where allied papers associated with included studies were available.
- The Data Synthesis section was amended and expanded to clarify that the unit of analysis was the study-level and how multiple reports of a single study would be handled within the evidence map.
- Sensitivity analyses were added to the evidence map methods in order to explore the overall applicability of the included body of evidence to the core population and whether the data would be usable in the context of a systematic review.
