# Supplementary material for: Interventions for treating obstetric fistula: An evidence gap map
Source: PLOS Glob Public Health. 2023 Jan 26;3(1):e0001481. doi: 10.1371/journal.pgph.0001481 (PMC10021774; doi:10.1371/journal.pgph.0001481)
Supplement: S2 Text — (DOCX) [file pgph.0001481.s002.docx]

**S2 Text: Finalised survey design**

Standard: Introduction (1 Question)

Standard: Age (1 Question)

Branch: New Branch

If

If Are you over 18 years old? No Is Selected

EndSurvey:

Branch: New Branch

If

If Are you over 18 years old? Yes Is Selected

Block: Demographics (3 Questions)

Standard: Intervention types (1 Question)

Standard: Outcomes (2 Questions)

EndSurvey:

| Page Break |  |
| --- | --- |

Start of Block: Introduction

Q1
**Welcome!**
 
Thank you for your interest in participating in this survey, which will be used to guide the production of an evidence gap map on the topic of obstetric fistula. 
 
Evidence gap maps are used to identify areas in research that require new evidence, identify topics for new systematic reviews or help guide the production of other documentation, such as guidelines. By creating this evidence gap map, we are aiming to understand how much research looking at interventions for treating or managing obstetric fistula has already been carried out and where new research could be needed. 
 
By participating, you will be helping to identify which outcomes are most important to patients, the public, clinicians and researchers with regards to obstetric fistula research. This will help to guide how the evidence gap map is constructed and inform potential recommendations for research. 

Information provided will be shared with members of the project team and findings will be used for this project (the production of an evidence gap map) and any related follow-up projects. All information collected will be kept confidential and will be stored securely by Newcastle University. There will be no connection to you specifically in the results or in future publication of the results.

Your participation in this survey is voluntary and you can withdraw from completing the survey at any point prior to submission without any penalty. 

By continuing past this point, you are giving informed consent to participate in this survey. You are verifying that you have read the explanation of the study, and that you agree to participate. You are also verifying that you understand your participation in this study is strictly voluntary.

This anonymous survey will take no more than 5 to 10 minutes to complete in total. **This survey is open until 14 March 2022.**
 
If you have any questions or issues regarding this survey, please contact Eugenie Johnson: eugenie.johnson@newcastle.ac.uk
 
**You must be over 18 to participate in this survey.**

**Data protection statement:** The information contained within this survey is confidential. Information provided on this form will be shared with members of the project team but will not be distributed to other third parties. No identifying information is being collected in this survey.

End of Block: Introduction

Start of Block: Age

Q2 Are you over 18 years old?

- Yes (1)
- No (2)

End of Block: Age

Start of Block: Demographics

Q3 Which of the following best describes you?

- Clinician (1)
- Patient or member of the public (2)
- Researcher (3)
- Clinical academic (4)
- Other (please indicate) (5) ________________________________________________

Q4 Where in the world are you based?

- Europe (1)
- North America (2)
- Latin America (3)
- Africa (4)
- Asia (5)
- Oceania (6)

Q5 Please indicate which country you are based in.

________________________________________________________________

End of Block: Demographics

Start of Block: Intervention types

Q6 Which of the following do you believe is the most important treatment option for research into obstetric fistula to examine?

- Lifestyle interventions (e.g. skin protection, pads and urethral plugs) (1)
- Catheter insertion (2)
- Physical therapy (e.g. therapeutic exercise, bladder training, electrical stimulation) (3)
- Psychological therapy (e.g. cognitive behavioural therapy) (4)
- Surgical interventions (5)

End of Block: Intervention types

Start of Block: Outcomes

| 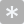 |
| --- |

Q7 Which of the following do you believe are the most important outcomes for research into interventions for managing or treating obstetric fistula? Please tick up to 5.

- Cure or improvement of obstetric fistula (closing or reducing the size of the obstetric fistula) (1)
- Improvement in pain (2)
- Improvement in urinary incontinence (3)
- Improvement in faecal incontinence (4)
- Improvement in difficulties urinating (e.g. slow stream, hesitancy) (5)
- Improvement in difficulties defecating (e.g. constipation, straining on the toilet) (6)
- Improvement in sexual function (e.g. less pain during sex) (7)
- Improvement in haematuria (blood in urine) (8)
- Improvement in quality of life (9)
- Improvement in mental health (e.g. anxiety or depression) (10)
- Improvement in associated pelvic organ prolapse symptoms (11)
- Improvement in urinary retention (12)
- Improvement in activities of daily living (everyday tasks) (13)
- Adverse events of interventions (14)
- Satisfaction with the intervention (15)
- Woman's need for further treatment (16)
- Adherence to the intervention (17)

Q8 Are there any other outcomes not stated above that you believe are important when evaluating the effectiveness of treatment options for fistula?

________________________________________________________________

End of Block: Outcomes
