# Supplementary material for: Interventions for treating obstetric fistula: An evidence gap map
Source: PLOS Glob Public Health. 2023 Jan 26;3(1):e0001481. doi: 10.1371/journal.pgph.0001481 (PMC10021774; doi:10.1371/journal.pgph.0001481)
Supplement: S1 Table — (DOCX) [file pgph.0001481.s003.docx]

**S1 Table: Campbell Collaboration reporting standards for evidence gap maps**

| Item name | Item no. | Standard | Page number |
| --- | --- | --- | --- |
| Format of title | ER1 | Follow the standard Campbell EGM title template. | 1 |
| Authors | ER2 | List names and affiliations of all authors | 1 |
| Writing the abstract | ER3 | Prepare a structured abstract to provide a succinct overview of the EGM. In the interests of brevity it is highly desirable for authors to provide an abstract of less than 700 words, and it should be no more than 1000 words in length. | 1-2 |
| Abstract, Background | ER4 | Summarize the rationale and scope of the EGM. | 1 |
| Abstract, Objectives | ER5 | State the main objective(s), preferably in a single concise sentence. | 1 |
| Abstract, Search methods | ER6 | Provide the date of the last search from which records were evaluated and any studies identified were incorporated into the EGM, and an indication of the databases and other sources searched. | 1 |
| Abstract, Selection criteria | ER7 | Summarize eligibility criteria of the evidence and gap map, including information on types of evidence, interventions, population, outcomes, and setting, as appropriate. | 1 |
| Abstract, Data collection and analysis | ER8 | Summarize any noteworthy methods for identifying studies, collecting data, and evaluating risk of bias/study quality. | 1 |
| Abstract, Main results: number of studies | ER9 | Report the number of included studies, reporting systematic reviews and primary studies separately. | 2 |
| Abstract, Main results: study characteristics | ER10 | Provide a brief description of the coverage of evidence by key dimensions to provide an overview of the body of evidence (e.g. region, and intervention type). Identify clusters of evidence and evidence gaps. | 2 |
| Abstract, Main results: risk of bias, study quality or confidence in included studies | ER11 | Provide a comment on the risk of bias/quality assessments/confidence of the evidence. | 2 |
| Abstract, Implications for research | ER12 | State key conclusions drawn. | 2 |
| Completeness of main report text | ER13 | Ensure that all findings reported in the abstract also appear in the main text of the EGM report. | 2 |
| Consistency of summary versions of the EGM | ER14 | Ensure that reporting of objectives, important outcomes, caveats and conclusions is consistent between the abstract and the main report. | 2 |
| Background | ER15 | Provide a concise description of the scope of the EGM, and why it is important to do the EGM. | 3 |
| Background references | ER16 | Support all key supporting statements with references. | 3 |
| Background text | ER17 | Do not use plagiarized text. | 3 |
| Objectives | ER18 | State the objectives, where appropriate in a single concise sentence. | 5 |
| Types of evidence | ER19 | State the types of evidence being shown in the EGM explicitly in the objectives | 7, S3 |
| Reference protocol | ER20 | Cite the protocol for the EGM. | 2, 5 |
| Eligibility criteria for types of study: types of evidence | ER21 | State eligible types of evidence, and provide a justification for the choice. | 7, S3 |
| Eligibility criteria for types of study: study report status | ER22 | Campbell EGM should include all relevant evidence regardless of publication status and language of publication. Any exceptions should be explicitly stated and justified. | 7, 8 |
| Eligibility criteria for types of participants | ER23 | State eligibility criteria for participants, including any criteria around location, setting, status, or definition of condition and demographic factors, and how studies including subsets of relevant participants are handled. | 7, 8 |
| Eligibility criteria for types of interventions | ER24 | State eligibility criteria for interventions and comparators, including any criteria around delivery, dose, duration, intensity, co-interventions, and characteristics of complex interventions. | 8 |
| Role of outcomes | ER25 | State whether outcomes are used as eligibility. If so, define. | 8 |
| Outcomes of interest | ER26 | State outcomes of interest to the EGM, and define acceptable ways of measuring them. | 8, S3 |
| Search sources | ER27 | List all sources searched, including: databases, trials registers, web sites, and grey literature. Database names should include platform/provider name and dates of coverage; web sites should include full name and URL. State whether reference lists were searched and whether individuals or organizations were contacted. Indicate whether stakeholders were consulted. | 7, S2 |
| Latest sources | ER28 | Provide the date of the last search and the issue/version number (where relevant) for each database whose results were evaluated and incorporated into the EGM. If a search was re-run prior to publication, the results of which were not incorporated, explain how the results were dealt with and provide the date. | 7 |
| Search restrictions | ER29 | Specify and justify any restrictions placed on the time period covered by the search. | 7 |
| Searches for different types of evidence | ER30 | If the EGM has specific eligibility criteria to include additional studies such as studies of adverse effects, economics evidence or qualitative research evidence, describe search methods for identifying such studies. | N/A |
| Search strategies for bibliographic databases | ER31 | Present the exact search strategy (or strategies) used for each database in an Appendix, including any limits and filters used, so that it could be replicated. | S2 |
| Search strategies for other sources | ER32 | Report the search terms used to search any sources other than bibliographic databases (e.g., trials registers, the web, direct contact with primary study authors), and the dates of the searches. | N/A |
| Inclusion decisions | ER33 | State how inclusion decisions were made (i.e. from search results to included studies), clarifying how many people were involved and whether they worked independently. | 8 |
| Data collection process | ER34 | State how data were extracted from reports of included studies, clarifying how many people were involved (and whether independently), and how disagreements were handled. Describe data collection process for any reports requiring translation. | 9 |
| Requests for data | ER35 | Describe attempts to obtain or clarify data from individuals or organizations. | N/A |
| Data items | ER36 | List the types of information that were sought from reports of included studies. | 9, S4 |
| Tools to assess risk of bias/ study quality/ confidence in individual studies | ER37 | State the tool(s) or coding strategies used to assess the primary study quality/risk of bias/confidence for included studies, how the tool(s) or coding strategies were implemented, and the criteria used to assign studies, for example, to judgments of low risk, high risk, and unclear risk of bias; low quality or high quality. | 9 |
| Flow of studies | ER38 | Provide information on the flow of studies from the number(s) of references identified in the search to the number of studies included in the EGM, ideally using a flow chart. Clarify how multiple references for the same study relate to the individual studies. | 13 |
| Excluded studies | ER39 | List in the report key excluded studies (i.e., those a reader might reasonably have expected to find) and provide justification for each exclusion. | 13, S5 |
| Studies awaiting classification | ER40 | List in the report the characteristics of any studies that have been identified as potentially eligible but have not been incorporated into the map. | 13, S6 |
| Provide details of references | ER41 | In the map provide a link to full reference for each included study, including multiple citations if applicable | 13-19 |
| Included studies | ER42 | Provide a brief structured abstract of all included studies. This should include the characteristics of the study design, objectives and dimensions of the map. | 13-14 |
| Filter for selected characteristics of included studies | ER43 | Provide a filter for included studies to enable a user of the EGM to assess the availability of evidence for their own setting (e.g. region, study date, population characteristics) | N/A |
| Quality or confidence of systematic reviews | ER44 | All systematic reviews should be appraised for quality or confidence. | 18, S8 |
| ‘Risk of bias’, study quality of confidence coding | ER45 | Use a coding (colours or shapes) to indicate the risk of bias, study quality or confidence for each included study, with judgments about risks of bias. | 18, S8 |
| ‘Risk of bias’, study or confidence table | ER46 | Present a “risk of bias and/or study quality or confidence” table for each included study, with judgments about risks of bias, and explicit supports for these judgments. | 14, 15, S8 |
| Summarise quality of systematic reviews | ER47 | Provide a brief narrative summary of the quality or confidence of systematic reviews in results. | 15 |
| Summary assessments of risk of bias/ study quality | ER48 | Summarize the study quality/risk of bias by dimensions of the map. | N/A |
| Number of Tables and Figures | ER49 | Restrict the number of Tables and Figures to a small number (six or less) to convey key findings without affecting the readability of the EGM report text. | 13, 15, 18, 19 |
| Consistency of reporting | ER50 | Ensure that all evidence reported in the map is captured in the EGM report, and that the report text is consistent with all Tables and Figures | 13-19 |
| Assessments of the quality of the body of evidence | ER51 | Provide justification or rationale for any measures of the quality of the body of evidence for each key outcome. | 13-19 |
| Discussion headings | ER52 | Include in the report the standard headings when writing the Discussion. | 19-23 |
| Limitations | ER53 | In the report, discuss limitations of the map (e.g., incomplete identification of studies, reporting bias), and the implications of any study-level or outcome-level risk of bias/quality/ confidence assessments of the evidence in the map. | 21-22 |
| Conclusions: implications for research | ER54 | If recommending further research, structure the implications for research to address the nature of evidence required, including population, intervention comparison, outcome, and type of study. | 22-23 |
| Acknowledgements | ER55 | Acknowledge the contribution of people not listed as authors of the map, including any assistance from Campbell Coordinating Groups, non-author contributions to searching, data collection, study appraisal or statistical analysis, and the role of any funders. | 23 |
| Contributions of authors | ER56 | Describe the contributions of each author. | 24 |
| Declarations of interests | ER56 | Report any present or past affiliations or other involvement in any organization or entity with an interest in the map’s findings that might lead to a real or perceived conflict of interest. | 23 |
| Changes from the protocol | ER57 | Explain and justify any changes from the protocol (including any *post hoc* decisions about eligibility criteria. | 5, S1 |
| Sources of support | ER58 | List sources of financial and non-financial support for the map and the role of the funder, if any. | 2, 23-24 |
| EGM: evidence gap map | | | |
