# Supplementary material for: Interventions for treating obstetric fistula: An evidence gap map
Source: PLOS Glob Public Health. 2023 Jan 26;3(1):e0001481. doi: 10.1371/journal.pgph.0001481 (PMC10021774; doi:10.1371/journal.pgph.0001481)
Supplement: S2 Table — (DOCX) [file pgph.0001481.s004.docx]

**S2 Table: Finalised search strategies**

| \| **Ovid MEDLINE(R) and Epub Ahead of Print, In-Process, In-Data-Review & Other Non-Indexed Citations, Daily and Versions(R) 1946 to February 15, 2022: searched 16 February 2022** \| \| --- \| | | |
| --- | --- | --- | --- |
| **Line** | **Query** | **Search results** |
| 1 | Vaginal Fistula/ | 1449 |
| 2 | Vesicovaginal Fistula/ | 2535 |
| 3 | Rectovaginal Fistula/ | 1591 |
| 4 | Urinary Fistula/ | 3954 |
| 5 | (bladder adj2 fistula*).tw. | 324 |
| 6 | (vesic* adj2 fistula*).tw. | 3266 |
| 7 | (vagina* adj2 fistula*).tw. | 1428 |
| 8 | (urin* adj2 fistula*).tw. | 1213 |
| 9 | (ureterovaginal* adj2 fistula*).tw. | 282 |
| 10 | (urogenital adj2 fistula*).tw. | 242 |
| 11 | (genitourin* adj2 fistula*).tw. | 119 |
| 12 | (urethra* adj2 fistula*).tw. | 896 |
| 13 | (cystovagina* adj2 fistula*).tw. | 2 |
| 14 | (vagino* adj2 fistula*).tw. | 49 |
| 15 | (rectovaginal* adj2 fistula*).tw. | 1380 |
| 16 | (vaginorect* adj2 fistula*).tw. | 2 |
| 17 | (obstetric adj2 fistula*).tw. | 591 |
| 18 | (postobstetric adj2 fistula*).tw. | 1 |
| 19 | (pelvic adj2 fistula*).tw. | 192 |
| 20 | (circumferential adj2 fistula*).tw. | 25 |
| 21 | (recto* adj2 fistula*).tw. | 2762 |
| 22 | vvf.tw. | 360 |
| 23 | (urethro* adj2 fistula*).tw. | 1212 |
| 24 | Or/1-23 | 13,634 |
| 25 | Randomized controlled trials as Topic/ | 152,779 |
| 26 | Randomized controlled trial/ | 558,739 |
| 27 | Random allocation/ | 106,610 |
| 28 | Double blind method/ | 170,052 |
| 29 | Single blind method/ | 31,602 |
| 30 | Clinical trial/ | 533,849 |
| 31 | exp Clinical Trials as Topic/ | 370,469 |
| 32 | or/25-31 | 1,265,709 |
| 33 | (clinic$ adj trial$1).tw. | 425,676 |
| 34 | ((singl$ or doubl$ or treb$ or tripl$) adj (blind$3 or mask$3)).tw. | 186,319 |
| 35 | Placebos/ | 35,884 |
| 36 | Placebo$.tw. | 233,011 |
| 37 | Randomly allocated.tw. | 32,913 |
| 38 | (allocated adj2 random).tw. | 808 |
| 39 | or/33-38 | 712,499 |
| 40 | 32 or 39 | 1,582,399 |
| 41 | Case report.tw. | 355,232 |
| 42 | Letter/ | 1,170,357 |
| 43 | Historical article/ | 367,661 |
| 44 | Review of reported cases.pt. | 0 |
| 45 | Review, multicase.pt. | 0 |
| 46 | or/41-45 | 1,875,711 |
| 47 | 40 not 46 | 1,544,874 |
| 48 | Epidemiologic studies/ | 9001 |
| 49 | exp case control studies/ | 1,285,238 |
| 50 | exp cohort studies/ | 2,297,294 |
| 51 | Case control.tw. | 140,841 |
| 52 | (cohort adj (study or studies)).tw. | 262,690 |
| 53 | Cohort analy$.tw. | 9985 |
| 54 | (Follow up adj (study or studies)).tw. | 52,965 |
| 55 | (observational adj (study or studies)).tw. | 135,219 |
| 56 | Longitudinal.tw. | 285,418 |
| 57 | Retrospective.tw. | 642,902 |
| 58 | Cross sectional.tw. | 435,474 |
| 59 | Cross-sectional studies/ | 411,555 |
| 60 | or/48-59 | 3,453,529 |
| 61 | systematic review.ti. or meta-analysis.pt. or meta-analysis.ti. or systematic literature review.ti. or this systematic review.tw,kf,hw. or pooling project.tw,kf,hw. or (systematic review.ti,ab. and review.pt.) or meta synthesis.ti. or meta-analy*.ti. or integrative review.tw,kf,hw. or integrative research review.tw,kf,hw. or rapid review.tw,kf,hw. or umbrella review.tw,kf,hw. or consensus development conference.pt. or practice guideline.pt. or drug class reviews.ti. or (1469-493X or 1361-6137).is. or (1539-8560 or 1056-8751).is. or (2046-4924 or 1366-5278).is. or 1530-440X.is. or 2202-4433.is. | 381,595 |
| 62 | (clinical guideline and management).tw,kf,hw. or ((evidence based.ti. or exp evidence-based medicine/ or best practice*.ti. or evidence synthesis.ti,ab.) and (review.pt. or exp diseases non mesh/ or exp "behavior and behavior mechanisms"/ or exp therapeutics/ or evaluation studies.pt. or validation studies.pt. or guideline.pt. or pmcbook.af.)) | 80,596 |
| 63 | (systematic or systematically).tw,kf,hw. or critical.ti,ab. or study selection.tw,kf,hw. or ((predetermined or inclusion) and criteri*).tw,kf,hw. or exclusion criteri*.tw,kf,hw. or main outcome measures.tw,kf,hw. or standard of care.tw,kf,hw. or standards of care.tw,kf,hw. | 1,616,309 |
| 64 | (survey or surveys).ti,ab. or overview*.tw,kf,hw. or review.ti,ab. or reviews.ti,ab. or search*.tw,kf,hw. or handsearch.tw,kf,hw. or analysis.ti. or critique.ti,ab. or appraisal.tw,kf,hw. or (reduction.tw,kf,hw. and (exp risk/ or risk.tw,kf,hw.) and (exp "death"/ or "death".af. or (exp "recurrence"/ or "recurrence".af.))) | 3,916,467 |
| 65 | (literature or articles or publications or publication or bibliography or bibliographies or published).ti,ab. or pooled data.tw,kf,hw. or unpublished.tw,kf,hw. or citation.tw,kf,hw. or citations.tw,kf,hw. or database.ti,ab. or internet.ti,ab. or textbooks.ti,ab. or references.tw,kf,hw. or scales.tw,kf,hw. or papers.tw,kf,hw. or datasets.tw,kf,hw. or trials.ti,ab. or meta-analy*.tw,kf,hw. or (clinical and studies).ti,ab. or exp treatment outcome/ or treatment outcome.tw,kf,hw. or pmcbook.af. | 4,159,298 |
| 66 | (letter or newspaper article).pt. | 1,188,633 |
| 67 | 61 or 62 | 449,029 |
| 68 | 63 and 64 and 65 | 390,196 |
| 69 | 67 or 68 | 580,712 |
| 70 | 69 not 66 | 569,301 |
| 71 | 47 or 60 or 70 | 5,025,679 |
| 72 | 24 and 71 | 3496 |
| Lines 25-70 are study design filters taken from the Ovid Expert Search website | | |

| **Embase 1974 to 2022 February 15: searched 16 February 2022** | | |
| --- | --- | --- |
| **Line** | **Query** | **Search results** |
| 1 | cystovaginal fistula/ | 3220 |
| 2 | urinary tract fistula/ | 2636 |
| 3 | bladder fistula/ | 1212 |
| 4 | ureter fistula/ | 866 |
| 5 | urethra fistula/ | 2042 |
| 6 | rectovaginal fistula/ | 3272 |
| 7 | Vvf.tw | 771 |
| 8 | (bladder adj2 fistula*).tw. | 462 |
| 9 | (vesic* adj2 fistula*).tw. | 3865 |
| 10 | (vagina* adj2 fistula*).tw. | 1998 |
| 11 | (urin* adj2 fistula*).tw. | 1654 |
| 12 | (ureterovaginal* adj2 fistula*).tw. | 371 |
| 13 | (urogenital adj2 fistula*).tw. | 276 |
| 14 | (genitourin* adj2 fistula*).tw. | 228 |
| 15 | (urethra* adj2 fistula*).tw. | 1230 |
| 16 | (cystovagina* adj2 fistula*).tw. | 3 |
| 17 | (vagino* adj2 fistula*).tw. | 69 |
| 18 | (rectovaginal* adj2 fistula*).tw. | 2054 |
| 19 | (vaginorect* adj2 fistula*).tw. | 5 |
| 20 | (obstetric adj2 fistula*).tw. | 821 |
| 21 | (postobstetric adj2 fistula*).tw. | 2 |
| 22 | (pelvic adj2 fistula*).tw. | 246 |
| 23 | (circumferential adj2 fistula*).tw. | 35 |
| 24 | (recto* adj2 fistula*).tw. | 3980 |
| 25 | (urethra* adj2 fistula*).tw | 1230 |
| 26 | Or/1-25 | 17,165 |
| 27 | exp Meta Analysis/ | 238,701 |
| 28 | ((meta adj analy$) or metaanalys$).tw. | 290,513 |
| 29 | (systematic adj (review$1 or overview$1)).tw. | 284,384 |
| 30 | or/27-29 | 481,574 |
| 31 | cancerlit.ab. | 743 |
| 32 | cochrane.ab. | 140,562 |
| 33 | embase.ab. | 155,027 |
| 34 | (psychlit or psyclit).ab. | 1006 |
| 35 | (psychinfo or psycinfo).ab. | 45,904 |
| 36 | (cinahl or cinhal).ab. | 43,616 |
| 37 | science citation index.ab. | 3972 |
| 38 | bids.ab. | 755 |
| 39 | or/31-38 | 240,294 |
| 40 | reference lists.ab. | 21,864 |
| 41 | bibliograph$.ab. | 26,039 |
| 42 | hand-search$.ab. | 9562 |
| 43 | manual search$.ab. | 6274 |
| 44 | relevant journals.ab. | 1519 |
| 45 | or/40-44 | 58,814 |
| 46 | data extraction.ab. | 32,814 |
| 47 | selection criteria.ab. | 41,269 |
| 48 | 46 or 47 | 71,595 |
| 49 | review.pt. | 2,858,177 |
| 50 | 48 and 49 | 33,743 |
| 51 | letter.pt. | 1,210,944 |
| 52 | editorial.pt. | 717,299 |
| 53 | animal/ | 1,556,541 |
| 54 | human/ | 23,128,691 |
| 55 | 53 not (53 and 54) | 1,140,534 |
| 56 | Or/51-52,55 | 3,050,806 |
| 57 | 30 or 39 or 45 or 50 | 570,134 |
| 58 | 57 not 56 | 555,296 |
| 59 | Clinical trial/ | 1,027,610 |
| 60 | Randomized controlled trial/ | 695,760 |
| 61 | Randomization/ | 93,017 |
| 62 | Single blind procedure/ | 45,161 |
| 63 | Double blind procedure/ | 192,260 |
| 64 | Crossover procedure/ | 69,433 |
| 65 | Placebo/ | 376,802 |
| 66 | Randomi?ed controlled trial$.tw. | 277,871 |
| 67 | Rct.tw. | 45,451 |
| 68 | Random allocation.tw. | 2322 |
| 69 | Randomly allocated.tw. | 40,547 |
| 70 | Allocated randomly.tw. | 2761 |
| 71 | (allocated adj2 random).tw. | 915 |
| 72 | Single blind$.tw. | 28,335 |
| 73 | Double blind$.tw. | 227,525 |
| 74 | ((treble or triple) adj blind$).tw. | 1509 |
| 75 | Placebo$.tw. | 338,565 |
| 76 | Prospective study/ | 745,565 |
| 77 | or/59-76 | 2,498,793 |
| 78 | Case study/ | 83,754 |
| 79 | Case report.tw. | 476,726 |
| 80 | Abstract report/ or letter/ | 1,227,432 |
| 81 | or/78-80 | 1,775,120 |
| 82 | 77 not 81 | 2,428,639 |
| 83 | Randomized controlled trials/ | 220,389 |
| 84 | 82 or 83 | 2,552,213 |
| 85 | Clinical study/ | 157,244 |
| 86 | Case control study/ | 184,154 |
| 87 | Family study/ | 25,379 |
| 88 | Longitudinal study/ | 167,819 |
| 89 | Retrospective study/ | 1,201,892 |
| 90 | Prospective study/ | 745,565 |
| 91 | Randomized controlled trials/ | 220,389 |
| 92 | 90 not 91 | 736,966 |
| 93 | Cohort analysis/ | 806,689 |
| 94 | (Cohort adj (study or studies)).mp. | 387,382 |
| 95 | (Case control adj (study or studies)).tw. | 151,362 |
| 96 | (follow up adj (study or studies)).tw. | 68,406 |
| 97 | (observational adj (study or studies)).tw. | 209,720 |
| 98 | (epidemiologic$ adj (study or studies)).tw. | 114,571 |
| 99 | (cross sectional adj (study or studies)).tw. | 278,012 |
| 100 | or/85-89,92-99 | 3,321,567 |
| 101 | 58 or 84 or 100 | 5,276,344 |
| 102 | 26 and 101 | 4073 |
| Lines 27-82 and 85-100 are study design filters taken from the Ovid Expert Search website | | |

| **CENTRAL: searched 16 February 2022** | | |
| --- | --- | --- |
| **Line** | **Query** | **Search results** |
| 1 | MeSH descriptor: [Vaginal Fistula] explode all trees | 26 |
| 2 | MeSH descriptor: [Vesicovaginal Fistula] explode all trees | 11 |
| 3 | MeSH descriptor: [Rectovaginal Fistula] explode all trees | 13 |
| 4 | MeSH descriptor: [Urinary Fistula] explode all trees | 28 |
| 5 | #1 or #2 or #3 or #4 | 42 |
| 6 | (bladder NEAR/2 fistula*):ti,ab,kw | 14 |
| 7 | (vesic* NEAR/2 fistula*):ti,ab,kw | 46 |
| 8 | (vagina* NEAR/2 fistula*):ti,ab,kw | 58 |
| 9 | (urin* NEAR/2 fistula*):ti,ab,kw | 71 |
| 10 | (ureterovaginal* NEAR/2 fistula*):ti,ab,kw | 5 |
| 11 | (urogenital* NEAR/2 fistula*):ti,ab,kw | 4 |
| 12 | (genitourin* NEAR/2 fistula*):ti,ab,kw | 6 |
| 13 | (urethra* NEAR/2 fistula*):ti,ab,kw | 52 |
| 14 | (cystovagina* NEAR/2 fistula*):ti,ab,kw | 27 |
| 15 | (vagino* NEAR/2 fistula*):ti,ab,kw | 0 |
| 16 | (rectovaginal* NEAR/2 fistula*):ti,ab,kw | 83 |
| 17 | (vaginorect* NEAR/2 fistula*):ti,ab,kw | 0 |
| 18 | (obstetric NEAR/2 fistula*):ti,ab,kw | 17 |
| 19 | (postobstetric NEAR/2 fistula*):ti,ab,kw | 0 |
| 20 | (pelvic NEAR/2 fistula*):ti,ab,kw | 4 |
| 21 | (circumferential NEAR/2 fistula*):ti,ab,kw | 2 |
| 22 | (recto* NEAR/2 fistula*):ti,ab,kw | 106 |
| 23 | (uretho* NEAR/2 fistula*):ti,ab,kw | 0 |
| 24 | (vvf):ti,ab,kw | 22 |
| 25 | #6 or #7 or #8 or #9 or #10 or #11 or #12 or #13 or #14 or #15 or #16 or #17 or #18 or #19 or #20 or #21 or #22 or #23 or #24 | 312 |
| 26 | #5 or #25 | 312 |

| **Global Index Medicus (All Regions): searched 16 February 2022** | | |
| --- | --- | --- |
| **Line** | **Query** | **Search results** |
| 1 | Vaginal Fistula/ OR Vesicovaginal Fistula/ OR Rectovaginal Fistula/ OR Urinary Fistula/ | 516 |
| 2 | vaginal fistula*.tw. | 539 |
| 3 | vesicovaginal fistula*.tw. | 354 |
| 4 | rectovaginal fistula*.tw. | 238 |
| 5 | urinary fistula*.tw. | 1177 |
| 6 | urethro vaginal fistula*.tw. | 6 |
| 7 | urethra vaginal fistula*.tw. | 40 |
| 8 | obstetric fistula*.tw. | 111 |
| 9 | pelvic floor fistula*.tw. | 24 |
| 10 | ureterovaginal fistula*.tw. | 63 |
| 11 | circumferential fistula*.tw. | 53 |
| 12 | vesicocervical fistula*.tw. | 10 |
| 13 | vesico cervical fistula*.tw. | 37 |
| 14 | 1 OR 2 OR 3 OR 4 OR 5 OR 6 OR 7 OR 8 OR 9 OR 10 OR 11 OR 12 OR 13 | 1743 |

| **ScanMedicine: searched 16 February 2022** | | |
| --- | --- | --- |
| **Line** | **Query** | **Search results** |
| 1 | "obstetric fistula"\|"vesicovaginal fistula"\|"rectovaginal fistula"\|"urethrovaginal fistula"\|"obstetric fistulas"\|"vesicovaginal fistulas"\|"rectovaginal fistulas"\|"urethrovaginal fistulas" | 75 |
