# Supplementary material for: Interventions for treating obstetric fistula: An evidence gap map
Source: PLOS Glob Public Health. 2023 Jan 26;3(1):e0001481. doi: 10.1371/journal.pgph.0001481 (PMC10021774; doi:10.1371/journal.pgph.0001481)
Supplement: S3 Table — (DOCX) [file pgph.0001481.s005.docx]

**S3 Table: Full eligibility criteria for the evidence map**

|  | Included | Excluded |
| --- | --- | --- |
| Study design | - RCTs (parallel, cross-over, cluster, multi-arm) - Case-control studies - Cohort studies (prospective and retrospective) - Systematic reviews (containing RCTs and/or non-randomised studies) | - Single-arm studies (i.e. not intervention vs intervention, intervention vs control or analysing the effects of an intervention on two different subgroups) - Case reports - Cross-sectional studies - Qualitative studies - Systematic reviews of qualitative studies - Mixed-methods studies (containing both quantitative and qualitative elements) |
| Population | - Women of any age - Living in any country or setting - Studies where more 80% or more of the fistula cases are caused by obstetric means (e.g. delayed or difficult labour, or caesarean section leading to “low” fistula formation (i.e. most likely to be mid-vaginal or bladder neck fistula of obstetric origin)) - As long as at least 80% of the fistula cases were caused due to obstetric origin, any type were eligible (e.g. vesicovaginal, rectovaginal, urethrovaginal, juxtacervical, uterine, or any studies only describing the fistula as “obstetric” with no other definition) | - Women whose fistula are not defined as being as the result of pregnancy or childbirth (e.g. iatrogenic fistula, fistula caused by hysterectomy or radiation therapy) - Studies where less than 80% of the fistula cases are caused by obstetric means (e.g. delayed or difficult labour, or caesarean section leading to “low” fistula formation (i.e. most likely to be mid-vaginal or bladder neck fistula of obstetric origin) - Studies where the type of fistula is not clearly defined (i.e. unclear whether iatrogenic or obstetric) - If caesarean section leads to “high” fistula formation (i.e. most likely to be vesicocervical, vesico-uterine, ureterocervical, uretero-uterine or juxtacervical vesicovaginal fistula of iatrogenic origin), studies excluded if these cases mean that less than 80% of the participants had obstetric fistula |
| Intervention | - Lifestyle interventions - Skin protection - Pads - Urethral plugs - Vaginal lubricants - UTI prophylaxis - Dietary modification - Catheter insertion - Bladder catheterisation - Ureteral catheterisation - Physical therapy - Therapeutic exercise - Bladder training - Bowel habit training - Muscle training - Coordination training - Biofeedback - Electrical muscle stimulation - Psychological therapy   - CBT - Surgical intervention - Native tissue repair - Graft repair - Tissue flaps - Native tissue repair, graft repair or tissue flaps with anal sphincter repair - Urinary diversion surgeries (e.g. ileal conduit, Mainz pouch) - Colostomy (temporary or permanent) for rectal fistula repairs - Debridement of fistula - Cystoscopic cauterisation of fistula | - Preventative interventions for obstetric fistula (e.g. education) - Interventions for reintegration after treatment or aftercare for surgery - Studies of surgical interventions where the approach and not the type of surgery is described (e.g. studies describing vaginal and abdominal approaches to fistula repair without clarification of the type surgery listed) |
| Comparison | - Studies comparing one or more of the interventions listed above with another intervention or a control intervention - Studies comparing the effects of an intervention among two or more population subgroups (e.g. differing time to delivery, differing age, geographical location) - Systematic reviews including studies with the comparators listed above | - Single-arm primary studies (i.e. not intervention vs intervention, intervention vs control or analysing the effects of an intervention on one subgroup versus another) |
| Outcomes | - Any outcomes eligible so long as the study or systematic review meets the rest of the PICOS eligibility criteria | - N/A |
| Publication type | - Journal articles | - Books or book chapters - Editorials, commentaries or letters |
