# Supplementary material for: Interventions for treating obstetric fistula: An evidence gap map
Source: PLOS Glob Public Health. 2023 Jan 26;3(1):e0001481. doi: 10.1371/journal.pgph.0001481 (PMC10021774; doi:10.1371/journal.pgph.0001481)
Supplement: S4 Table — (DOCX) [file pgph.0001481.s006.docx]

**S4 Table: Coding tool for evidence map**

| Study ID | Free text box | - Name of first author - Year published |
| --- | --- | --- |
| Study design | Tick boxes and free text for additional detail | - RCT (additional information: parallel-arm, cross-over, cluster) - Non-RCT (additional information: case-control study, cohort study) - Systematic review (additional information: RCTs only, non-RCTs only, or combination of RCTs and non-RCT) |
| Funding sources | Free text box | - As reported in the text |
| Conflicts of interest | Free text box | - As reported in the text |
| Type of fistula | Tick boxes and additional detail | - Single type of fistula - Mixed fistulas (e.g. vesicovaginal, urethrovaginal or rectovaginal) - Type of obstetric fistula not defined |
| Population | Free text boxes | - Country - Setting - Dates study conducted - Number of participants in study (primary studies only; N/A if systematic review) - Number of studies in systematic review (systematic review only; additional details could include amount of RCTs and non-RCTs included; N/A if primary study) - Stage or classification of fistula included |
|  | Tick boxes | - More than 80% women with obstetric fistula - More than 95% women with obstetric fistula - Unclear (used when fistulae included are described as obstetric in the title or elsewhere but aetiology not fully described) |
| Intervention | Tick boxes | - Lifestyle management   - Skin protection   - Pads   - Urethral plugs   - Vaginal lubricants   - UTI prophylaxis   - Dietary modification - Catheter insertion   - Bladder catheterisation   - Ureteral catheterisation - Physical therapy   - Therapeutic exercise   - Bladder training   - Bowel habit training   - Muscle training   - Coordination training   - Biofeedback   - Electrical muscle stimulation - Psychological therapy   - CBT - Surgical intervention   - Native tissue repair   - Graft repair   - Tissue flaps   - Native tissue repair, graft repair or tissue flaps with anal sphincter repair   - Urinary diversion surgeries (e.g. ileal conduit, Mainz pouch)   - Colostomy (temporary or permanent) for rectal fistula repairs   - Debridement of fistula   - Cystoscopic cauterisation of fistula |
| Interventions reported separately | Tick boxes | - Yes - No |
| Comparison | Tick boxes and additional detail | - Intervention versus intervention and/or control - Comparison of population subgroups |
| Outcomes | Tick boxes | - Cure or improvement of obstetric fistula - Improvement in pain - Improvement in urinary incontinence - Improvement in faecal incontinence - Improvement in difficulties urinating (e.g. slow stream, hesitancy) - Improvement in difficulties defecating (e.g. constipation, straining on the toilet) - Improvement in sexual function (e.g. less pain during sex) - Improvement in haematuria (blood in urine) - Improvement in quality of life - Improvement in mental health (e.g. anxiety or depression) - Improvement in associated pelvic organ prolapse symptoms - Improvement in urinary retention - Improvement in activities of daily living (everyday tasks) - Adverse events of interventions - Patient satisfaction with intervention - Adherence to the intervention - Woman’s need for further treatment |
| Length of follow-up | Free text boxes | - Primary studies: length of follow-up - Systematic reviews: time-points for assessment |
| Risk of bias | Tick boxes and free text | - AMSTAR-2 - Cochrane ‘Risk of bias’ tool - JBI Checklist for Cohort Studies - JBI Checklist for Case-Control Studies |
| Additional reports of study | Free text | - Bibliographic details of any additional reports of the study |
