# Supplementary material for: Interventions for treating obstetric fistula: An evidence gap map
Source: PLOS Glob Public Health. 2023 Jan 26;3(1):e0001481. doi: 10.1371/journal.pgph.0001481 (PMC10021774; doi:10.1371/journal.pgph.0001481)
Supplement: S5 Table — (DOCX) [file pgph.0001481.s007.docx]

**S5 Table: List of excluded studies**

| **Excluded from database searches** | |
| --- | --- |
| **Study reference** | **Reason for exclusion** |
| Abdel-Karim AM, Elmissiry M, Aboulfotoh A, Moussa A, Elsalmy S. Laparoendoscopic single-site surgery (LESS) and conventional laparoscopic extravesical repair of vesicouterine fistula: single-center experience. International Urology & Nephrology. 2013;45(4):995-1000. | Ineligible population: not described as obstetric fistula |
| Abdel-Karim A, Aboelfotoh A, Elsalmy S. Laparoscopic repair of female genitourinary fistulae: Single-center singlesurgeon experience. European Urology Supplements. 2017;16(16):e1724. | Ineligible population: numbers of iatrogenic and obstetric cases of fistula not reported |
| Abdel-Karim A, Elmissiry M, Moussa A, Mahfouz W, Abulfotooh A, Dawood W, et al. Laparoscopic repair of female genitourinary fistulae: 10-year single-center experience. International Urogynecology Journal. 2020;31(7):1357-62. | Ineligible population: less than 80% obstetric |
| Abdullah A, Syed S. Vesicovaginal fistula increasing problem in developing world. European Urology, Supplements. 2009;8:240. | Ineligible study design: focus on aetiology not intervention |
| Abou-Elela A, Alfaiomy H, Torky H, Reyad E, Azazy S. The use of rotational bladder flap and hemostatic matrix sealant (FloSeal): a modified transabdominal approach to repair supratrigonal and complex vesicovaginal fistula. Surgical Technology International. 2012;22:44-8. | Ineligible population: less than 80% obstetric |
| Ahmed S, Quaiyum A, Islam F, Genadry R. Delay in care seeking decreases success in obstetrical fistula repair. Female Pelvic Medicine and Reconstructive Surgery. 2013;2:S141. | Ineligible intervention: does not specify method of repair |
| Ahmed Z, Abdullahi H, Yola A, Yakasai I. Obstetrics fistula repairs in Kano, Northern Nigeria: The journey so far. Annals of Tropical Medicine and Public Health. 2013;6(5):545-8. | Ineligible intervention: does not specify method of repair |
| Aimakhu VE. Reproductive functions after the repair of obstetric vesicovaginal fistulae. Fertility & Sterility. 1974;25(7):586-91. | Ineligible intervention: does not specify method of repair |
| Aitola P, Hiltunen KM, Matikainen M. Fibrin glue in perianal fistulas--a pilot study. Annales Chirurgiae et Gynaecologiae. 1999;88(2):136‐8. | Ineligible study design: case series |
| Akhtar N, Khan SM. A tertiary experience of vesicovaginal fistula repair: With Naveed's technique. BJOG: An International Journal of Obstetrics and Gynaecology. 2017;124:194-5. | Ineligible study design: cross-sectional |
| Al Aridy HM. The use of peritoneal flap in the transabdominal repair of vesicovaginal fistula. The Iraqi Postgraduate Medical Journal. 2010;9:235-43. | Ineligible population: less than 80% obstetric |
| Al Otaibi K, Al Damanhori R. Minimally invasive treatment of ureterovaginal fistula: A review and report of a new technique. Journal of Endourology. 2013;1:A266. | Ineligible study design: case report |
| Al Rubai MJ, Abdul Kareem NF. Abdominal transperitonial approach in management of vesicovaginal fistula in Iraqi patients. The Iraqi Postgraduate Medical Journal. 2012;11(2):238-41. | Ineligible study design: single group |
| Al-Salihi S, Lim J, Carey M. Clinical and histological study of the utility of a seton drain in the surgical management of rectovaginal fistulae. International Urogynecology Journal and Pelvic Floor Dysfunction. 2009;20:S373-4. | Ineligible population: less than 80% obstetric |
| Alam MM, Awal MA, Rasul MA, Rahman MM, Naser MF, Salam MA, et al. Surgical management of rectourethral fistula in different situations. Mymensingh Medical Journal: MMJ. 2014;23(1):75-80. | Ineligible population: not obstetric fistula |
| Ali W, Kharal IA, Ijaz I, Younis M. Outcome of transvaginal vesicovaginal fistula repair with martius fat pad flap in comparison to simple closure. Pakistan Journal of Medical and Health Sciences. 2018;12(3):1126‐8. | Ineligible population: not described as obstetric fistula |
| Aliyu SU, Hanif SM, Lawal IU. Effect of Paula exercise method on functional outcomes of women with post fistula repair incontinence: a protocol for randomized controlled trial. BMC Women's Health. 2021;21(101). | Ineligible population: treating urinary incontinence in women after obstetric fistula, not obstetric fistula itself |
| Alper P, Ali E. LIFT technique for simple rectovaginal fistula. JCPSP Journal of the College of Physicians & Surgeons Pakistan. 2017;27(12):791-2. | Ineligible study design: case report |
| Alver O, Ersoy YE, Aydemir I, Erguney S, Teksoz S, Apaydin B, et al. Use of "house" advancement flap in anorectal diseases. World Journal of Surgery. 2008;32(10):2281-6. | Ineligible study design: single arm, no subgrouping or comparison |
| Ambauen-Berger B, Walker SH. Authors' reply re: Quality of life among women in Bangladesh following ileal conduit urinary diversion operations for irreparable vesicovaginal fistula and bladder exstrophy; observational study. BJOG: An International Journal of Obstetrics & Gynaecology. 2017;124(12):1909. | Ineligible publication type: letter |
| Anonymous. Ineffective surgery for adhesions. Medicine Today. 2003;4(6):8. | Ineligible population: chronic abdominal pain, not obstetric fistula |
| Anonymous. Reply by Authors. Journal of Urology. 2016;196:964-5. | Ineligible publication type: letter with no additional information |
| Ansquer Y, Mellier G, Santulli P, Bennis M, Mandelbrot L, Madelenat P, et al. Latzko operation for vault vesicovaginal fistula. Acta Obstetricia et Gynecologica Scandinavica. 2006;85(10):1248-51. | Ineligible population: iatrogenic fistula, not obstetric fistula |
| Anyikam A, Holden A. International variation in interpositional graft use for vesicovaginal fistula repair: A systematic review. International Urogynecology Journal and Pelvic Floor Dysfunction. 2011;2:S380. | Ineligible study design: not enough information to know if the included studies in this review meet the inclusion criteria |
| Archibong MS, Ayegbusi OE. Minimal access, optimal dryness: A review of laparoscopic repair of vesicovaginal fistula. World Journal of Laparoscopic Surgery. 2020;13(3):130-2. | Ineligible population: aetiology of fistula not described, cannot tell percentage with obstetric fistula |
| Aristide Kabore F, Kambou T, Ouattara A, Zango B, Yameogo C, Kirakoya B, et al. Epidemiology, etiology and psychosocial impact of urogenital fistulas in a cohort of 170 consecutive patients managed in three treatment centers in Burkina Faso from 2010 to 2012 [French]. Progres en urologie : journal de l'Association francaise d'urologie et de la Societe francaise d'urologie. 2014;24(8):526-32. | Ineligible study design: not interventional; examines the epidemiology, aetiology and impact of fistula |
| Arrowsmith SD. Genitourinary reconstruction in obstetric fistulas. Journal of Urology. 1994;152(2):403-6. | Ineligible study design: single group |
| Arrowsmith SD. Urinary diversion in the vesico-vaginal fistula patient: general considerations regarding feasibility, safety, and follow-up. International Journal of Gynaecology & Obstetrics. 2007;99:S65-8. | Ineligible study design: reports experience, not a clinical trial |
| Asif Abbas K, Riaz A, Rafique A. Role of Peritoneum as interposition tissue in the management of vesico vaginal fistula. Journal of Surgery Pakistan. 2005;10:2-4. | Ineligible population: iatrogenic fistula, not obstetric fistula |
| Asif Nawaz MM, Ghaffar A, Ghaffar U, Javeed Z. Surgical management of vesicovaginal fistula, single center experience at urology department Nishtar Medical University Hospital Multan. Medical Forum Monthly. 2019;30(10):36-40. | Ineligible intervention: only described as vaginal or abdominal repair with no further specification |
| Atwa A, Rawal A, Joshi P, Kulkarni S, Bhadranavar S. A novel technique for repair of urethroperineal fistula complicating abdominoperineal resection of rectal carcinoma. Journal of Urology. 2019;201:e974. | Ineligible study design: single arm, no subgrouping or comparison |
| Australian New Zealand Clinical Trials Registry (ANZCTR). Randomised controlled trial comparing anal fistula closure rates between anal fistula plug insertion and ligation of intersphincteric fistula tract in patients with complex anal fistula of cryptoglandular origin. Trial ID: ACTRN12611001167954. 2011. [Accessed 16 February 2022]. | Ineligible population: fistula of cryptoglandular origin, not obstetric |
| Australian New Zealand Clinical Trials Registry (ANZCTR). Are women with obstetric fistula shorter that those who had a vaginal delivery? Trial ID: ACTRN12617001072303. 2017. [Accessed 16 February 2022]. | Ineligible intervention: not an interventional study, does not look at treatment efficacy |
| Australian New Zealand Clinical Trials Registry (ANZCTR). The risk of domestic violence in women suffering with obstetric fistula, chronic 4th degree tear and pelvic organ prolapse. Trial ID: ACTRN12617001073392. 2017. [Accessed 16 February 2022]. | Ineligible study design: study of causative factors, not interventional |
| Australian New Zealand Clinical Trials Registry (ANZCTR). A Prospective Randomised Controlled Trial investigating the effect of optimised therapeutic infliximab levels guided by proactive therapeutic drug monitoring on fistula healing in adults with perianal fistulising Crohn's Disease: PROACTIVE Trial. Trial ID: ACTRN12621000023853. 2021. [Accessed 16 February 2022]. | Ineligible population: participants with Crohn's disease |
| Ayaz A, un Nisa R, Anwar S, Mohammad T. Vesicovaginal and rectovaginal fistulas: 12-year results of surgical treatment. Journal of Ayub Medical College, Abbottabad: JAMC. 2012;24(3):25-7. | Ineligible intervention: only described as vaginal or abdominal repair with no further specification |
| Ayed M, El Atat R, Hassine LB, Sfaxi M, Chebil M, Zmerli S. Prognostic factors of recurrence after vesicovaginal fistula repair. International Journal of Urology. 2006;13(4):345-9. | Ineligible population: less than 80% obstetric |
| Ayhan A, Tuncer ZS, Dogan L, Pekin S, Kisnisci HA. Results of treatment in 182 consecutive patients with genital fistulas. International Journal of Gynaecology & Obstetrics. 1995;48(1):43-7. | Ineligible population: less than 80% obstetric |
| Bahuguna G, Mittal A, Kumar Panwar V, Singh Bhadoria A, Kumar Mandal A. Management strategies and outcome of ureterovaginal fistulae: A systematic review and meta-analysis. BJOG: An International Journal of Obstetrics and Gynaecology. 2021;128:267-8. | Ineligible population: less than 80% obstetric |
| Baloch BA, Salam A, Unnisa Z, Nawaz H. Vesico-vaginal fistulae; review of the causes, diagnosis and treatment. The Professional Medical Journal. 2014;21(5):851-5. | Ineligible population: less than 80% obstetric |
| Baloch R, Hafeezullah, Jalbani M, Denari R. Vesicovaginal fistulae: a surgical outcome. Journal of Surgery Pakistan. 2003;8(1):10-3. | Ineligible study design: single group |
| Bangser M. Strengthening public health priority-setting through research on fistula, maternal health, and health inequities. International Journal of Gynecology and Obstetrics. 2007;99:S16-S20. | Ineligible study design: description of four studies, only one about repair and not described by groups |
| Bannura GC, Barrera AE, L. MC, F. IF, Gallardo CV. Alternativas quirúrgicas en el manejo de la fístula rectovaginal: experiencia de 25 años. Revista Chilena de Cirugía. 2017;69(2):144-50. | Ineligible population: less than 80% obstetric |
| Banura G, Contreras J, Melo C, Barrera A. Espectro clínico de la fístula rectovaginal: análisis de 38 pacientes. Revista Chilena de Obstetricia y Ginecología. 2002;67(3):219-25. | Ineligible population: not obstetric fistula |
| Barnfield L, Mahran M, Katimada-Annaiah T, Esmyot M, Cooke L, Vusirikala K, et al. Re: Use of a postoperative pad test to identify continence status in women after obstetric vesicovaginal fistula repair: a prospective cohort study. BJOG: An International Journal of Obstetrics & Gynaecology. 2017;124(10):1622-3. | Ineligible publication type: letter |
| Barone MA, Frajzyngier V, Ruminjo J, Asiimwe F, Barry TH, Bello A, et al. Determinants of postoperative outcomes of female genital fistula repair surgery. Obstetrics & Gynecology. 2012;120(3):524-31. | Ineligible intervention: does not specify repair type |
| Barratt R, Kotes S, Pakzad M, Hamid R, Ockrim J, Greenwell T. The outcomes of urethrovaginal fistula repair. Journal of Clinical Urology. 2018;11:87. | Ineligible population: less than 80% obstetric |
| Batra R, Singh A, Ganpule A, Sabnis R, Desai M. Robotic assisted vesico-vaginal fistula repair: Our technique and outcomes. Journal of Endourology. 2021;35:A81. | Ineligible population: less than 80% obstetric |
| Bazi T. Spontaneous closure of vesicovaginal fistulas after bladder drainage alone: review of the evidence. International Urogynecology Journal. 2007;18(3):329-33. | Ineligible population: less than 80% obstetric |
| Bazi T, Nasr R. Management of vesicovaginal fistulae: A multicenter analysis from the fellows' pelvic research network. Female Pelvic Medicine and Reconstructive Surgery. 2015;21(1):59. | Ineligible population: less than 80% obstetric |
| Beardmore-Gray A, Pakzad M, Hamid R, Ockrim J, Greenwell T. Does the Goh classification predict the outcome of vesico-vaginal fistula repair in the developed world? International Urogynecology Journal. 2017;28(6):937-40. | Ineligible population: less than 80% obstetric |
| Bekele ML, Jijo ZW. Treatment outcome of obstetric fistula and associated factors in Yirgalem Fistula Center, Ethiopia. International Journal of Gynecology and Obstetrics. 2018;143:422. | Ineligible intervention: does not specify repair type |
| Belayihun B, Mavhandu-Mudzusi AH. Effects of surgical repair of obstetric fistula on severity of depression and anxiety in Ethiopia. BMC Psychiatry. 2019;19(1):58. | Ineligible intervention: type of surgery not specified |
| Bengtson AM, Kopp D, Tang JH, Chipungu E, Moyo M, Wilkinson J. Identifying Patients With Vesicovaginal Fistula at High Risk of Urinary Incontinence After Surgery. Obstetrics & Gynecology. 2016;128(5):945-53. | Ineligible intervention: specifies type of repair for preventing UI but not main method of obstetric fistula repair |
| Benski AC, Delavy M, Rochat CH, Viviano M, Catarino R, Elsig V, et al. Prognostic factors and long-term outcomes of obstetric fistula care using the Tanguieta model. International Journal of Gynecology & Obstetrics. 2020;148(3):331-7. | Ineligible intervention: repair only described as vaginal or abdominal, no other details |
| Bernard L, Giles A, Fabiano S, Giles S, Hudgins S, Olson A, et al. Predictors of Obstetric Fistula Repair Outcomes in Lubango, Angola. Journal of Obstetrics & Gynaecology Canada: JOGC. 2019;41(12):1726-33. | Ineligible study design: two cohorts compared against one another; not a valid study design |
| Berthe HJG, Cisse MC, Diakite AS, Diakite ML, Diallo MS, Ouatarra Z, et al. Etude des facteurs influencant les resultats cliniques de la chirurgie de la fistule uro-genitale obstetricale au CHU de l'hopital du Point G Bamako Mali. Mali Médical (En Ligne). 2015;30(3):42-5. | Ineligible study design: cross-sectional |
| Bing-Shu L, Li H, Qin W, Min H, Yan-Xiang C. Clinical effects of transvaginal vesicovaginal fistula repair surgery mediated by the Foley catheter (64 cases). Clinical & Experimental Obstetrics & Gynecology. 2011;38(4):360-3. | Ineligible population: less than 80% obstetric |
| Bishinga A, Zachariah R, Hinderaker S, Tayler-Smith K, Khogali M, van Griensven J, et al. High loss to follow-up following obstetric fistula repair surgery in rural Burundi: is there a way forward? Public Health in Action. 2013;3(2):113-7. | Ineligible intervention: type of surgery not specified |
| Blaivas JG, Heritz DM, Romanzi LJ. Early versus late repair of vesicovaginal fistulas: vaginal and abdominal approaches. Journal of Urology. 1995;153(4):1110-2; discussion 2-3. | Ineligible population: not obstetric fistula |
| Borseth KF, Acharya G, Kiserud T, Trovik J. Incidence of gynecological fistula and its surgical treatment: A national registry-based study. Acta Obstetricia et Gynecologica Scandinavica. 2019;98(9):1120-6. | Ineligible population: fistula not described as obstetric |
| Bouya PA, Nganongo WI, Lomin D, Iloki LH. [Retrospective study of 34 urogenital fistulas of obstetricalal origin]. Gynecologie, Obstetrique & Fertilite. 2002;30(10):780-3. | Ineligible study design: single group |
| Bozdar HUR, Fatima S, Memon IU, Bhatti WS, Bhatti NK, Memon IK. Experience of management of vesicovaginal fistula in Khairpur medical college hospital Khairpur. Pakistan Journal of Medical and Health Sciences. 2021;15:1537-9. | Ineligible population: less than 80% obstetric |
| Brandt FT, Lorenzato FR, Albuquerque CD. Treatment of vesicovaginal fistula by bladder mucosa autograft technique. Journal of the American College of Surgeons. 1998;186(6):645-8. | Ineligible population: iatrogenic fistula, not obstetric fistula |
| Brazilian Registry of Clinical Trials (ReBEC). Epidemiological and demographic data collect from women Urinary Incontinence. Trial ID: RBR-8jd639. 2020. [Accessed 16 February 2022]. | Ineligible study design: development of a registry system |
| Brook G, Tessema AB. Obstetric fistula: the use of urethral plugs for the management of persistent urinary incontinence following successful repair. International Urogynecology Journal. 2013;24(3):479-84. | Ineligible population: study focuses on women who have urinary incontinence after obstetric fistula repair and not on obstetric fistula |
| Brough SJS, Barrington JW, Wheeler J, Stephenson TP. Omental interposition in the management of urinary fistulas involving the vagina. Journal of Obstetrics and Gynaecology. 1995;15(5):316-8. | Ineligible population: less than 80% obstetric |
| Browning A. The circumferential obstetric fistula: characteristics, management and outcomes. BJOG: An International Journal of Obstetrics & Gynaecology. 2007;114:1172-6. | Ineligible study design: compares one population undertaking an intervention with another population undertaken other interventions; not an eligible design |
| Browning A, Whiteside S. Characteristics, management, and outcomes of repair of rectovaginal fistula among 1100 consecutive cases of female genital tract fistula in Ethiopia. International Journal of Gynaecology & Obstetrics. 2015;131(1):70-3. | Ineligible population: only a small minority of rectovaginal fistulas included are described as obstetric; aetiology of remainder of included fistula cases not reported |
| Browning A, Fentahun W, Goh JT. The impact of surgical treatment on the mental health of women with obstetric fistula. BJOG: An International Journal of Obstetrics & Gynaecology. 2007;114(11):1439-41. | Ineligible study design: single group, pre-post |
| Browning A, Williams G, Petros P. Skin flap vaginal augmentation helps prevent and cure post obstetric fistula repair urine leakage: a critical anatomical analysis. BJOG: An International Journal of Obstetrics & Gynaecology. 2018;125(6):745-9. | Ineligible study design: describes surgical techniques |
| Bruce RG, El-Galley RE, Galloway NT. Use of rectus abdominis muscle flap for the treatment of complex and refractory urethrovaginal fistulas. Journal of Urology. 2000;163(4):1212-5. | Ineligible study design: case series |
| Buchanan GN, Bartram CI, Phillips RK, Gould SW, Halligan S, Rockall TA, et al. Efficacy of fibrin sealant in the management of complex anal fistula: a prospective trial. Diseases of the Colon & Rectum. 2003;46(9):1167-74. | Ineligible study design: case series |
| Bugeja R, Brown K, Harding C, Greenwell T. Remote mentorship as a novel training method in urogenital fistula surgery. Neurourology and Urodynamics. 2019;38:S134-5. | Ineligible population: iatrogenic fistula, not obstetric fistula |
| Byrnes JN, Gebhart J, Schmitt J, Mara K, Weaver AL, Chua HK, et al. Outcomes of recurrent rectovaginal fistula repair. Female Pelvic Medicine and Reconstructive Surgery. 2017;23(2):S62. | Ineligible population: fistula not described as obstetric |
| Byrnes JN, Schmitt JJ, Faustich BM, Mara KC, Weaver AL, Chua HK, et al. Outcomes of Rectovaginal Fistula Repair. Female Pelvic Medicine & Reconstructive Surgery. 2017;23(2):124-30. | Ineligible population: fistula not described as obstetric |
| Capes T, Stanford EJ, Romanzi L, Foma Y, Moshier E. Comparison of two classification systems for vesicovaginal fistula. International Urogynecology Journal and Pelvic Floor Dysfunction. 2012;23(12):1679-85. | Ineligible intervention: repair described as vaginal or abdominal with no further description |
| Carrara B, María C. Fístula recto-vaginal. In: Castillo P, Edgardo A, editors. Tratado de perineología: disfunciones del piso pélvico. Montevideo: Academia Nacional de Medicina; 2019. p. 219-28. | Ineligible publication type: book |
| Castille YJ, Avocetien C, Zaongo D, Colas JM, Peabody JO, Rochat CH. One-year follow-up of women who participated in a physiotherapy and health education program before and after obstetric fistula surgery. International Journal of Gynaecology & Obstetrics. 2015;128(3):264-6. | Ineligible study design: single group, pre-post design |
| Cavalcanti AG. Editorial comment: Urorectal fistula repair using different approaches: operative results and quality of life issues. International Brazilian Journal of Urology. 2021;47(2):413-4. | Ineligible publication type: editorial comment |
| Chakrabortti DK. Vesico-uterine fistula following caesarean section. Journal of the Indian Medical Association. 1991;89(12):341-2. | Ineligible study design: case report |
| Champagne BJ, McGee MF. Rectovaginal Fistula. Surgical Clinics of North America. 2010;90(1):69-82. | Ineligible study design: commentary |
| Chandna A, Mavuduru RS, Bora GS, Sharma AP, Parmar KM, Devana SK, et al. Robot-assisted Repair of Complex Vesicovaginal Fistulae: Feasibility and Outcomes. Urology. 2020;144:92-8. | Ineligible population: less than 80% obstetric |
| Chang OH, Bretschneider CE, Unger C. 17: Perioperative adverse events by route of surgery in patients undergoing rectovaginal fistula repair in the United States. American Journal of Obstetrics and Gynecology. 2019;220:S717. | Ineligible intervention: repair described as vaginal or abdominal with no further description |
| Chang OH, Ganesh P, Wilkinson JP, Pope RJ. Extended bladder catheterization for women with positive dye tests after obstetric vesicovaginal fistula repair surgery. International Journal of Gynaecology & Obstetrics. 2020;149(1):61-5. | Ineligible study design: risk factors to successful or unsuccessful extension, not interventional |
| Chang OH, Pope R, Ganesh P, Stokes M, Wilkinson JP. Extending the duration of bladder catheterization for women with positive dye tests after obstetric fistula surgery. Female pelvic medicine & reconstructive surgery. 2018;24(5):S129‐. | Ineligible study design: risk factors to successful or unsuccessful extension, not interventional |
| Chang OH, Pope RJ, Sangi-Haghpeykar H, Ganesh P, Wilkinson JP. Predictors of Urinary Retention After Vesicovaginal Fistula Surgery: A Retrospective Case-Control Study. Female Pelvic Medicine & Reconstructive Surgery. 2020;26(12):726-30. | Ineligible intervention: paper does not include eligible interventions |
| Chen C, Yin L. Retrospective research minimally invasive treatment of mid-low rectovaginal fistula. Diseases of the Colon and Rectum. 2019;62:e325. | Ineligible study design: single group |
| Chen GD, Rizk DEE, Richter HE. Surgical repair of vesico-vaginal fistula: the need for an evidence-based approach. International Urogynecology Journal. 2019;30(2):169-70. | Ineligible publication type: commentary |
| Chen XB, Wang YX, Jiang H, Liao DX, Yu JH, Luo CH. Salvage irrigation-suction in gracilis muscle repair of complex rectovaginal and rectourethral fistulas. World Journal of Gastroenterology. 2013;19(39):6625-9. | Ineligible study design: single arm, no subgrouping or comparison |
| ChiCTR. Comparison of effects of vaginal irrigation with different irrigative solutions before vesicovaginal fistula repair: a prospective, randomised, controlled clinical trial. Trial ID: Chi-CTR-IOR-16008531. 2016. [Accessed 16 February 2022]. | Ineligible intervention: effects of preoperative irrigation, not surgery itself |
| Chibber PJ, Shah HN, Jain P. Laparoscopic O'Conor's repair for vesico-vaginal and vesico-uterine fistulae. BJU International. 2005;96(1):183-6. | Ineligible study design: single group |
| Chigbu CO, Nwogu-Ikojo EE, Onah HE, Iloabachie GC. Juxtacervical vesicovaginal fistulae: outcome by route of repair. Journal of Obstetrics & Gynaecology. 2006;26(8):795-7. | Ineligible intervention: only described as vaginal or abdominal repair with no further specification |
| Chimamise C, Shiripinda I, Munjanja SP, Machinga M. A comparison of quality of life of obstetric fistula survivors in Zimbabwe before and after surgical treatment. African Journal of Urology. 2021;27. | Ineligible study design: single arm, no subgrouping or comparison |
| Chong W, Liu T, Bui A. Incidence and Risk Factors for Postoperative Complications of Rectovaginal Fistula Repairs, Based on Different Surgical Routes. Female Pelvic Medicine & Reconstructive Surgery. 2021;27(1):e82-e90. | Ineligible population: fistula not described as obstetric |
| Clinical Trials Registry - India (CTRI). Single procedure brachytherapy in cervical cancer. Trial ID: CTRI/2017/03/008172. 2017. [Accessed 16 February 2022]. | Ineligible population: women with cervical cancer |
| Clinical Trials Registry - India (CTRI). Study of Durvalumab or Placebo given along with Chemoradiation therapy in women with Locally Advanced Cervical Cancer. Trial ID: CTRI/2019/04/018479. 2019. [Accessed 16 February 2022]. | Ineligible population: women with locally advanced cervical cancer |
| Clinical Trials Registry - India (CTRI). Design & evaluation of new devices for radiation dose delivery in brachytherapy. Trial ID: CTRI/2020/03/023701. 2020. [Accessed 16 February 2022]. | Ineligible population: carcinoma of the uterine or cervix, not obstetric fistula |
| Clinical Trials Registry - India (CTRI). A clinical trial to study the effects of two drugs, Dinoprostone gel and tablet Misoprostol in pregnant women who are planned for induction of labour. Trial ID: CTRI/2020/04/024809. 2020. [Accessed 16 February 2022]. | Ineligible population: pregnant women seeking induction of labour |
| ClinicalTrials.gov. Phase II Clinical Trial, Multicentre, Randomised and Comparative, to Evaluate the Efficacy and Safety of a New Therapy With Autologous Stem Cells Derived From Lipoaspirates for the Non-Surgical Treatment of Complex Perianal Fistula. Trial ID: NCT00115466. 2005. [Accessed 16 February 2022]. | Ineligible population: male and female participants with perianal fistula |
| ClinicalTrials.gov. A Randomized, Prospective, Multi-Centered Study Comparing Clinical Outcomes of the Ligation of Intersphincteric Fistula Tract (LIFT)Procedure Versus Use of Anal Fistula Plug (AFP)in the Surgical Repair of Trans-Sphincteric Anal Fistulae of Cryptoglandular Origin. Trial ID: NCT00830661. 2009. [Accessed 16 February 2022]. | Ineligible population: male and female participants with anal fistula |
| ClinicalTrials.gov. Randomized, Single-blind, Placebo Controlled Multicenter Phase III Study to Assess the Efficacy and Safety of Expanded Autologous Adipose-derived Stem Cells (ASCs) (CX-401), for Treatment of Complex Perianal Fistulas in Perianal Crohn's Disease. Trial ID: NCT01378390. 2011. [Accessed 16 February 2022]. | Ineligible population: participants with Crohn's disease |
| ClinicalTrials.gov. Cancer of the Uterus and Treatment of Stress Urinary Incontinence. Trial ID: NCT02667431. 2016. [Accessed 16 February 2022]. | Ineligible population: cancer and stress urinary incontinence, not obstetric fistula |
| ClinicalTrials.gov. A Phase I, Pilot Trial to Evaluate the Safety and Efficacy of Injection of Allogeneic Mesenchymal Bone-Marrow Derived Human Stem Cells in Patients With Fistulizing Crohn's Disease. Trial ID: NCT02677350. 2016. [Accessed 16 February 2022]. | Ineligible study design: single group assignment |
| ClinicalTrials.gov. Does Extending Catheterization Improve Outcomes in Early Vesicovaginal Fistula Repair Failures? A Prospective Randomized Controlled Trial. Trial ID: NCT03029130. 2017. [Accessed 16 February 2022]. | Ineligible population: not described as obstetric |
| ClinicalTrials.gov. Timing of Repeat Voiding Trials After Outpatient Pelvic Floor Surgery. Trial ID: NCT03048682. 2017. [Accessed 16 February 2022]. | Ineligible population: women had previously undergone surgery for pelvic organ prolapse or stress urinary incontinence; not stated to be related to obstetric fistula |
| ClinicalTrials.gov. The Safety and Efficiacy of Local MSC Injection in the Treatment of Fistulas in Patients With Perianal Crohn's Disease. Trial ID: NCT03056664. 2017. [Accessed 16 February 2022]. | Ineligible population: participants with Crohn's disease |
| ClinicalTrials.gov. Post-market, Prospective, Randomised, Sham-controlled Clinical Trial Designed to Confirm the Efficacy and Safety of the Fotona Smooth Device to Treat Female Stress Urinary Incontinence. Trial ID: NCT03098992. 2017. [Accessed 16 February 2022]. | Ineligible population: women with stress urinary incontinence, not obstetric fistula |
| ClinicalTrials.gov. A New MR-based Perianal Crohn's Disease Activity Score: A Multi-centre Study. Trial ID: NCT03325582. 2017. [Accessed 16 February 2022]. | Ineligible study design: does not appear to be more than one study group |
| ClinicalTrials.gov. A Pilot Study to Assess the Efficacy of an Anorectal Fistula Plug With Sealing of the Internal Opening (Curaseal AF) as a Treatment for Perianal Fistula. Trial ID: NCT03381365. 2017. [Accessed 16 February 2022]. | Ineligible study design: single group assignment |
| ClinicalTrials.gov. Feasibility of the Menstrual Cup for Non-Surgical Management of VVF Among Women Seeking Care at a Health Facility in Ghana. Trial ID: NCT03414060. 2018. [Accessed 16 February 2022]. | Ineligible study design: single group assignment |
| ClinicalTrials.gov. Randomized Trial of Retropubic Versus Single-incision Mid-Urethral Sling (Altis ) for Concomitant Management of Stress Urinary Incontinence During Native Tissue Vaginal Repair. Trial ID: NCT03520114. 2018. [Accessed 16 February 2022]. | Ineligible population: women with pelvic organ prolapse or stress urinary incontinence, not obstetric fistula |
| ClinicalTrials.gov. Mechanism of Action and Clinical Effect of BI 655130 in Patients With Fistulizing Crohn's Disease. Trial ID: NCT03752970. 2018. [Accessed 16 February 2022]. | Ineligible population: participants with Crohn's disease |
| ClinicalTrials.gov. Use of Permacol Paste in Perianal Crohn's Disease. Trial ID: NCT03776825. 2018. [Accessed 16 February 2022]. | Ineligible population: participants with Crohn's disease |
| ClinicalTrials.gov. Comparative Effectiveness of Biofeedback and Injectable Bulking Agents for Treatment of Fecal Incontinence: The Fecal Incontinence Treatment (FIT) Study. Trial ID: NCT03811821. 2019. [Accessed 16 February 2022]. | Ineligible population: men and women with faecal incontinence |
| ClinicalTrials.gov. Surgical Evaluation Of Using The Inner Surface Of The Prepuce In Feminizing Genitoplasty In Cases Of Verilized Females With Congenital Adrenal Hyperplasia. Trial ID: NCT03897504. 2019. [Accessed 16 February 2022]. | Ineligible study design: single group assignment |
| ClinicalTrials.gov. Randomised, Sham Controlled Trial of Fotona Smooth Erbium Yag Laser In the Treatment of Pelvic Organ Prolapse. Trial ID: NCT03995797. 2019. [Accessed 16 February 2022]. | Ineligible population: women with pelvic organ prolapse, not obstetric fistula |
| ClinicalTrials.gov. Randomised, Sham Controlled Trial of Fotona Smooth Erbium Yag Laser In the Treatment of Stress Urinary Incontinence. Trial ID: NCT03996070. 2019. [Accessed 16 February 2022]. | Ineligible population: women with stress urinary incontinence, not OF |
| ClinicalTrials.gov. Use of the Leva Pelvic Digital Health System in Women With Fecal Incontinence: a Pilot Study. Trial ID: NCT04027335. 2019. [Accessed 16 February 2022]. | Ineligible population: women with faecal incontinence; cause of faecal incontinence not defined as obstetric fistula |
| ClinicalTrials.gov. LASER and Radiofrequency as Alternative Treatments for Genitourinary Syndrome of Menopause. Trial ID: NCT04045379. 2019. [Accessed 16 February 2022]. | Ineligible population: genitourinary symptoms of menopause, not obstetric fistula |
| ClinicalTrials.gov. LASER and Radiofrequency as Alternative Treatment of Vaginal Vulvar Atrophy in Women Treated for Breast Cancer. Trial ID: NCT04081805. 2019. [Accessed 16 February 2022]. | Ineligible population: vulvovaginal atrophy in women with breast cancer |
| ClinicalTrials.gov. An Open-Label, Single-Center, Investigator Initiated Phase 1B Trial of E-CEL UVEC as an Adjunct Cell Therapy for Treatment of Anal Fistulas. Trial ID: NCT04190862. 2019. [Accessed 16 February 2022]. | Ineligible study design: single group assignment |
| ClinicalTrials.gov. Impact of Placement of a Diazepam Suppository Intraoperatively on Early Postoperative Pain Following Pelvic Reconstructive Surgery: a Double-Blind, Randomized Placebo-Controlled Trial. Trial ID: NCT04198233. 2019. [Accessed 16 February 2022]. | Ineligible population: women with hysterectomy, not obstetric fistula |
| ClinicalTrials.gov. Fecal Microbial Transplantation (FMT) For the Treatment of Fecal Incontinence in Women. Trial ID: NCT04201821. 2019. [Accessed 16 February 2022]. | Ineligible population: women with faecal incontinence; cause of faecal incontinence not defined as obstetric fistula |
| ClinicalTrials.gov. USTekinumab in Fistulising Perianal Crohn's Disease (CD): The USTAP CD Study. Trial ID: NCT04496063. 2020. [Accessed 16 February 2022]. | Ineligible population: participants with Crohn's disease |
| ClinicalTrials.gov. A Phase IB/IIA Study of Adult Allogeneic Bone Marrow Derived Mesenchymal Stem Cells for the Treatment of Rectovaginal Fistulas in the Setting of Crohn's Disease. Trial ID: NCT04519697. 2020. [Accessed 16 February 2022]. | ineligible population: Crohn's disease patients, not obstetric fistula |
| ClinicalTrials.gov. SASS: Randomized Trial of Single-incision Versus Retropubic Mid-Urethral Sling (Solyx) for Concomitant Management of Stress Urinary Incontinence During Minimally Invasive Sacrocolpopexy. Trial ID: NCT04586166. 2020. [Accessed 16 February 2022]. | Ineligible population: women with pelvic organ prolapse or stress urinary incontinence, not obstetric fistula |
| ClinicalTrials.gov. Barbed Suture Versus Non-Barbed Suture for Posterior Colporrhaphy: A Randomized Controlled Trial. Trial ID: NCT04658784. 2020. [Accessed 16 February 2022]. | Ineligible population: women with pelvic organ prolapse, not obstetric fistula |
| ClinicalTrials.gov. Treatment of Fistula in Ano With Autologous Fatty Tissue Cells Transplantation. Trial ID: NCT04660903. 2020. [Accessed 16 February 2022]. | Ineligible population: fistula in ano, not obstetric fistula |
| ClinicalTrials.gov. To Investigate the Efficacy and Safety of Nimotuzumab Combined With Concurrent Chemoradiotherapy Versus Concurrent Chemoradiotherapy in the Treatment of Local Advanced Cervical Squamous Cell Carcinoma. Trial ID: NCT04678791. 2020. [Accessed 16 February 2022]. | Ineligible population: participants with cervical squamous cell carcinoma, not obstetric fistula |
| ClinicalTrials.gov. Comprehensive Reintegration Assistance for Women With Obstetric Fistula. Trial ID: NCT04748653. 2021. [Accessed 16 February 2022]. | Ineligible study design: single group assignment |
| ClinicalTrials.gov. Stelara and CDED Diet Trial for Crohn's Disease. Trial ID: NCT04779762. 2021. [Accessed 16 February 2022]. | Ineligible population: participants with Crohn's disease |
| ClinicalTrials.gov. Adipose Tissue Injection for the Treatment of Complex Cryptoglandular Perianal Fistula. Trial ID: NCT04790123. 2021. [Accessed 16 February 2022]. | Ineligible population: participants with cryptoglandular fistula, not obstetric fistula |
| ClinicalTrials.gov. Effect of Preoperative Fiber on Postoperative Bowel Function. Trial ID: NCT04882995. 2021. [Accessed 16 February 2022]. | Ineligible population: women with pelvic organ prolapse, not obstetric fistula |
| ClinicalTrials.gov. SPOTLITE: Prospective Observational Study on the Clinical Outcomes of Surgical Interventions in Complex Fistulizing Conditions (CPF-CD, CD-RVF, CCF). Trial ID: NCT04940611. 2021. [Accessed 16 February 2022]. | Ineligible population: participants have Crohn's disease, crypotglandular fistula or other fistulising medical conditions, not obstetric fistula |
| ClinicalTrials.gov. A Perspective, Multi-center, Single Arm Study of Nimotuzumab Combined With IMRT in Elder Patients With Cervical Squamous Cell Carcinoma. Trial ID: NCT04976478. 2021. [Accessed 16 February 2022]. | Ineligible population: participants with squamous cell carcinoma, not obstetric fistula |
| ClinicalTrials.gov. Rectovaginal Fistula in Post-Radiotherapy Advanced Cervical Cancer Patients. Trial ID: NCT05059119. 2021. [Accessed 16 February 2022]. | Ineligible population: women with cervical cancer |
| ClinicalTrials.gov. Musset's Surgical Technique: Evaluation of Long-term Results (LONGOMUSSET). Trial ID: NCT05079139. 2021. [Accessed 16 February 2022]. | Ineligible study design: single arm study |
| ClinicalTrials.gov. Research About the Method of Cotton Padding Promoting the Healing of the Wound After Rectovaginal Fistula Surgery. Trial ID: NCT05138965. 2021. [Accessed 16 February 2022]. | Ineligible study design: single arm study |
| ClinicalTrials.gov. A Phase I Dose Escalation and Cohort Expansion Study to Evaluate the Safety, Tolerance and Pharmacokinetic of BAT1308 Injection in Patients With Advanced Solid Tumors. Trial ID: NCT05155722. 2021. [Accessed 16 February 2022]. | Ineligible study design: single group assignment |
| ClinicalTrials.gov. Comparing Postoperative Pain After LigaSureTM Vessel Sealing Device Versus Conventional Suturing Methods for Vaginal Hysterectomy and Pelvic Reconstructive Surgery: a Randomized Controlled Trial. Trial ID: NCT05192954. 2022. [Accessed 16 February 2022]. | Ineligible population: women after hysterectomy, not obstetric fistula |
| **Autologous Slings With Vesico-Vaginal Fistula Repair** | Ineligible population: fistula not described as obstetric |
| Colenbrander J, Heesakkers J, Martens F. Vesico-Vaginal Fistula Repair by a Vaginal Approach. Urologia Internationalis. 2021;105(11):1113-8. | Ineligible population: less than 80% obstetric |
| Companywala RT, Bhattacharya M, Purandare VN. Clinical evaluation of genito-urinary fistula. Journal of Postgraduate Medicine. 1978;24(2):109-12. | Ineligible study design: single group |
| Creanga AA, Genadry RR. Obstetric fistulas: a clinical review. International Journal of Gynecology & Obstetrics. 2007;99:S40‐6. | Ineligible study design: literature review |
| Cromwell D, Hilton P. Retrospective cohort study on patterns of care and outcomes of surgical treatment for lower urinary-genital tract fistula among English National Health Service hospitals between 2000 and 2009. BJU International. 2013;111(4):E257-62. | Ineligible population: less than 80% obstetric |
| D'Ambrosio G, Paganini AM, Guerrieri M, Barchetti L, Lezoche G, Fabiani B, et al. Minimally invasive treatment of rectovaginal fistula. Surgical Endoscopy. 2012;26(2):546-50. | Ineligible study design: single arm, no subgrouping or comparison |
| Dafnis G. Transsphincteric repair of rectourethral fistulas: 15 years of experience with the York Mason approach. International Journal of Urology. 2018;25(3):290-6. | Ineligible population: participants were men |
| Debela TF, Hordofa ZA, Aregawi AB, Sori DA. Quality of life of obstetrics fistula patients before and after surgical repair in the Jimma University Medical Center, Southwest Ethiopia. BMC Women's Health. 2021;21(1):212. | Ineligible study design: single arm, no subgrouping or comparison |
| Delamou A, Delvaux T, Beavogui AH, Leveque A, Zhang WH, De Brouwere V. A descriptive longitudinal study protocol: recurrence and pregnancy post-repair of obstetric fistula in Guinea. BMC Pregnancy & Childbirth. 2016;16(1):299. | Ineligible intervention: does not specify type of repair |
| Delamou A, Delvaux T, Utz B, Camara BS, Beavogui AH, Cole B, et al. Factors associated with loss to follow-up in women undergoing repair for obstetric fistula in Guinea. Tropical Medicine & International Health. 2015;20(11):1454-61. | Ineligible intervention: does not specify type of repair |
| Delamou A, Delvaux T, Beavogui AH, Toure A, Kolie D, Sidibe S, et al. Factors associated with the failure of obstetric fistula repair in Guinea: implications for practice. Reproductive Health. 2016;13(1):135. | Ineligible intervention: type of surgery not specified |
| Delamou A, Delvaux T, El Ayadi AM, Tripathi V, Camara BS, Beavogui AH, et al. Fistula recurrence, pregnancy, and childbirth following successful closure of female genital fistula in Guinea: a longitudinal study. The Lancet Global Health. 2017;5(11):e1152-60. | Ineligible study design: single arm, no subgrouping or comparison |
| Delamou A, Diallo M, Beavogui AH, Delvaux T, Millimono S, Kourouma M, et al. Good clinical outcomes from a 7-year holistic programme of fistula repair in Guinea. Tropical Medicine & International Health. 2015;20(6):813-9. | Ineligible intervention: does not specify type of repair |
| Derso EA, Ayalew S, Eshete A, Wale M. Determinants of time to recovery from obstetric fistula by using the data of university of Gondar teaching hospital fistula center, Gondar -Ethiopia: A parametric survival regression analysis. Cogent Medicine. 2020;7(1):1816259. | Ineligible intervention: does not specify type of repair |
| Devaraju S, Nerli R, Reddy M, Guntaka A, Patil S. Functional outcome following laparoscopic VVF repair in comparison with vaginal repair (Abstract number 382). In: (ICS) ICS, editor. Proceedings of the 41st Annual Meeting of the International Continence Society (ICS); August 29 2011 to September 2 2011; Glasgow, Scotland. | Ineligible population: iatrogenic fistula, not obstetric fistula |
| Dolan LM, Dixon WE, Hilton P. Urinary symptoms and quality of life in women following urogenital fistula repair: a long-term follow-up study. BJOG: An International Journal of Obstetrics & Gynaecology. 2008;115(12):1570-4. | Ineligible population: less than 80% obstetric |
| Egziabher TG, Eugene N, Ben K, Fredrick K. Obstetric fistula management and predictors of successful closure among women attending a public tertiary hospital in Rwanda: a retrospective review of records. BMC Research Notes. 2015;8:774. | Ineligible intervention: type of surgery not specified |
| Ekwedigwe KC, Isikhuemen ME, Sunday-Adeoye I, Yakubu EN, Eliboh MO. Bladder spasm following urogenital fistula repair. International Journal of Gynaecology & Obstetrics. 2017;138(3):299-303. | Ineligible intervention: repair described as vaginal or abdominal with no further description |
| El Ayadi A, Barageine J, Korn A, Obore S, Byamugisha J, Mwanje H, et al. Predictors of continence following surgical repair of vesicovaginal fistula in Uganda. International Journal of Gynecology and Obstetrics. 2018;143:419-20. | Ineligible population: fistula not described as obstetric |
| El Ayadi A, Nalubwama H, Barageine J, Obore S, Kakaire O, Mwanje H, et al. Sociodemographic, clinical and service predictors of postsurgical reintegration score among Ugandan women treated for obstetric fistula. Tropical Medicine and International Health. 2017;22:333. | Ineligible intervention: does not specify type of repair |
| El Ayadi AM, Barageine J, Korn A, Kakaire O, Turan J, Obore S, et al. Trajectories of women's physical and psychosocial health following obstetric fistula repair in Uganda: a longitudinal study. Tropical Medicine & International Health. 2019;24(1):53-64. | Ineligible population: less than 80% obstetric |
| El Ayadi AM, Nalubwama H, Barageine JK, Miller S, Obore S, Kakaire O, et al. Feasibility and acceptability of mobile phone data collection for longitudinal follow-up among patients treated for obstetric fistula in Uganda. Health Care for Women International. 2020:1-15. | Ineligible study design: about feasibility of data collection, not interventional |
| El Ayadi AM, Painter CE, Delamou A, Barr-Walker J, Korn A, Obore S, et al. Rehabilitation and reintegration programming adjunct to female genital fistula surgery: A systematic scoping review. International Journal of Gynecology & Obstetrics. 2020;148:42-58. | Ineligible study design: systematic review that contains both eligible and ineligible study designs |
| El-Azab AS, Abolella HA, Farouk M. Update on vesicovaginal fistula: A systematic review. Arab Journal of Urology Print. 2019;17(1):61-8. | Ineligible population: number of studies described as obstetric or iatrogenic not reported; not possible to tell if meets eligibility criteria as a result |
| El Imam M, El Hassan M, Adam I. Vesicovaginal fistula in Sudanese women. Saudi Medical Journal. 2005;26(2):341-2. | Ineligible population: less than 80% obstetric |
| El-Tabey NA, Ali-El-Dein B, Shaaban AA, El-Kappany HA, Mokhtar AA, El-Azab M, et al. Urological trauma after gynecological and obstetric surgeries. Scandinavian Journal of Urology & Nephrology. 2006;40(3):225-31. | Ineligible population: less than 80% obstetric |
| Ellis CN. Outcomes after repair of rectovaginal fistulas using bioprosthetics. Diseases of the Colon & Rectum. 2008;51(7):1084-8. | Ineligible population: fistula not described as obstetric |
| Emembolu J. The obstetric fistula: factors associated with improved pregnancy outcome after a successful repair. International Journal of Gynecology & Obstetrics. 1992;39(3):205-12. | Ineligible intervention: does not specify type of repair |
| Epiu I, Alia G, Mukisa J, Tavrow P, Lamorde M, Kuznik A. Estimating the cost and cost-effectiveness for obstetric fistula repair in low-income countries. Value in Health. 2018;21:S126. | Ineligible study design: economic evaluation |
| Espinoza AR, Cardozo A, Rojas C, Petit M, Lopez V. Vaginal repair of vesico-vaginal fistula: Experience at a tertiary care center in Venezuela [Spanish]. Archivos Espanoles de Urologia. 2019;72(4):398-405. | Ineligible population: less than 80% obstetric |
| Etabbal AM, El Kawafi RM, Gero AH. Vesicovaginal fistula diagnosis and repair, Benghazi, Libya (nine years of of experience). Libyan International Medical University Journal. 2017;1(2):93-105. | Ineligible population: less than 80% obstetric |
| EU Clinical Trials Register. A Phase III multicenter, single blind, randomized, comparative, add-on clinical trial, in three parallel groups, to evaluate the efficacy and safety of a new therapy with adipose-derived autologous stem cells for the treatment of complex perianal fistulas in patients without inflammatory bowel disease' FATT I: Fistula Advanced Therapy Trial (I),FATT 1. Trial ID: 2006-003370-95/DE. 2007. [Accessed 16 February 2022]. | Ineligible population: men and women with perianal fistula, not obstetric fistula |
| EU Clinical Trials Register. A Phase III multicenter, single blind, randomized, comparative, add-on clinical trial, in three parallel groups, to evaluate the efficacy and safety of a new therapy with adipose-derived autologous stem cells for the treatment of complex perianal fistulas in patients without inflammatory bowel disease' FATT I: Fistula Advanced Therapy Trial (I),FATT 1. Trial ID: 2006-003370-95/NL. 2007. [Accessed 16 February 2022]. | Ineligible population: men and women with perianal fistula, not obstetric fistula |
| EU Clinical Trials Register. A Phase 2, 12 Week, Multicenter, Randomized, Double-Blind, Placebo-Controlled, Parallel Group, Proof Of Concept Study Evaluating the Efficacy and Safety of PD 0299685 for the Treatment of Symptoms Associated with Interstitial Cystitis/Painful Bladder Syndrome. Trial ID: 2008-002421-37/FR. 2008. [Accessed 16 February 2022]. | Ineligible population: participants with interstitial cystitis |
| EU Clinical Trials Register. Randomized, single-blind, placebo controlled multicenter phase III study to assess the efficacy and safety of expanded autologous adipose-derived stem cells (eASCs) (CX-401), for treatment of complex perianal fistulas in perianal Crohn s disease. FATT II: Fistula Advanced Therapy Trial (II),FATT II. Trial ID: 2008-004286-25/NL. 2008. [Accessed 16 February 2022]. | Ineligible population: participants with Crohn's disease |
| EU Clinical Trials Register. A Phase 2B, Randomized, Double Blind, Placebo-Controlled, Dose Ranging Study Evaluating the Efficacy and Safety of Tanezumab for the Treatment of Moderate to Severe Pain Associated with Interstitial Cystitis/ Painful Bladder Syndrome (IC/PBS). Trial ID: 2009-014597-17/FI. 2009. [Accessed 16 February 2022]. | Ineligible population: participants with interstitial cystitis |
| EU Clinical Trials Register. A Phase 2B, Randomized, Double Blind, Placebo-Controlled, Dose Ranging Study Evaluating the Efficacy and Safety of Tanezumab for the Treatment of Moderate to Severe Pain Associated with Interstitial Cystitis/ Painful Bladder Syndrome (IC/PBS). Trial ID: 2009-014597-17/SE. 2009. [Accessed 16 February 2022]. | Ineligible population: participants with interstitial cystitis |
| EU Clinical Trials Register. A Phase 2B, Randomized, Double Blind, Placebo-Controlled, Dose Ranging Study Evaluating the Efficacy and Safety of Tanezumab for the Treatment of Moderate to Severe Pain Associated with Interstitial Cystitis/ Painful Bladder Syndrome (IC/PBS). Trial ID: 2009-014597-17/SK. 2010. [Accessed 16 February 2022]. | Ineligible population: participants with interstitial cystitis |
| EU Clinical Trials Register. Phase II, open-label clinical trial to evaluate the safety and efficacy of platelet-rich plasma and fibrin clot processed with PRGF-System tecnology in the treatment of anal fistulas in Crohn's patients Ensayo cl nico fase II, abierto para evaluar la seguridad y factibilidad del plasma rico plaquetas y coagulo de fibrina procesados con la tecnologia PRGF-System, en el tratamiento de f stulas anales de enfermos con Crohn. Trial ID: 2011-004820-37/ES. 2012. [Accessed 16 February 2022]. | Ineligible population: participants with Crohn's disease |
| EU Clinical Trials Register. A phase III, randomised, double blind, parallel group, placebo controlled, multicentre study to assess efficacy and safety of expanded allogeneic adipose-derived stem cells (eASCs) for the treatment of perianal fistulising Crohn s disease over a period of 24 weeks and an extended follow-up period up to 52 weeks. ADMIRE-CD study.,ADMIRE-CD study. Trial ID: 2011-006064-43/NL. 2012. [Accessed 16 February 2022]. | Ineligible population: participants with Crohn's disease |
| EU Clinical Trials Register. Phase II, open-label clinical trial to evaluate the safety and efficacy of platelet-rich plasma and fibrin clot in processed with PRGF-system technology, in the treatment of anal fistulas in Crohn's patients with no concomitant therapy with second-line drugs. Ensayo cl nico fase II, abierto para evaluar la seguridad y factibilidad del plasma rico plaquetas y coagulo de fibrina procesados con la tecnolog a PRGF-System, en el tratamiento de f stulas anales de enfermos con Crohn, sin terapia concomitante con f rmacos de segunda l nea. Trial ID: 2011-006127-38/ES. 2012. [Accessed 16 February 2022]. | Ineligible population: participants with Crohn's disease |
| EU Clinical Trials Register. Multimodal treatment of perianal fistulas in Crohn's disease: seton vs anti-TNF vs advancement plasty,PISA-trial. Trial ID: 2013-002932-25/NL. 2013. [Accessed 16 February 2022]. | Ineligible population: participants with Crohn's disease |
| EU Clinical Trials Register. A Phase 2, Double-Blind, Randomized, Placebo-Controlled Study Evaluating the Efficacy and Safety of Filgotinib in the Treatment of Perianal Fistulizing Crohn s Disease Estudio en fase II, doble ciego, aleatorizado y controlado con placebo para evaluar la eficacia y la seguridad de filgotinib en el tratamiento de la enfermedad de Crohn perianal fistulizante. Trial ID: 2016-003153-15/ES. 2017. [Accessed 16 February 2022]. | Ineligible population: participants with Crohn's disease |
| EU Clinical Trials Register. A Phase 2, Double-Blind, Randomized, Placebo-Controlled Study Evaluating the Efficacy and Safety of Filgotinib in the Treatment of Perianal Fistulizing Crohn s Disease II. f zis , kett s vak, randomiz lt, placebo kontroll lt vizsg lat a filgotinib hat soss g nak s biztons goss g nak rt kel s re, a perianalis fistul val j r Crohn-betegs g kezel s ben. Trial ID: 2016-003153-15/HU. 2017. [Accessed 16 February 2022]. | Ineligible population: participants with Crohn's disease |
| EU Clinical Trials Register. A Phase 2, Double-Blind, Randomized, Placebo-Controlled Study Evaluating the Efficacy and Safety of Filgotinib in the Treatment of Perianal Fistulizing Crohn’s Disease. Trial ID: 2016-003153-15/PL. 2017. [Accessed 16 February 2022]. | Ineligible population: participants with Crohn's disease |
| EU Clinical Trials Register. Phase III Clinical Trial, Mulchintric, Randomized, Blind Double in Two Parallel Groups to Compare the Effectiveness and Safety of the Simple Closure of the Cryptoglandular Anal Fistula With or Without the Adjuvant Administration of Plasma Rico in Growth Factors (Endoret / PRGF Technology), in a Period of 48 Weeks. Trial ID: 2017-001209-34/ES. 2017. [Accessed 16 February 2022]. | Ineligible population: participants with cryptoglandular fistula, not obstetric fistula |
| EU Clinical Trials Register. Mechanism of Action and Clinical Effect of BI 655130 in Patients with fistulizing Crohn s Disease,BI 655130 treatment of fistulizing CD. Trial ID: 2017-003090-34/AT. 2018. [Accessed 16 February 2022]. | Ineligible population: participants with Crohn's disease |
| EU Clinical Trials Register. Mechanism of Action and Clinical Effect of BI 655130 in Patients with fistulizing Crohn s Disease,BI 655130 treatment of fistulizing CD. Trial ID: 2017-003090-34/BE. 2018. [Accessed 16 February 2022]. | Ineligible population: participants with Crohn's disease |
| EU Clinical Trials Register. Mechanism of Action and Clinical Effect of BI 655130 in Patients with fistulizing Crohn’s Disease,BI 655130 treatment of fistulizing CD. Trial ID: 2017-003090-34/DE. 2018. [Accessed 16 February 2022]. | Ineligible population: participants with Crohn's disease |
| EU Clinical Trials Register. Mechanism of Action and Clinical Effect of BI 655130 in Patients with fistulizing Crohn’s Disease,BI 655130 treatment of fistulizing CD. Trial ID: 2017-003090-34/NL. 2018. [Accessed 16 February 2022]. | Ineligible population: participants with Crohn's disease |
| EU Clinical Trials Register. Mechanism of Action and Clinical Effect of BI 655130 in Patients with fistulizing Crohn s Disease Klinikai vizsg lat a BI 655130 hat sm dj nak vizsg lat ra sipollyal j r Crohn- betegs gben szenved betegekben,BI 655130 treatment of fistulizing CD. Trial ID: 2017-003090-34/HU. 2019. [Accessed 16 February 2022]. | Ineligible population: participants with Crohn's disease |
| EU Clinical Trials Register. Treatment of Perianal FIstulas in Crohn’s Disease: Surgical closure vs Anti-TNF,PISA II-trial. Trial ID: 2018-002064-15/NL. 2019. [Accessed 16 February 2022]. | Ineligible population: participants with Crohn's disease |
| EU Clinical Trials Register. Pilot Project for the Treatment of Perianal Disease through the Local Injection of Remsima Guided by Ultrasound. Number of cases. Proyecto Piloto del Tratamiento de la Enfermedad Perianal Mediante la Inyecci n Local de Remsima Guiada por Ecograf a. Serie de Casos. Trial ID: 2019-000668-27/ES. 2019. [Accessed 16 February 2022]. | Ineligible population: participants with Crohn's disease |
| Fan X, Ma X, Lai Y, Li Z, Huang J, Huang H. Suprapubic Transvesical Repair of Vesicovaginal Fistula Using a Homemade Laparoscopic Single-Port Device: Experience of 42 Patients. Frontiers in Surgery. 2021;8:744226. | Ineligible study design: cross-sectional |
| Fang G, Hong L, Li B, Liu C, Wu D, Hong S, et al. Transvaginal genital fistula repair with insertion of Foley catheter via fistula tract. Journal of Obstetrics & Gynaecology Research. 2015;41(7):1049-55. | Ineligible study design: single arm, no subgrouping or comparison |
| Farahat YA, Elbendary MA, Elgamal OM, Tawfik AM, Bastawisy MG, Radwan MH, et al. Application of small intestinal submucosa graft for repair of complicated vesicovaginal fistula: a pilot study. Journal of Urology. 2012;188(3):861-4. | Ineligible population: less than 80% of the women had fistula of obstetric aetiology |
| Fazari AB, Elmusharaf K, Mukhtar WA, AbdAlla M, St. Louis S, Najim S. Surgical outcomes of obstetric fistula repair, West Darfur experience/Sudan. Female Pelvic Medicine and Reconstructive Surgery. 2012;1:S35. | Ineligible intervention: does not specify type of repair |
| Frajzyngier V, Ruminjo J, Barone MA. Authors' response to: Factors influencing choice of surgical route of repair of genitourinary fistula, and the influence of route of repair on surgical outcomes: findings from a prospective cohort study. BJOG: An International Journal of Obstetrics & Gynaecology. 2013;120(11):1441-2. | Ineligible publication type: letter with no further information |
| Frajzyngier V, Li G, Larson E, Ruminjo J, Barone MA. Development and comparison of prognostic scoring systems for surgical closure of genitourinary fistula. American Journal of Obstetrics & Gynecology. 2013;208(2):112.e1-11. | Ineligible population: fistula not described as obstetric |
| Frajzyngier V, Ruminjo J, Asiimwe F, Barry TH, Bello A, Danladi D, et al. Factors influencing choice of surgical route of repair of genitourinary fistula, and the influence of route of repair on surgical outcomes: findings from a prospective cohort study. BJOG: An International Journal of Obstetrics & Gynaecology. 2012;119(11):1344-53. | Ineligible intervention: surgical intervention only described as vaginal or abdominal, no other details |
| Frajzyngier V, Ruminjo J, Barone MA. Factors influencing urinary fistula repair outcomes in developing countries: a systematic review. American Journal of Obstetrics & Gynecology. 2012;207(4):248-58. | Ineligible population: cases of obstetric fistula in included studies less than 80% |
| Fu J, Liang Z, Zhu Y, Cui L, Chen W. Surgical repair of rectovaginal fistulas: predictors of fistula closure. International Urogynecology Journal. 2019;30(10):1659-65. | Ineligible population: less than 80% of the women had fistula of obstetric aetiology |
| Fu W, Yi S, An M, Tang Y, Tang L, Wang Y, et al. Effect of diverting stoma for rectovaginal fistula: A protocol of systematic review and meta-analysis. Medicine. 2020;99(49):e23202. | Ineligible intervention: stoma not an eligible intervention |
| Ganda S, Diagne BA, Gueye S, Harrisou A, Soumana A. Correlation of surgical repair outcomes of female genital fistulae and their classification. International Journal of Gynecology and Obstetrics. 2015;5:E600. | Ineligible population: fistula not described as obstetric |
| Ganesh PR, Mernoff R, Dikkers R, Nundwe W, Pope R. A Comparison of Postoperative Surgical Outcomes among Women Undergoing Obstetric Fistula Repair with and without HIV. International Journal of MCH & AIDS. 2021;10(2):191-7. | Ineligible intervention: does not specify type of repair |
| García Guido L. Fístulas urogenitales. In: Castillo P, A. E, Malfatto GL, Pons JE, editors. Uroginecología y Disfunciones del Piso Pélvico. Montevideo: Oficina del Libro FEFMUR; 2007. p. 217-34. | Ineligible publication type: book |
| Garduno-Segovia N, Bravo-Lopez GM, Mucientes-Avellaneda VM, Rico-Fontana E, Reyes-Gutierrez MA, Soria-Fernandez GR. Result comparison of the abdominal and vaginal approaches in vesicovaginal fistula management [Spanish]. Revista Mexicana de Urologia. 2019;79(2):e03. | Ineligible study design: cross-sectional |
| Garofalo TE, Delaney CP, Jones SM, Remzi FH, Fazio VW. Rectal advancement flap repair of rectourethral fistula: a 20-year experience. Diseases of the Colon & Rectum. 2003;46(6):762-9. | Ineligible population: iatrogenic fistula, not obstetric fistula |
| Gedik A, Deliktas H, Celik N, Kayan D, Bircan MK. Is percutaneous cystostomy always necessary in transvaginal repair of benign vesicovaginal fistulae? International Journal of Clinical and Experimental Medicine. 2016;9(5):8755-9. | Ineligible population: less than 80% of the women had fistula of obstetric aetiology |
| Gedik A, Deliktas H, Celik N, Kayan D, Bircan MK. Which Surgical Technique Should be Preferred to Repair Benign, Primary Vesicovaginal Fistulas? Urology Journal. 2015;12(6):2422-7. | Ineligible population: less than 80% of the women had fistula of obstetric aetiology |
| Geidam AD, Barka E. The Management of Vesico-vaginal Fistula in State Specialist Hospital Maiduguri, Borno State. British Journal of Medicine & Medical Research. 2016;17(1):1-6. | Ineligible intervention: does not specify type of repair |
| Genadry R. Clinical and quality of life outcomes after fistula surgery - a multi-country study. International Journal of Gynecology and Obstetrics. 2009;2:S32. | Ineligible intervention: does not specify type of repair |
| Gessessew A, Mesfin M. Genitourinary and rectovaginal fistulae in Adigrat Zonal Hospital, Tigray, north Ethiopia. Ethiopian Medical Journal. 2003;41(2):123-30. | Ineligible study design: single group |
| Goh JT, Browning A, Berhan B, Chang A. Predicting the risk of failure of closure of obstetric fistula and residual urinary incontinence using a classification system. International Urogynecology Journal. 2008;19(12):1659-62. | Ineligible intervention: type of surgical procedure not described |
| Goh JT, Krause H, Tessema AB, Abraha G. Urinary symptoms and urodynamics following obstetric genitourinary fistula repair. International Urogynecology Journal. 2013;24(6):947-51. | Ineligible population: secondary repair of urinary incontinence, not obstetric fistula |
| Gottgens KW, Smeets RR, Stassen LP, Beets G, Breukink SO. The disappointing quality of published studies on operative techniques for rectovaginal fistulas: a blueprint for a prospective multi-institutional study. Diseases of the Colon & Rectum. 2014;57(7):888-98. | Ineligible population: most studies examined Crohn’s or cryptoglandular fistula |
| Group AMMS. Burden of severe maternal morbidity and association with adverse birth outcomes in sub-Saharan Africa and south Asia: protocol for a prospective cohort study. Journal of Global Health. 2016;6(2):020601. | Ineligible study design: study of prevalence, not interventional |
| Grewal M, Pakzad M, Hamid R, Ockrim J, Greenwell TJ. The medium- to long-term functional outcomes of women who have had successful anatomical closure of vesicovaginal fistulae. Urology Annals. 2019;11:247-51. | Ineligible population: less than 80% of the women had fistula of obstetric aetiology |
| Grott M, Rickert A, Hetjens S, Kienle P. Clinical outcome and quality of life after gracilis muscle transposition for fistula closure over a 10-year period. International Journal of Colorectal Disease. 2021;36(3):569-80. | Ineligible population: less than 80% of the women had fistula of obstetric aetiology |
| Gumodoka B, Mach E, Majinge CR. Genito-urinary fistula patients at Bugando Medical Centre. East African Medical Journal. 2010;87(7):294-8. | Ineligible population: fistulae not described as being of obstetric aetiology |
| Guntaka AK, Nerli RB, Reddy MN, Hiremath MB. Transvesicoscopic repair of vesicovaginal fistula: short term follow-up. Indian Journal of Urology. 2014;30:S75‐6. | Ineligible population: fistula not described as obstetric |
| Gupta R, Mahajan A, Mahajan M, Anand A, Masood S. Management of Genitourinary Fistulas Following Benign Gynecological and Obstetric Procedures: A Single Surgeon Experience. Journal of Mid-Life Health. 2020;11(3):156-60. | Ineligible population: less than 80% of the women had fistula of obstetric aetiology |
| Gupta SK, Pandey A, Kumar P, Srivastava S, Singh S, Rawat J. Evaluation and management of rectovaginal fistula in anorectal malformation: an observational study. Pediatric Surgery International. 2021;37(11):1601-6. | Ineligible study design: single arm, no subgrouping or comparison |
| Gurlek A, Gherardini G, Coban YK, Gorgu M, Erdogan B, Evans GR. The repair of multiple rectovaginal fistulas with the neurovascular pudendal thigh flap (Singapore flap). Plastic & Reconstructive Surgery. 1997;99(7):2071-3. | Ineligible study design: case report |
| Hauch A, McKee RM, Li WY, Crowley JS, Ramamoorthy S, Dobke M. Rectovaginal Fistula Repair 1 Year Later: Lessons Learned. Annals of Plastic Surgery. 2021;87(2):187-93. | Ineligible study design: single group |
| Hawkins L, Spitzer RF, Christoffersen-Deb A, Leah J, Mabeya H. Characteristics and surgical success of patients presenting for repair of obstetric fistula in western Kenya. International Journal of Gynaecology & Obstetrics. 2013;120(2):178-82. | Ineligible intervention: type of surgical procedure not described |
| Higashino T, Sakuraba M, Fukunaga Y, Oshima A, Masuda H, Ito M. Surgical outcome for colorectal or urinary tract-related fistula: Usefulness of vascularized tissue transfer-a retrospective study. Journal of Plastic, Reconstructive and Aesthetic Surgery. 2021;74:1041-9. | Ineligible population: less than 80% of the women had fistula of obstetric aetiology |
| Hillary CJ, Osman NI, Hilton P, Chapple CR. The Aetiology, Treatment, and Outcome of Urogenital Fistulae Managed in Well- and Low-resourced Countries: A Systematic Review. European Urology. 2016;70(3):478-92. | Ineligible population: compares high and low income countries; population not representative of overall PICO |
| Hillary CJ, Chapple CR. The choice of surgical approach in the treatment of vesico-vaginal fistulae. Asian Journal of Urology. 2018;5(3):155-9. | Ineligible study design: literature review, not systematic review |
| Hilton P, Ward A. Epidemiological and surgical aspects of urogenital fistulae: a review of 25 years' experience in southeast Nigeria. International Urogynecology Journal. 1998;9(4):189-94. | Ineligible intervention: surgical intervention only described as vaginal or abdominal, no other details |
| Holt LM, Potluri T, Tanner JP, Duffy S, Wasingya L, Greene KA. 63: Risk factors for failed surgical repair of urogenital fistulas. American Journal of Obstetrics and Gynecology. 2020;222:S813. | Ineligible intervention: surgical intervention only described as vaginal or abdominal, no other details |
| Holt L, Potluri T, Tanner JP, Duffy S, Wasingya L, Greene K. Risk factors for failed surgical repair of urogenital fistulas. International Urogynecology Journal. 2020;31:S71-2. | Ineligible publication type: duplicate |
| Holt L, Potluri T, Tanner JP, Duffy S, Wasingya L, Greene K. Risk factors for early and late failures following repair of urogenital fistulas. International Urogynecology Journal. 2021;32(9):2473-82. | Ineligible intervention: surgical intervention only described as vaginal or abdominal, no other details |
| Holzer E, Schmidt T, Trotnow S. [Proceedings: Late results of vaginal surgery for vesicovaginal fistula occlusion]. Archiv fur Gynakologie. 1975;219(1):251-2. | Ineligible study design: case report |
| Hotouras A, Ribas Y, Zakeri S, Murphy J, Bhan C, Chan CL. Gracilis muscle interposition for rectovaginal and anovaginal fistula repair: a systematic literature review. Colorectal Disease. 2015;17(2):104-10. | Ineligible population: less than 80% of the women had fistula of obstetric aetiology |
| Hull TL, El-Gazzaz G, Gurland B, Church J, Zutshi M. Surgeons should not hesitate to perform episioproctotomy for rectovaginal fistula secondary to cryptoglandular or obstetrical origin. Diseases of the Colon & Rectum. 2011;54(1):54-9. | Ineligible population: less than 80% of the women had fistula of obstetric aetiology |
| Ijaiya MA, Rahman AG, Aboyeji AP, Olatinwo AWO, Esuga SA, Ogah OK, et al. Vesicovaginal Fistula: a Review of Nigerian Experience. West African Journal of Medicine. 2010;29(5):293-8. | Ineligible study design: literature review, not systematic review |
| Inipavudu B, Mitterschiffthaler G, Hasibeder WR, Dunser MW. Spinal versus epidural anesthesia for vesicovaginal fistula repair surgery in a rural sub-Saharan African setting. Journal of Clinical Anesthesia. 2007;19(6):444-7. | Ineligible population: fistula not described as obstetric |
| Islam T, Akhter S. Identification of risk factors of failed closure of genitourinary fistulae and residual incontinence. International Journal of Gynecology and Obstetrics. 2018;143:417. | Ineligible intervention: type of surgical procedure not described |
| Itam S, Barratt R, Pakzad M, Hamid R, Ockrim J, Shah J, et al. Increasing the rate of vaginal repair of vesicovaginal fistulae does not affect outcome. Neurourology and Urodynamics. 2018;37:S600-1. | Ineligible intervention: surgical intervention only described as vaginal or abdominal, no other details |
| Itam S, Barratt R, Pakzad M, Hamid R, Ockrim J, Shah J, et al. Increasing vaginal repair of vesicovaginal fistulae does not affect outcome. Journal of Clinical Urology. 2018;11:86-7. | Ineligible population: fistula not described as obstetric |
| Jadhav S, Raut A, Mandke J, Patil S, Vora Kittur D. Nonclosure of rectourethral fistula during posterior sagittal anorectoplasty: our experience. Journal of Indian Association of Pediatric Surgeons. 2013;18(1):5‐6. | Ineligible population: anorectal malformation |
| Jatoi N, Jatoi NM, Shaikh F, Sirichand P. Key to successful vesico-vaginal fistula repair--an experience of urogenital fistula surgeries and outcome at gynaecological surgical camp-2005. Journal of Ayub Medical College, Abbottabad: JAMC. 2008;20(2):125-7. | Ineligible study design: single arm, no subgrouping or comparison |
| Javed A, Abdullah A, Faruqui N, Syed SS, Binat ul M, Pirzada AJ. Doctor! Will I be dry? Factors determining recurrence after vesicovaginal fistula repair. JPMA - Journal of the Pakistan Medical Association. 2015;65(9):954-9. | Ineligible population: fistulas not 80% obstetric aetiology; although total obstetric reasons and caesarean equal more than 80%, it is not reported whether the caesareans led to ‘high’ or ‘low’ fistula |
| Jeppson PC, Nosti PA, Mishra K, Uwamahoro A, Hampton BS. The effect of verbal education on postoperative knowledge for women presenting for vesicovaginal fistula surgery in Kigali, Rwanda. Female Pelvic Medicine and Reconstructive Surgery. 2013;1:S26-7. | Ineligible intervention: education and testing post-surgical knowledge |
| Ju M, Wang X, Xia C, Ju Y. Tissue selecting technique for adult women with low rectovaginal fistula. Journal of the Chinese Medical Association: JCMA. 2022;85:120-3. | Ineligible population: less than 80% of the women had fistula of obstetric aetiology |
| Junaid R. Profile of women who experienced vesicovaginal fistula due to obstetric trauma. Pakistan Journal of Medical Sciences. 2010;26(2):499. | Ineligible study design: cross-sectional |
| Kafetsioulis A, Swinney J. Urinary diversion by ileal conduit. A long-term follow-up. British Journal of Urology. 1968;40(1):1-11. | Ineligible population: less than 80% of the women had fistula of obstetric aetiology |
| Kapoor R, Sureka SK, Jena R. Vesico-vaginal fistula repair by transvaginal route: Comparison of resource utilization and postoperative outcome with literature reported population minimally invasive approach cohort. Journal of Urology. 2019;201:e886. | Ineligible intervention: surgical intervention only described as vaginal or abdominal, no other details |
| Karakus SC, User IR, Akcaer V, Ceylan H, Ozokutan BH. Posterior sagittal anorectoplasty in vestibular fistula: with or without colostomy. Pediatric Surgery International. 2017;33(7):755-9. | Ineligible population: anorectal malformation in infants |
| Karp NE, Berger MB, Low C, DeLancey JO, Fenner DE. Comparison in outcomes between obstetric and non-obstetric rectovaginal fistula repairs. Female Pelvic Medicine and Reconstructive Surgery. 2016;22:S56. | Ineligible population: less than 80% of the women had fistula of obstetric aetiology |
| Karp NE, Kobernik EK, Berger MB, Low CM, Fenner DE. Do the Surgical Outcomes of Rectovaginal Fistula Repairs Differ for Obstetric and Nonobstetric Fistulas? A Retrospective Cohort Study. Female Pelvic Medicine & Reconstructive Surgery. 2019;25(1):36-40. | Ineligible population: less than 80% of the women had fistula of obstetric aetiology |
| Kayondo M, Mukasa P, Senkugu J, Tarnay C, Hartshorn T, Margolis MT, et al. Predictors for obstetric fistulae repair outcomes at Mbarara regional referral hospital in Southwestern Uganda. Female Pelvic Medicine and Reconstructive Surgery. 2011;1:S175. | Ineligible intervention: type of surgical procedure not described |
| Kayondo M, Wasswa S, Kabakyenga J, Mukiibi N, Senkungu J, Stenson A, et al. Predictors and outcome of surgical repair of obstetric fistula at a regional referral hospital, Mbarara, western Uganda. BMC Urology. 2011;11:23. | Ineligible intervention: type of surgical procedure not described |
| Kelly J, Kwast BE. Epidemiologic study of vesicovaginal fistulas in Ethiopia. International Urogynecology Journal. 1993;4:278-81. | Ineligible study design: single arm, no subgrouping or comparison; epidemiological |
| Kelly J. Ethiopia: An epidemiological study of vesico-vaginal fistula in Addis Ababa. World Health Statistics Quarterly. 1995;48:15-7. | Ineligible study design: single group |
| Kersting S, Athanasiadis CJ, Jung KP, Berg E. Operative results, sexual function and quality of life after gracilis muscle transposition in complex rectovaginal fistulas. Colorectal Disease. 2019;21(12):1429-37. | Ineligible population: not obstetric fistula |
| Keyser L, McKinney J, Salmon C, Furaha C, Kinsindja R, Benfield N. Analysis of a pilot program to implement physical therapy for women with gynecologic fistula in the Democratic Republic of Congo. International Journal of Gynecology & Obstetrics. 2014;127(2):127-31. | Ineligible study design: mixed-methods study |
| Keyser L, McKinney J, Hosterman L, Chen CCG. Rehabilitative care practices in the management of childbirth-related pelvic fistula: A systematic review. International Urogynecology Journal. 2021;32(9):2311-24. | Ineligible study design: mixed-methods systematic review |
| Khalid N, Qureshi F. Vesicovaginal fistula: psychosocial problems in rural areas of Pakistan. Journal of Bahria University Medical and Dental College. 2018;8(2):65-6. | Ineligible study design: editorial |
| Khan AL, Khan M, Bano A. Vesicovaginal fistula; surgical management. The Professional Medical Journal. 2004;11(3):261-6. | Ineligible population: one group consisted of participants who were classed as having “high” VVF; not an eligible type of fistula |
| Khanduja KS, Padmanabhan A, Kerner BA, Wise WE, Aguilar PS. Reconstruction of rectovaginal fistula with sphincter disruption by combining rectal mucosal advancement flap and anal sphincteroplasty. Diseases of the Colon & Rectum. 1999;42(11):1432-7. | Ineligible study design: single group |
| Khanduja KS, Yamashita HJ, Wise WE, Jr., Aguilar PS, Hartmann RF. Delayed repair of obstetric injuries of the anorectum and vagina. A stratified surgical approach. Diseases of the Colon & Rectum. 1994;37(4):344-9. | Ineligible population: less than 80% of the women had fistula of obstetric aetiology |
| Khisa W, Wakasiaka S, Lavender T. The correlates of a successful fistula surgery in Africa, a prospective cohort. BJOG: An International Journal of Obstetrics and Gynaecology. 2021;128:268. | Ineligible intervention: type of surgical procedure not described |
| Kim M, Park N, Yun H. Factors affecting surgical treatment and outcomes of vesico-vaginal fistula: A retrospective study. Asian Journal of Surgery. 2021;44(1):427-8. | Ineligible population: less than 80% of the women had fistula of obstetric aetiology |
| Kopp DM, Bengtson AM, Tang JH, Chipungu E, Moyo M, Wilkinson J. Authors' reply re: Use of a postoperative pad test to identify continence status in women after obstetric vesicovaginal fistula repair: a prospective cohort study. BJOG: An International Journal of Obstetrics & Gynaecology. 2017;124(10):1623. | Ineligible publication type: letter with no additional information |
| Kopp DM, Tang JH, Bengtson AM, Chi BH, Chipungu E, Moyo M, et al. Continence, quality of life and depression following surgical repair of obstetric vesicovaginal fistula: a cohort study. BJOG: An International Journal of Obstetrics & Gynaecology. 2019;126(7):926-34. | Ineligible intervention: type of surgical procedure not described |
| Kopp DM, Wilkinson J, Bengtson A, Chipungu E, Pope RJ, Moyo M, et al. Fertility outcomes following obstetric fistula repair: a prospective cohort study. Reproductive Health. 2017;14(1):159. | Ineligible study design: single arm, no subgrouping or comparison |
| Kopp DM, Bengtson AM, Tang JH, Chipungu E, Moyo M, Wilkinson J. Use of a postoperative pad test to identify continence status in women after obstetric vesicovaginal fistula repair: a prospective cohort study. BJOG: An International Journal of Obstetrics & Gynaecology. 2017;124(6):966-72. | Ineligible study design: focuses on validation of pad test as an outcome measure, not interventional |
| Krause HG, Wong V, Goh J. Does obstructed labour resulting in obstetric fistula cause levator muscle avulsion? Female Pelvic Medicine and Reconstructive Surgery. 2014;1:S205. | Ineligible study design: not interventional, assesses epidemiology and aetiology of levator muscle avulsion |
| Krause HG, Hall BA, Ng SK, Natukunda H, Singasi I, Goh JTW. Mental health screening in women with severe pelvic organ prolapse, chronic fourth-degree obstetric tear and genital tract fistula in western Uganda. International Urogynecology Journal. 2017;28(6):893-7. | Ineligible population: less than 80% of the women had fistula of obstetric aetiology |
| Lambertz A, Luken B, Ulmer TF, Bohm G, Neumann UP, Klink CD, et al. Influence of diversion stoma on surgical outcome and recurrence rates in patients with rectovaginal fistula - A retrospective cohort study. International Journal Of Surgery. 2016;25:114-7. | Ineligible population: less than 80% of the women had fistula of obstetric aetiology |
| Landry E, Frajzyngier V, Ruminjo J, Asiimwe F, Barry TH, Bello A, et al. Profiles and experiences of women undergoing genital fistula repair: Findings from five countries. Global Public Health. 2013;8:926-42. | Ineligible intervention: type of surgical procedure not described |
| Lassey AT, Peterson CF, Ampofo K. Pregnancy and delivery after ureterosigmoidotomy for vesicovaginal fistula. International Journal of Gynaecology & Obstetrics. 2002;79(1):25-6. | Ineligible study design: case report |
| Lee D, Dillon BE, Lemack GE, Zimmern PE. Long-term functional outcomes following nonradiated vesicovaginal repair. Journal of Urology. 2014;191(1):120-4. | Ineligible population: less than 80% of the women had fistula of obstetric aetiology |
| Lee D, Zimmern PE. Long-term functional outcomes following non-radiated urethrovaginal fistula repair. World Journal of Urology. 2016;34(2):291-6. | Ineligible population: fistula not described as obstetric |
| Lembrande RO, Vadra JE. Fístula rectovaginal. La Prensa Médica Argentina. 1985;72(15):514-9. | Ineligible study design: commentary |
| Lengmang S, Shephard S, Datta A, Lozo S, Kirschner CV. Pubovesical sling for residual incontinence after successful vesicovaginal fistula closure: a new approach to an old procedure. International Urogynecology Journal. 2018;29(10):1551-6. | Ineligible population: secondary repair of urinary incontinence, not obstetric fistula |
| Levitt MA, King SK, Bischoff A, Alam S, Gonzalez G, Pena A. The Gonzalez hernia revisited: use of the ischiorectal fat pad to aid in the repair of rectovaginal and rectourethral fistulae. Journal of Pediatric Surgery. 2014;49(8):1308-10. | Ineligible study design: single group |
| Lewis R, Lunniss PJ, Hammond TM. Novel biological strategies in the management of anal fistula. Colorectal Disease. 2012;14(12):1445-55. | Ineligible population: less than 80% of the women had fistula of obstetric aetiology |
| Li S, Liu Y, Chang X, Li K, Yang D, Zhang X, et al. Two-Staged Versus Three-Staged Laparoscopic Anorectoplasty for Patients with Rectoprostatic and Bladder Neck Fistulas: A Comparative Study. Journal of Laparoendoscopic & Advanced Surgical Techniques Part A. 2019;29(11):1486-91. | Ineligible population: anorectal malformation in infants |
| Linder BJ, Raju R, Tappy EE, Bews KA, Habermann EB, Occhino JA. Perioperative outcomes of rectovaginal fistula repair based on surgical approach. International Urogynecology Journal. 2019;30:S280-1. | Ineligible population: fistula not described as obstetric |
| Lindsey I, Smilgin-Humphreys MM, Cunningham C, Mortensen NJ, George BD. A randomized, controlled trial of fibrin glue vs. conventional treatment for anal fistula. Diseases of the Colon and Rectum. 2002;45(12):1608‐15. | Ineligible population: participants had Crohn’s disease or fistula arising due to cryptoglandular causes |
| Lo TS, Chua S, Wijaya T, Kao CC, Uy-Patrimonio MC. Clinical relevance and treatment outcomes of vesicovaginal fistula (VVF) after obstetric and gynecologic surgery. Taiwanese Journal of Obstetrics & Gynecology. 2019;58(1):111-6. | Ineligible population: less than 80% of the women had fistula of obstetric aetiology |
| Lo TS, Huang YH, Dass AK, Karim N, Uy-Patrimonio MC. Rectovaginal fistula: Twenty years of rectovaginal repair. Journal of Obstetrics & Gynaecology Research. 2016;42(10):1361-8. | Ineligible population: less than 80% of the women had fistula of obstetric aetiology |
| Loposso M, Hakim L, Ndundu J, Lufuma S, Punga A, De Ridder D. Predictors of Recurrence and Successful Treatment Following Obstetric Fistula Surgery. Urology. 2016;97:80-5. | Ineligible study design: single group |
| Lotocki W, Jozwik M, Jozwik M. Prognosis of fertility after surgical closure of vesicouterine fistula. European Journal of Obstetrics, Gynecology, & Reproductive Biology. 1996;64(1):87-90. | Ineligible study design: single group |
| Luo DY, Shen H. Transvaginal Repair of Apical Vesicovaginal Fistula: A Modified Latzko Technique-Outcomes at a High-volume Referral Center. European Urology. 2019;76(1):84-8. | Ineligible study design: cross-sectional |
| Mafo Degge H, Hayter M, Laurenson M. An integrative review on women living with obstetric fistula and after treatment experiences. Journal of Clinical Nursing. 2017;26(11):1445-57. | Ineligible study design: mixed-methods review |
| Mahfouz W, Abdel-Karim AM. Laparoscopic VVF repair: Current trends. Current Women's Health Reviews. 2013;9(2):93-8. | Ineligible study design: literature review, not systematic review |
| Mancini M, Righetto M, Modonutti D, Morlacco A, Dal Moro F, Zattoni F. Successful Treatment of Vesicovaginal Fistulas via an Abdominal Transvesical Approach: A Single-center 50-yr Experience. European Urology Focus. 2021;7(6):1485-92. | Ineligible population: less than 80% of the women had fistula of obstetric aetiology |
| Margulies SL, Geller EJ. Vaginal Repair of Vesicovaginal Fistula: Comparison of National Practice Patterns by Surgeon Specialty. Journal of the American College of Surgeons. 2021;233(5):S164. | Ineligible population: fistula not described as obstetric |
| Maroyi R, Keyser L, Hosterman L, Notia A, Mukwege D. The mobile surgical outreach program for management of patients with genital fistula in the Democratic Republic of Congo. International Journal of Gynaecology & Obstetrics. 2020;148:27-32. | Ineligible population: less than 80% of the women had fistula of obstetric aetiology |
| Maroyi R, Shahid U, Vangaveti V, Rane A, Mukwege D. Obstetric vesico-vaginal fistulas: Midvaginal and juxtacervical fistula repair outcomes in the Democratic Republic of Congo. International Journal of Gynaecology & Obstetrics. 2021;153(2):294-9. | Ineligible intervention: describes layers and suturing in surgical procedure but not the specific type of surgery undertaken |
| Martinez Carrancio LA, Eguiluz Jardin EJ. Fístulas Vesicovaginales. In: Castillo P, Edgardo A, editors. Tratado de Perineología: Disfunciones del Piso Pélvico. Montevideo: Academia Nacional de Medicina; 2019. p. 173-80. | Ineligible publication type: book chapter |
| Massengill JC, Baker TM, Von Pechmann WS, Horbach NS, Hurtado EA. Commonalities of cerclage-related genitourinary fistulas. Female Pelvic Medicine & Reconstructive Surgery. 2012;18(6):362-5. | Ineligible study design: case series |
| Matei DV, Zanagnolo V, Vartolomei MD, Crisan N, Ferro M, Bocciolone L, et al. Robot-Assisted Vesico-Vaginal Fistula Repair: Our Technique and Review of the Literature. Urologia Internationalis. 2017;99(2):137-42. | Ineligible study design: case series |
| Matsuo K, Rosenshein NB, Im DD. The Big Seep. American Journal of Obstetrics & Gynecology. 2008;198(1):145.e1-2. | Ineligible study design: case report |
| Maulet N, Keita M, Macq J. Medico-social pathways of obstetric fistula patients in Mali and Niger: an 18-month cohort follow-up. Tropical Medicine & International Health. 2013;18(5):524-33. | Ineligible intervention: type of surgical procedure not described |
| Melah GS, El-Nafaty AU, Bukar M. Early versus late closure of vesicovaginal fistulas. International Journal of Gynecology & Obstetrics. 2006;93(3):252-3. | Ineligible intervention: early versus late surgical repair but no further details |
| McCauley M, van den Broek N. Improving the physical, psychological and social ill-health of women affected by obstetric fistula. BJOG: An International Journal of Obstetrics & Gynaecology. 2019;126(7):935. | Ineligible publication type: commentary |
| Mernoff R, Chigwale S, Pope R. Physical etiology of sexual dysfunction in obstetric fistula patients: A prospective study. International Journal of Gynecology & Obstetrics. 2020;149(2):178-83. | Ineligible study design: single group, pre-post |
| Michel F, Gaillet S, Boissier R, Delaporte V, Lechevallier E, Bensadoun H, et al. Epidemiology and care pathway of vesicovaginal fistulas managed in France between 2010 and 2018. World Journal of Urology. 2022;20:20. | Ineligible population: less than 80% of the women had fistula of obstetric aetiology |
| Miklos JR, Moore RD, Chinthakanan O. Laparoscopic and Robotic-assisted Vesicovaginal Fistula Repair: A Systematic Review of the Literature. Journal of Minimally Invasive Gynecology. 2015;22(5):727-36. | Ineligible population: less than 80% of the women had fistula of obstetric aetiology |
| Miklos JR, Moore RD. Laparoscopic extravesical vesicovaginal fistula repair: our technique and 15-year experience. International Urogynecology Journal. 2015;26(3):441-6. | Ineligible study design: single arm, no subgrouping or comparison |
| Mohr S, Brandner S, Mueller MD, Dreher EF, Kuhn A. Sexual function after vaginal and abdominal fistula repair. American Journal of Obstetrics & Gynecology. 2014;211(1):74.e1-6. | Ineligible population: less than 80% of the women had fistula of obstetric aetiology |
| Morhason-Bello IO, Ojengbede OA, Adedokun BO, Okunlola MA, Oladokun A. Uncomplicated midvaginal vesico-vaginal fistula repair in ibadan: a comparison of the abdominal and vaginal routes. Annals of Ibadan Postgraduate Medicine. 2008;6(2):39-43. | Ineligible intervention: surgical intervention only described as vaginal or abdominal, no other details |
| Mubeen RM, Naheed F, Anwar F. Management of vesicovaginal fistulae in urological context. Journal of the College of Physicians and Surgeons--Pakistan: JCPSP. 2007;17(1):28-31. | Ineligible study design: case series |
| Mubeen RM, Naheed F, Ashraf R, Malik AA. Surgical management of simple vesicovaginal fistulae. Annals of King Edward Medical University. 2005;11(1):27-9. | Ineligible intervention: surgical intervention only described as vaginal or abdominal, no other details |
| Mukwege D, Peters L, Amisi C, Mukwege A, Smith AR, Miller JM. Panzi score as a parsimonious indicator of urogenital fistula severity derived from Goh and Waaldijk classifications. International Journal of Gynecology & Obstetrics. 2018;142(2):187-93. | Ineligible study design: prognostic tool |
| Muleta M, Tafesse B, Aytenfisu HG. Antibiotic use in obstetric fistula repair: single blinded randomized clinical trial. Ethiopian Medical Journal. 2010;48(3):211‐7. | Ineligible intervention: study of antibiotics, not eligible intervention |
| Muleta M, Rasmussen S, Kiserud T. Obstetric fistula in 14,928 Ethiopian women. Acta Obstetricia et Gynecologica Scandinavica. 2010;89(7):945-51. | Ineligible intervention: type of surgical procedure not described |
| Murray C, Goh JT, Fynes M, Carey MP. Urinary and faecal incontinence following delayed primary repair of obstetric genital fistula. BJOG: An International Journal of Obstetrics & Gynaecology. 2002;109(7):828-32. | Ineligible study design: case series |
| Murthy NR, Jahagirdar R, Mallikarjun G, Jagadeeshwar T, Chandar GR, Kumar RA. A clinical study of various presentations and different modalities in management of vesico-vaginal fistula. International Archives of Integrated Medicine. 2016;3(9):1-8. | Ineligible population: less than 80% of the women had fistula of obstetric aetiology |
| Nagraj HK, Kishore TA, Nagalaksmi S. Early laparoscopic repair for supratrigonal vesicovaginal fistula. International Urogynecology Journal. 2007;18(7):759-62. | Ineligible study design: single group |
| Nasir JM, Qayyum A, Hidayat U. Comparison of transvesical repair of vesico-vaginal fistula and use of free bladder mucosal graft. Biomedica. 2008;24:23-5. | Ineligible population: less than 80% of the women had fistula of obstetric aetiology |
| Neogi SB, Negandhi H, Bharti P, Zodpey S, Mathur A. Burden and management of obstetric fistula in South-East Asian region countries: A systematic review and meta-analysis. Indian Journal of Public Health. 2020;64(4):386-92. | Ineligible study design: includes mixed study designs, including case reports |
| Nielsen HS, Lindberg L, Nygaard U, Aytenfisu H, Johnston OL, Sorensen B, et al. A community-based long-term follow up of women undergoing obstetric fistula repair in rural Ethiopia. BJOG: An International Journal of Obstetrics & Gynaecology. 2009;116(9):1258-64. | Ineligible study design: single group assignment |
| Ockrim JL, Greenwell TJ, Foley CL, Wood DN, Shah PJ. A tertiary experience of vesico-vaginal and urethro-vaginal fistula repair: factors predicting success. BJU International. 2009;103(8):1122-6. | Ineligible population: less than 80% of the women had fistula of obstetric aetiology |
| Okunola TO, Yakubu E, Daniyan B, Ekwedigwe K, Eliboh M, Sunday-Adeoye I. Profile and outcome of patients with recurrent urogenital fistula in a fistula centre in Nigeria. International Urogynecology Journal. 2019;30(2):197-201. | Ineligible intervention: type of surgical procedure not described |
| Ommer A, Herold A, Berg E, Furst A, Schiedeck T, Sailer M. German S3-Guideline: rectovaginal fistula. German Medical Science. 2012;10:Doc15. | Ineligible study design: guidelines |
| Opare-Addo HS, Danso KA, Turpin CA. Pregnancy after ureterosigmoidostomy for vesicovaginal fistula. International Journal of Gynecology & Obstetrics. 2009;106(1):66. | Ineligible study design: case report |
| Ostad M, Uzzo RG, Coleman J, Young GP. Use of a free bladder mucosal graft for simple repair of vesicovaginal fistulae. Urology. 1998;52(1):123-6. | Ineligible study design: case series |
| Otubu JA, Kumi GO, Ezem BU. Pregnancy and delivery after successful repair of vesicovaginal fistula. International Journal of Gynecology & Obstetrics. 1982;20(2):163-6. | Ineligible intervention: type of surgical procedure not described |
| Ouedraogo I, Payne C, Nardos R, Adelman AJ, Wall LL. Obstetric fistula in Niger: 6-month postoperative follow-up of 384 patients from the Danja Fistula Center. International Urogynecology Journal. 2018;29(3):345-51. | Ineligible intervention: type of surgical procedure not described |
| Pactr. An RCT of early discharge with a catheter Vs late discharge after catheter removal following urogenital fistula repair among women in Mulago Hospital. 2013. Trial ID: PACTR201306000556261. [Accessed 16 February 2022]. | Ineligible population: fistula not described as obstetric |
| Palma PCR, Riccetto CLZ, Rodrigues Netto Junior N. Autocistoplastia no tratamento das fistulas vesicovaginais complexas. Jornal Brasileiro de Urologia. 1993;19(4):265-7. | Ineligible study design: describes an operative technique |
| Panaiyadiyan S, Nayyar BU, Nayyar R, Kumar N, Seth A, Kumar R, et al. Impact of vesicovaginal fistula repair on urinary and sexual function: patient-reported outcomes over long-term follow-up. International Urogynecology Journal. 2021;32(9):2521-8. | Ineligible study design: cross-sectional |
| Pandey S. Re: Quality of life among women in Bangladesh following ileal conduit urinary diversion operations for irreparable vesicovaginal fistula and bladder exstrophy; observational study. BJOG: An International Journal of Obstetrics & Gynaecology. 2017;124(12):1908-9. | Ineligible publication type: letter |
| Paprottka FJ, Krezdorn N, Lohmeyer JA, Young K, Kuhbier J, Keck M, et al. Plastic reconstructive surgery techniques using VRAM or gracilis flaps in order to successfully treat complex urogenital fistulas. Journal of Plastic, Reconstructive & Aesthetic Surgery: JPRAS. 2016;69(1):128-37. | Ineligible population: includes both male and female participants |
| Park SO, Hong KY, Park KJ, Chang H, Shin JY, Jeong SY. Treatment of rectovaginal fistula with gracilis muscle flap transposition: long-term follow-up. International Journal of Colorectal Disease. 2017;32(7):1029-32. | Ineligible population: less than 80% of the women had fistula of obstetric aetiology |
| Parkar RB, Pinder LF, Wanyoike JG, Patel Y, Otieno D, Palkhi Y, et al. Laparoscopic surgery in low-income and limited-resource settings: does it safely add value? A review of 2,901 laparoscopic gynecologic procedures. World Journal of Laparoscopic Surgery. 2016;9(2):82‐5. | Ineligible population: not focused on obstetric fistula |
| Patil S, Goel A, Dalela D, Singh V, Singh B, Kumar M, et al. Voiding dysfunction after vesicovaginal and urethrovaginal fistula repair-does it really occur? A prospective study. Indian Journal of Urology. 2014;1:S127. | Ineligible intervention: surgical intervention only described as vaginal or abdominal, no other details |
| Perveen S. Vesicovaginal fistula; a complication of obstructed labor article citation. The Professional Medical Journal-Quarterly. 2013;20(5):852-4. | Ineligible study report: case report |
| Polk HC, Jr., Welch JP, Nagaraj HS, Amin M. Rectosigmoidal adenocarcinoma after successful pull-through for imperforate anus and rectourethral fistula: a footnote to Dr. Hugh Young's 1933 operation. Johns Hopkins Medical Journal. 1982;151(4):151-4. | Ineligible study design: case report |
| Pope R, Brown RH, Chalamanda C, Hollier LH, Wilkinson JP. The gracilis muscle flap for irreparable, "impossible", and recurrent obstetric fistulas. International Journal of Gynecology and Obstetrics. 2018;143(3):390-2. | Ineligible study design: case series |
| Pope R, Chang O, Stokes M, Ganesh P, Wilkinson J. Extended foley catheterization for women with obstetric fistulas. International Journal of Gynecology and Obstetrics. 2018;143:866. | Ineligible study design: risk factors to successful or unsuccessful extension, not interventional |
| Pope R, Hollier PC, Brown RH, Chalamanda C, Hollier LH, Jr., Wilkinson J. A retrospective review to identify criteria for incorporating the Singapore flap and gracilis muscle flap into obstetric fistula repair. International Journal of Gynecology & Obstetrics. 2020;148:37-41. | Ineligible study design: case series |
| Pope R, Ganesh P, Chalamanda C, Nundwe W, Wilkinson J. Sexual Function Before and After Vesicovaginal Fistula Repair. Journal of Sexual Medicine. 2018;15(8):1125-32. | Ineligible study design: single group, pre-post |
| Pope R, Mernoff R, Chalamanda C, Bengtson AM. Obstetric fistula scoring tool for surgical trainees. International Journal of Gynecology & Obstetrics. 2022;156(3):502-7. | Ineligible intervention: type of surgical procedure not described |
| Pope RJ, Kopp DM, Chipungu E, Chalamanda C, Wilkinson JP. Association of ureteric stent placement/ duration and pyelonephritis in vesicovaginal fistula patients. International Urogynecology Journal and Pelvic Floor Dysfunction. 2016;27(1):S109‐10. | Ineligible intervention: stents, examining prevention of obstruction, not obstetric fistula |
| Potluri T, Holt L, Tanner JP, Wasingya L, Duffy S, Greene K. Evaluating risk factors associated with need for blood transfusions after urogenital fistula repair in Uganda. International Urogynecology Journal. 2020;31:S225. | Ineligible intervention: linked to full Potluri study report; surgical intervention only described as vaginal or abdominal, no other details |
| Potluri T, Holt LM, Tanner JP, Wasingya L, Duffy S, Greene KA. 56: Evaluating risk factors associated with need for blood transfusions after urogenital fistula repair in Uganda. American Journal of Obstetrics and Gynecology. 2020;222(3):S808-9. | Ineligible intervention: linked to full Potluri study report; surgical intervention only described as vaginal or abdominal, no other details |
| Potluri TK, Holt LM, Tanner JP, Wasingya L, Duffy S, Greene KA. Risk factors for perioperative blood transfusions after urogenital fistula repair in Uganda: a retrospective cohort study. BJOG: An International Journal of Obstetrics & Gynaecology. 2022;129(1):120-6. | Ineligible intervention: surgical intervention only described as vaginal or abdominal, no other details |
| Pradhan HK, Dangal G, Karki A, Shrestha R, Bhattachan K, Upadhyay AM, et al. Clinical Profile of Urogenital Fistula in Kathmandu Model Hospital. Journal of Nepal Health Research Council. 2020;18(2):210-3. | Ineligible population: less than 80% of the women had fistula of obstetric aetiology |
| Pshak T, Nikolavsky D, Terlecki R, Flynn BJ. Is tissue interposition always necessary in transvaginal repair of benign, recurrent vesicovaginal fistulae? Urology. 2013;82(3):707-12. | Ineligible population: less than 80% of the women had fistula of obstetric aetiology |
| Raassen TJ, Hancock B. Factors influencing choice of surgical route of repair of genitourinary fistula, and the influence of route of repair on surgical outcomes: findings from a prospective cohort study. BJOG: An International Journal of Obstetrics & Gynaecology. 2013;120(11):1441. | Ineligible publication type: letter |
| Raithel M, Vetter M, Braun A, Vasilakis T, Hagel AF. Meta-analysis and own experience in the treatment of recto-urogenital fistula using the over-the-scope clip (OTSC). United European Gastroenterology Journal. 2017;5:A237. | Ineligible population: fistula not described as obstetric |
| Rajamaheswari N, Chhikara AB, Seethalakshmi K, Bail A, Agarwal S. Trans-vaginal repair of gynecological supratrigonal vesicovaginal fistulae: a worthy option! Urology Annals. 2012;4(3):154-7. | Ineligible population: iatrogenic fistula from gynaecological surgery |
| Raju R, Linder BJ, Bews KA, Tappy E, Habermann EB, Occhino JA. Perioperative Outcomes of Rectovaginal Fistula Repair Based on Surgical Approach: A National Contemporary Analysis. Female Pelvic Medicine & Reconstructive Surgery. 2021;27(2):e342-7. | Ineligible population: fistula not described as obstetric |
| Reisenauer C. Presentation and management of rectovaginal fistulas after delivery. International Urogynecology Journal. 2016;27(6):859-64. | Ineligible study design: case series |
| Reisenauer C. Vesicovaginal fistulas: a gynecological experience in 41 cases at a German pelvic floor center. Archives of Gynecology & Obstetrics. 2015;292(2):245-53. | Ineligible study design: case series |
| Reisenauer C, Amend B, Falch C, Abele H, Brucker SY, Andress J. Evaluation and management of obstetric genital fistulas treated at a pelvic floor centre in Germany. BMC Women's Health. 2021;21(1):1-4. | Ineligible study design: case series |
| Rijo E, Bielsa O, Lorente JA, Gil-Vernet JM, Fumadó L, Francés A, et al. Complex vesico-vaginal fistula repair with posterosuperior bladder flap. International Brazilian Journal of Urology. 2011;37(6):802-3. | Ineligible study design: case report |
| Romanova A, Sifri Y, Menhaji K, Gaigbe-Togbe B, Seaman C, Hardart A, et al. Vesicovaginal fistula repair and surgeon specialty: Analysis of a national database. Female Pelvic Medicine and Reconstructive Surgery. 2021;27:S15. | Ineligible population: fistula not described as obstetric |
| Roush KM. Social implications of obstetric fistula: an integrative review. Journal of Midwifery & Women's Health. 2009;54(2):e21-33. | Ineligible study design: mixed-methods review |
| Ruminjo J, Barone M, Frajzyngier V. Clinical procedures, practices and postoperative outcomes in surgical repair of female genital fistula: A prospective cohort study. Urology. 2012;1:S147. | Ineligible population: fistula not described as obstetric |
| Ruminjo JK, Frajzyngier V, Bashir Abdullahi M, Asiimwe F, Barry TH, Bello A, et al. Clinical procedures and practices used in the perioperative treatment of female genital fistula during a prospective cohort study. BMC Pregnancy & Childbirth. 2014;14(1):220. | Ineligible population: fistula not described as obstetric |
| Sabate Arroyo XA, Pastor Lopez S, Prats De Puig JM. Novel use of indocianine green for the vesicovaginal fistula repair. European Urology Open Science. 2020;19:e2357. | Ineligible study design: case report |
| Sadiq G, Sadiq M, Sultana N. Obstetric trauma is the commonest cause of urogenital fistulae. Rawal Medical Journal. 2008;33:197-200. | Ineligible population: less than 80% of the women had fistula of obstetric aetiology |
| Salom EM, Mendez LE, Schey D, Lambrou N, Kassira N, Gomez-Marin O, et al. Continent ileocolonic urinary reservoir (Miami pouch): The University of Miami experience over 15 years. Women's Oncology Review. 2004;190(4):199-201. | Ineligible population: less than 80% of the women had fistula of obstetric aetiology |
| Sang-Keon LEE, Yong-Seok LEE, Seung-Yong S, Won-Jai LEE, Dong-Won LEE. Double-sided folded internal pudendal artery perforator flap for the repair of a recurrent rectovaginal fistula. Archives of Plastic Surgery. 2018;45(1):90-2. | Ineligible study design: case report |
| Sang-Wook BAI, Sung-Hoon KIM, Han-Sung K, Koon-Ho RHA, Kyung-Ah C, Sei-Kwang KIM, et al. Surgical Outcome of Female Genital Fistula in Korea. Yonsei Medical Journal. 2002;43(3):315-9. | Ineligible population: less than 80% of the women had fistula of obstetric aetiology |
| Sankhwar S. Comparative analysis of laparoscopic versus open abdominal method of supratrigonal VVF repair. International Journal of Gynecology and Obstetrics. 2018;143:864. | Ineligible intervention: surgery described as laparoscopic or open but with no further details of surgery type |
| Schlussel AT, Lustik MB, Delaney CP, Stein SL, Reynolds HL, Senagore AJ, et al. Rectourethral fistulas: A comparison of the National Inpatient Sample and the American College of Surgeons National Surgical Quality Improvement Program. American Journal of Surgery. 2017;213(4):723-30.e4. | Ineligible population: includes both men and women |
| Shamout S, Anderson K, Baverstock R, Carlson K. Evaluation of surgical approaches for vesicovaginal fistulae repair: the case for transvaginal repair as the gold standard. International Urogynecology Journal. 2021;32(9):2429-35. | Ineligible population: less than 80% of the women had fistula of obstetric aetiology |
| Shephard SN, Lengmang SJ, Anzaku SA, Mamven OV, Kirschner CV. Effect of HIV infection on outcomes after surgical repair of genital fistula. International Journal of Gynaecology & Obstetrics. 2017;138(3):293-8. | Ineligible population: fistula not described as obstetric |
| Shephard SN, Lengmang SJ, Kirschner CV. Bladder stones in vesicovaginal fistula: is concurrent repair an option? Experience with 87 patients. International Urogynecology Journal. 2017;28(4):569-74. | Ineligible population: fistula not described as obstetric |
| Shieh CJ, Gennaro AR. Rectovaginal fistula: a review of 11 years experience. International Surgery. 1984;69(1):69-72. | Ineligible population: iatrogenic fistula, not obstetric fistula |
| Shittu OS, Ojengbede OA, Wara LHI. A review of postoperative care for obstetric fistulas in Nigeria. International Journal of Gynecology & Obstetrics. 2007;99:S79-84. | Ineligible study design: literature review, not systematic review |
| Shrestha DB, Budhathoki P, Karki P, Jha P, Mainali G, Dangal G, et al. Vesico-Vaginal Fistula in Females in 2010-2020: a Systemic Review and Meta-analysis. Reproductive Sciences. 2022;3:03. | Ineligible population: less than 80% of the women within the studies had fistula of obstetric aetiology |
| Shrey J, Naveen K, Rohit K, Sanjoy S, Aneesh S, Rakesh K. Vesico-vaginal fistula repair by transvaginal route: Comparison of resource utilisation and outcome with literature reported population matched minimally invasive cohort. Indian Journal of Urology. 2020;36:S51. | Ineligible population: fistula not described as obstetric |
| Singh O, Gupta SS, Hastir A. Urogenital fistulae: a prospective study of 50 cases at a tertiary care hospital, important issues missed. Urology Annals. 2011;3(2):114. | Ineligible publication type: letter |
| Singh RB, Dalal S, Nanda S, Pavithran NM. Management of female uro-genital fistulas: framing certain guidelines. Urology Annals. 2010;2(1):2-6. | Ineligible population: less than 80% of the women within the studies had fistula of obstetric aetiology |
| Singh S, Chandhiok N, Singh Dhillon B. Obstetric fistula in India: current scenario. International Urogynecology Journal. 2009;20(12):1403-5. | Ineligible study design: not an interventional study |
| Singh V, Jhanwar A, Mehrotra S, Paul S, Sinha RJ. A comparison of quality of life before and after successful repair of genitourinary fistula: Is there improvement across all the domains of WHOQOL-BREF questionnaire? African Journal of Urology. 2015;21(4):230-4. | Ineligible population: not possible to tell numbers of women with obstetric fistula compared to those with iatrogenic fistula |
| Singh V, Sinha R, Bansal A, Mehrotra S, Singh K. Prospective randomized comparison of repairing simple vesicovaginal fistula with or without interposition flap: a tertiary care hospital study from nothern India. Journal of Urology. 2017;197(4):e529. | Ineligible population: not possible to tell numbers of women with obstetric fistula compared to those with iatrogenic fistula |
| Singh V, Sinha R, Bansal A, Purkait B, Mehrotra S. Prospective randomized comparison of repairing simple vesicovaginal fistula with or without interposition flap. European Urology Supplements. 2018;17(2):e1771. | Ineligible population: not possible to tell numbers of women with obstetric fistula compared to those with iatrogenic fistula |
| Singh W, Sethi RS, Lal A. Repair of vesicovaginal fistula. Transperitoneal approach with bisection of bladder. Journal of the Indian Medical Association. 1966;47(12):609-11. | Ineligible study design: case report |
| Sjoveian S, Vangen S, Mukwege D, Onsrud M. Surgical outcome of obstetric fistula: a retrospective analysis of 595 patients. Acta Obstetricia et Gynecologica Scandinavica. 2011;90(7):753-60. | Ineligible study design: cross-sectional |
| Sombie I, Kambou T, Conombo SG, Sankara O, Ouedraogo L, Zoungrana T, et al. [Retrospective study of urogenital fistula in Burkina Faso from 2001 to 2003]. Medecine Tropicale. 2007;67(1):48-52. | Ineligible study design: cross-sectional |
| Stokes M, Wilkinson J, Ganesh P, Nundwe W, Pope R. Persistent depression after obstetric fistula repair. International Journal of Gynecology and Obstetrics. 2018;143:418-9. | Ineligible intervention: type of surgical procedure not described |
| Stokes MJ, Wilkinson JP, Ganesh P, Nundwe W, Pope RJ. Persistent depression after obstetric fistula repair. International Journal of Gynecology & Obstetrics. 2019;147(2):206-11. | Ineligible intervention: type of surgical procedure not described |
| Studniarek A, Borsuk D, Kim D, Tremblay J, Al-Khamis A, Kochar K, et al. Rectal advancement flap (RAF) and interpositional biological mesh as the method of choice for low rectovaginal fistulas. Diseases of the Colon & Rectum. 2019;62(6):e270. | Ineligible population: less than 80% of the women had fistula of obstetric aetiology |
| Studniarek A, Abcarian A, Pan J, Wang H, Gantt G, Jr., Abcarian H. What is the best method of rectovaginal fistula repair? A 25-year single-center experience. Techniques in Coloproctology. 2021;25(9):1037-44. | Ineligible population: less than 80% of the women had fistula of obstetric aetiology |
| Sturiale A, Fabiani B, Menconi C, Giani I, Cafaro D, Toniolo G, et al. Autologous, micro-fragmented and minimally manipulated adipose tissue as an innovative approach for the treatment of complex anal fistulas: A prospective observational study. Techniques in Coloproctology. 2018;22:407. | Ineligible population: includes men and women who had undergone proctologic surgery |
| Sunday-Adeoye I, Uraku AJ, Uraku OH, Lengmang SJ, Chima GA. Asssessment of some selected vitamin levels in patients with vesico - vaginal fistula in Jos, Northern Nigeria. International Journal of Medical and Surgical Sciences (Print). 2017;4(3):1209-15. | Ineligible study design: not an interventional study; about vitamin levels in women |
| Sur Roy SK. Vesicovaginal fistula, with special reference to those complicated with rectovaginal fistula or complete perineal tear. Journal of the Indian Medical Association. 1952;21(12):499-506. | Ineligible study design: single group |
| Takano S, Boutros M, Wexner SD. Gracilis muscle transposition for complex perineal fistulas and sinuses: a systematic literature review of surgical outcomes. Journal of the American College of Surgeons. 2014;219(2):313-23. | Ineligible population: not obstetric fistula |
| Takayanagi A, Masumori N, Saito T, Tsukamoto T. The outcomes of surgical repairs of vesicovaginal fistula in 16 patients. Journal of Obstetrics & Gynaecology. 2014;34(2):169-71. | Ineligible population: not obstetric fistula |
| Tancer ML. A report of thirty-four instances of urethrovaginal and bladder neck fistulas. Surgery, Gynecology & Obstetrics. 1993;177(1):77-80. | Ineligible population: less than 80% of the women had fistula of obstetric aetiology |
| Tarnay C, Kalyebara P, Kayondo M. Recurrence rate of incontinence; and fertility among women success fully repaired for obstetric fistula at a tertiary hospital, Mbarara, South-Western Uganda. Female Pelvic Medicine and Reconstructive Surgery. 2021;27:S62-3. | Ineligible study design: single group |
| Tebeu PM, Maninzou SD, Kengne Fosso G, Jemea B, Fomulu JN, Rochat CH. Risk factors for obstetric vesicovaginal fistula at University Teaching Hospital, Yaounde, Cameroon. International Journal of Gynecology & Obstetrics. 2012;118(3):256-8. | Ineligible study design: assesses risk factors; not an interventional study |
| Tebeu PM, Maninzou SD, Takam D, Nguefack-Tsague G, Fomulu JN, Rochat CH. Surgical outcome following treatment of obstetric vesicovaginal fistula among HIV-positive and HIV-negative patients in Cameroon. International Journal of Gynecology & Obstetrics. 2014;125(2):168-9. | Ineligible intervention: type of surgical procedure not described |
| Thai Clinical Trials Registry (TCTR). The benefit of an anal purse-string suture to decrease contamination during vaginal surgery: a prospective, randomized controlled trial. Trial ID: TCTR20151211001. 2015. [Accessed 16 February 2022]. | Ineligible design: pelvic organ prolapse and urinary incontinence, not obstetric fistula |
| Theofanides MC, Sui W, Sebesta EM, Onyeji I, Matulay JT, Chung DE. Vesicovaginal fistulas in the developed world: An analysis of disease characteristics, treatments, and complications of surgical repair using the ACS-NSQIP database. Neurourology & Urodynamics. 2017;36(6):1622-8. | Ineligible population: fistula not described as obstetric |
| Tobias-Machado M, Mattos PAL, Juliano CAB, da Costa Jr RMM, Juliano RV, Pompeo ACL. Transluminal approaches to vesicorectal fistula repair. International Brazilian Journal of Urology. 2014;40(2):283-4. | Ineligible study design: describes operative technique and case series |
| Tozer PJ, Balmforth D, Kayani B, Rahbour G, Hart AL, Phillips RK. Surgical management of rectovaginal fistula in a tertiary referral centre: many techniques are needed. Colorectal Disease. 2013;15(7):871-7. | Ineligible population: less than 80% of the women had fistula of obstetric aetiology |
| Traore SI, Dembele O, Traore S, Diallo A, Maiga A, Sylla M, et al. [Urogenital fistulas at Sikasso: about 150 cases]. The Pan African Medical Journal. 2019;33:133. | Ineligible study design: cross-sectional |
| Trompetto M, Realis Luc A, Novelli E, Tutino R, Clerico G, Gallo G. Use of the Martius advancement flap for low rectovaginal fistulas. Colorectal Disease. 2019;21(12):1421-8. | Ineligbile study design: single group, pre-post |
| Uddin SW, Biswas M, Sharifa J. Repair of vesicovaginal fistula: Experience of 30 cases and analysis of outcome predictors. International Journal of Urology. 2020;27:160. | Ineligible population: report says that “most” fistula were of obstetric origin but exact number or percentage of overall cases not reported |
| Umoiyoho AJ, Inyang-Etoh EC, Etukumana EA. Obstetric fistula repair: experience with hospital-based outreach approach in Nigeria. Global Journal of Health Science. 2012;4(5):40-5. | Ineligible study design: single group |
| Umoiyoho AJ, Inyang-Etoh EC, Abah GM, Abasiattai AM, Akaiso OE. Quality of life following successful repair of vesicovaginal fistula in Nigeria. Rural & Remote Health. 2011;11(3):1734. | Ineligible study design: cross-sectional |
| Uribe N, Millan M, Minguez M, Ballester C, Asencio F, Sanchiz V, et al. Clinical and manometric results of endorectal advancement flaps for complex anal fistula. International Journal of Colorectal Disease. 2007;22(3):259-64. | Ineligible study design: single arm, no subgrouping or comparison |
| Van Onkelen R, Arends L, Gosselink M, Thijsse S, Schouten R. Transanal advancement flap repair versus ligation of the intersphincteric fistula tract: Systematic review and meta-analysis of fistula healing and postoperative faecal continence. Colorectal Disease. 2014;16(3):18. | Ineligible population: not obstetric fistula |
| Vennesland O, Flateland I. Vesicovaginal fistulas. [Norwegian]. Tidsskrift For Den Norske Laegeforening. 1984;104:870-1. | Ineligible study design: case series |
| Veronikis DK, Nichols DH, Spino C. The Noble-Mengert-Fish operation-revisited: a composite approach for persistent rectovaginal fistulas and complex perineal defects. American Journal of Obstetrics & Gynecology. 1998;179(6):1411-6; discussion 6-7. | Ineligible population: less than 80% of the women had fistula of obstetric aetiology |
| Vimercati A, Dellino M, Crupano FM, Gargano G, Cicinelli E. Ultrasonic assessment of cesarean section scar to vesicovaginal fold distance: an instrument to estimate pre-labor uterine rupture risk. Journal of Maternal-Fetal & Neonatal Medicine. 2021;8:1-5. | Ineligible study design: single group diagnostic study |
| Waaldijk K. The immediate surgical management of fresh obstetric fistulas with catheter and/or early closure. International Journal of Gynaecology & Obstetrics. 1994;45(1):11-6. | Ineligible study design: case series |
| Waaldijk K. Surgical classification of obstetric fistulas. International Journal of Gynecology & Obstetrics. 1995;49(2):161-3. | Ineligible study design: describes classification system for fistula |
| Wahab F, Nasir A, Manan F. Outcome of VVF repair without omental interposition. JPMA - Journal of the Pakistan Medical Association. 2016;66(5):590-2. | Ineligible study design: single group |
| Walker SH, Ambauen-Berger B, Saha SL, Akhter S. Quality of life among women in Bangladesh following ileal conduit urinary diversion operations for irreparable vesicovaginal fistula and bladder exstrophy: observational study. BJOG: An International Journal of Obstetrics & Gynaecology. 2018;125(5):616-22. | Ineligible study design: single group |
| Wang Y, Hadley HR. Nondelayed transvaginal repair of high lying vesicovaginal fistula. Journal of Urology. 1990;144(1):34-6. | Ineligible study design: single group |
| Warner R, Beardmore-Gray A, Pakzad M, Hamid R, Ockrim J, Greenwell T. The cost effectiveness of vaginal versus abdominal repair of vesicovaginal fistulae. International Urogynecology Journal. 2020;31(7):1363-9. | Ineligible population: less than 80% of the women had fistula of obstetric aetiology |
| Wexner SD, Ruiz DE, Genua J, Nogueras JJ, Weiss EG, Zmora O. Gracilis muscle interposition for the treatment of rectourethral, rectovaginal, and pouch-vaginal fistulas: results in 53 patients. Annals of Surgery. 2008;248(1):39-43. | Ineligible population: not obstetric fistula |
| Wiechell H, Massenbach WV. Long term results of operative therapy of vesicovagino-rectal fistulas (author's transl). Geburtshilfe und Frauenheilkunde. 1974;34(1):29-35. | Ineligible study design: case series |
| Wilson AL, Chipeta E, Kalilani-Phiri L, Taulo F, Tsui AO. Fertility and pregnancy outcomes among women with obstetric fistula in rural Malawi. International Journal of Gynaecology & Obstetrics. 2011;113(3):196-8. | Ineligible study design: single arm, no subgrouping or comparison |
| Wright J. Circumferential urethral fistulae in Sub-Saharan Africa, current outcomes and future prospects. International Urogynecology Journal. 2015;26(8):1209-12. | Ineligible intervention: type of surgical procedure not described |
| Wu D, Zhu G, Song W, Yang Z, He D. Comparison of surgical outcomes of laparoscopic versus robotic assisted repair of vesicovaginal fistula: Initial experience from northwest china. International Journal of Urology. 2018;25:312. | Ineligible population: iatrogenic fistula, not obstetric fistula |
| Xiong Y, Tang Y, Huang F, Liu L, Zhang X. Transperitoneal laparoscopic repair of vesicovaginal fistula for patients with supratrigonal fistula: comparison with open transperitoneal technique. International Urogynecology Journal. 2016;27(9):1415-22. | Ineligible population: less than 80% of the women had fistula of obstetric aetiology |
| Yassin N, Askari A, Jenkins JT, Faiz O, Hart A, Phillips R. The use of biological 'infill' materials for the treatment of recto-vaginal fistulas-a systematic review. Colorectal Disease. 2014;3:104. | Ineligible population: fistula not described as obstetric |
| Yismaw L, Alemu K, Addis A, Alene M. Time to recovery from obstetric fistula and determinants in Gondar university teaching and referral hospital, northwest Ethiopia. BMC Women's Health. 2019;19(1):5. | Ineligible intervention: type of surgical procedure not described |
| Young-Gon KIM, Young-Kyung P. Clinical Observation of the Vesicovaginal Fistulas. Korean Journal of Urology. 1983;24(4):649-52. | Ineligible population: less than 80% of the women had fistula of obstetric aetiology |
| Yuan X, Chen H, Chen C, Yang M, Li Q, Gao R, et al. Minimally invasive treatment of mid-low rectovaginal fistula: a transanal endoscopic surgery study. Surgical Endoscopy. 2020;34(9):3971-7. | Ineligible study design: case series |
| Zabin F. Problem of developing residual urinary incontinence after successful repair of obstetric fistula. Journal of Obstetrics and Gynaecology Research. 2017;43:200. | Ineligible population: study focuses on women who have urinary incontinence after obstetric fistula repair and not on obstetric fistula |
| Zabin F, Akhter S, Sultana M. Residual urinary incontinence after successful repair of obstetric fistula. Bangladesh Journal of Obstetrics and Gynecology. 2015;30:86-91. | Ineligible population: study focuses on women who have urinary incontinence after obstetric fistula repair and not on obstetric fistula |
| Zakeri S, Hotouras A, Bhan C, Chan C. Gracilis muscle interposition for the repair of rectovaginal fistula (RVF): A systematic literature review. Colorectal Disease. 2014;2:202. | Ineligible population: less than 80% of the women had fistula of obstetric aetiology |
| Zheng AX, Anderson FW. Obstetric fistula in low-income countries. International Journal of Gynaecology & Obstetrics. 2009;104(2):85-9. | Ineligible study design: systematic review of case series |
| Zhou L, Yang TX, Luo DY, Chen SL, Liao BH, Li H, et al. Factors Influencing Repair Outcomes of Vesicovaginal Fistula: A Retrospective Review of 139 Procedures. Urologia Internationalis. 2017;99(1):22-8. | Ineligible population: less than 80% of the women had fistula of obstetric aetiology |
| Zhou P, Deng W, Li J, Pan H, Wang Y, Song C, et al. Transvesical versus extravesical approach to laparoscopic posthysterectomy vesicovaginal fistula repair: A retrospective study from two medical centers. Neurourology & Urodynamics. 2021;40(6):1593-9. | Ineligible population: post-hysterectomy fistula, not obstetric fistula |
| Zhou Q, Liu ZM, Chen HX, Ren DL, Lin HC. Stapled transperineal repair for low- and mid-level rectovaginal fistulas: A 5-year experience and comparison with sutured repair. World Journal of Gastroenterology. 2021;27(14):1451-64. | Ineligible population: less than 80% of the women had fistula of obstetric aetiology |
| Zimmermann MS, Hoffmann M, Hildebrand P, Bouchard R, Schorcht A, Bader FG, et al. Surgical repair of rectovaginal fistulas--a challenge. International Journal of Colorectal Disease. 2011;26(6):817-9. | Ineligible study design: case report |

| **Excluded from citation chaining** | |
| --- | --- |
| **Study reference** | **Reason for exclusion** |
| Ahmad S, Nishtar A, Hafeez GA, Khan Z. Management of vesico-vaginal fistulas in women. International Journal of Gynaecology & Obstetrics. 2005;88(1):71-5. | Ineligible study design: case series |
| Balde FB, Diallo AB, Toure A, Kante D, Diallo TMO, Lamadine A, et al. Risk Factors for Urinary Incontinence after Obstetric Vesicovaginal Fistula Closure in Guinea. Surgical Science. 2021;12:8. | Ineligible intervention: type of surgical procedure not described |
| Barros P. Use of the Folley catheter in the surgical treatment of vesicovaginal fistulas. Gynecologie Pratique. 1960;11:383-90. | Ineligible study design: not an intervention study; describes operative techniques |
| Browning A. Prevention of residual urinary incontinence following successful repair of obstetric vesico-vaginal fistula using a fibro-muscular sling. BJOG: An International Journal of Obstetrics & Gynaecology. 2004;111(4):357-61. | Ineligible study design: not an intervention study; describes operative techniques |
| Cardenas-Trowers O, Heusinkveld J, Hatch K. Simple and effective: transvaginal vesico-vaginal fistula repair with a modified Latzko technique. International Urogynecology Journal. 2018;29:767-9. | Ineligible study design: not an intervention study; describes operative techniques and includes a case study |
| Das M, Hahm KS, LaRocca AA, Luna CA, Mendez K, Hoffman R, et al. A Low-Cost, Easily Deployable Vesicovaginal Fistula Occluding Device for Providing Interim Continence Journal of Medical Devices. 2022. | Ineligible study design: proof of concept for a device |
| Devakumar H, Chandrasekaran N, Alas A, Martin L, Davila GW, Hurtado E. Transvaginal Repair of Complex Rectovaginal Fistulas Using the Porcine Urinary Bladder Matrix as an Augmenting Graft. Female Pelvic Medicine and Reconstructive Surgery. 2017;23(3):e25-8. | Ineligible study design: two case reports |
| Elkins T. Surgery for the obstetric vesico-vaginal fistula: a review of 100 operations in 82 patients. American Journal of Obstetrics & Gynecology. 1994;170(4):1118-20. | Ineligible study design: compares multiple populations undertaking interventions with other populations undertaking other interventions; not an eligible design |
| Elkins TE, Ghosh TS, Stocker R. Transvaginal mobilization and utilization of the anterior bladder wall to repair vesicovaginal fistulas involving the urethra. Obstetrics & Gynecology. 1992;79(3):455-60. | Ineligible study design: not comparative |
| Gerten KA, Venkatesh S, Norman AM, Shu'aibu J, Richter HE. Pilot study utilizing a patient educational brochure at a vesicovaginal fistula hospital in Nigeria, Africa. International Urogynecology Journal and Pelvic Floor Dysfunction. 2009;20:33–37. | Ineligible study design: single group feasibility study |
| Ghoniem GN, Monga M. Modified Pubovaginal Sling and Martius Graft for Repair of the Recurrent Vesicovaginal Fistula Involving the Internal Urinary Sphincter. European Urology. 1995;27:241-5. | Ineligible study design: case report |
| Gray Sears CL, Schenkman N, Lockrow EG. Use of End-to-End Anastomotic Sizer with Occlusion Balloon to Prevent Loss of Pneumoperitoneum in Robotic Vesicovaginal Fistula Repair. Urology. 2007;70(3):581-2. | Ineligible study design: not an intervention study; describes operative technique |
| Gupta NP, Mishra S, Mishra A, Seth A, Anand A. Outcome of Repeat Supratrigonal Obstetric Vesicovaginal Fistula Repair after Previous Failed Repair. Urologia Internationalis. 2012;88:259-62. | Ineligible study design: single group, not comparative |
| Gutman RE, Dodson JL, Mostwin JL. Complications of treatment of obstetric fistula in the developing world: Gynatresia, urinary incontinence, and urinary diversion. International Journal of Gynaecology & Obstetrics. 2007;99(S1):S57-S64. | Ineligible study design: literature review, not systematic review |
| Hawkins L, Spitzer RF, Christoffersen-Deb A, Leah J, Mabeya H. Characteristics and surgical success of patients presenting for repair of obstetric fistula in western Kenya. International Journal of Gynecology & Obstetrics. 2013;120:178-82. | Ineligible intervention: type of surgical procedure not described |
| Homaira R, Khatun S, Zabin F. A Study on Different Surgical Methods used for Repair of Vesicovaginal Fistulas in Dhaka Medical College Hospital. Medicine Today. 2010;22(1):12-4. | Ineligible study design: cross-sectional |
| Husain A, Johnson K, Glowacki CA, Osias J, Wheeless Jr CR, Asrat K, et al. Surgical Management of Complex Obstetric Fistula in Eritrea. Journal of Women's Health. 2005;14(9):839-44. | Ineligible study design: single group, not comparative |
| Hussen S, Melese E. Time-to-recovery from obstetric fistula and associated factors: The case of Harar Hamlin Fistula Center. Ethiopian Journal of Health Development. 2017;31(2):85-95. | Ineligible intervention: type of surgical procedure not described |
| Johnson KA, Turan JM, Hailemariam L, Mengsteab E, Jena D, Polan ML. The role of counseling for obstetric fistula patients: Lessons learned from Eritrea. Patient Education and Counseling. 2010;80(2):262-5. | Ineligible study design: single group, pre-post |
| Lilungulu A, Gumodoka B, Nassoro M, Soka P, Stephen K. Obstetric fistulae, birth out comes, and surgical repair outcomes: a retrospective analysis of hospital-based data in Dodoma, Tanzania. South Sudan Medical Journal. 2018;11(4):93-6. | Ineligible intervention: surgery only described as either vaginal or abdominal |
| Melamud O, Eichel L, Turbow B, Shanberg A. Laparoscopic vesicovaginal fistula repair with robotic reconstruction. Urology. 2005;65(1):163-6. | Ineligible study design: case report |
| Mohammad RH. A community program for women’s health and development: implications for the long-term care of women with  fistulas. International Journal of Gynecology & Obstetrics. 2007;99(S1):S137-42. | Ineligible study design: not a clinical study; describes the procedures of a specific fistula program |
| Morgan MA, Polan ML, Melecot HH, Debru B, Sleemi A, Husain A. Experience with a low-pressure colonic pouch (Mainz II) urinary diversion for irreparable vesicovaginal fistula and bladder extrophy in East Africa. International Urogynecology Journal. 2009;20:1163-8. | Ineligible study design: case report |
| Munoz O, Bowling CB, Gerten KA, Taryor R, Norman AM, Szychowski JM, et al. Factors influencing post-operative short-term outcomes of vesicovaginal fistula repairs in a community hospital in Liberia. British Journal of Medical and Surgical Urology. 2011;4(6):259-65. | Ineligible study design: single group assignment; compares outcomes, not groups |
| Norman AM, Gerten KA, Ibrahim J, Richter HE. A modified Mainz II pouch technique for management of refractory vesicovaginal fistulas: Patient focused outcomes. International Journal of Gynaecology & Obstetrics. 2008;101(1):35-8. | Ineligible study design: case series |
| Ojengbede OA, Baba Y, Morhason-Bello IO, Armah M, Dimiti A, Buwa D, Kariom M. Group psychological therapy in obstetric fistula care: a complementary recipe for the accompanying mental ill health morbidities? African Journal of Reproductive Health. 2014;18:155–159. | Ineligible study design: single group, pre-post |
| Oluwasolaa TAO, Bello OO. Clinical and Psychosocial Outcomes of Obstetrics Fistulae in Sub-Saharan Africa – A Review of Literature. Journal of Basic and Clinical Reproductive Sciences. 2020;11(9):1. | Ineligible study design: not a systematic review |
| Pope RJ, Brown RH, Chipungu E, Hollier Jr LH, Wilkinson JP. The use of Singapore flaps for vaginal reconstruction in women with vaginal stenosis with obstetric fistula: a surgical technique. BJOG: An International Journal of Obstetrics & Gynaecology. 2017;125(6):751-6. | Ineligible study design: not an intervention study; describes operative technique and a case study |
| Rangnekar NP, Imdad Ali N, Kaul SA, Pathak HR. Role of the Martius procedure in the management of urinary–vaginal fistulas. Journal of the American College of Surgeons. 2000;191(3):259-63. | Ineligbile population: says most of the participants had obstetric trauma as the cause of fistula, but exact figures regarding aetiology of fistula not reported |
| Raz S, Bregg KJ, Nitti VW, Sussman E. Transvaginal Repair of Vesicovaginal Fistula Using a Peritoneal Flap. The Journal of Urology. 1993;150(1):56-9. | Ineligible study design: not an intervention study; describes operative technique |
| Roenneburg ML, Genadry R, Wheeless Jr CR. Repair of obstetric vesicovaginal fistulas in Africa. American Journal of Obstetrics & Gynecology. 2006;195(6):1748-52. | Ineligible intervention: procedure only described as vaginal or abdominal |
| Rothenberger DA, Christenson CE, Balcos EG, Schottler JL, Nemer FD, Vivatvongs S, et al. Endorectal advancement flap for treatment of simple rectovaginal fistula. Diseases of the Colon & Rectum. 1982;25:297-300. | Ineligible study design: describes an operative technique and a case series |
| Santosa KB, Pramana IB, Tirtayasa PM, Yudiana IW, Duarsa GW, Oka AA. The management of vesicovaginal fistulae at Urology Division, Sanglah Hospital, Bali. International Journal of Medical Reviews and Case Reports. 2019;3(7):399-402. | Ineligible population: less than 80% of total participants had obstetric fistula |
| Savan K, Ekin M, Kupelioglu L, Oral S, Yasar L. Surgical repair of genitourinary fistulae: comparison of our experience at Turkey and Niger. Archives of Gynecology & Obstetrics. 2010;282:649-53. | Ineligible population: less than 80% of total participants had obstetric fistula |
| Sayegh AS, La Riva A, Perez LC, Rangel E, Medina LG, Adamic B, et al. Robotic-assisted vesicovaginal fistula repair using a vaginal cuff flap. International Urogynecology Journal. 2022. | Ineligible study design: describes an operative technique and a case study |
| Sharifiaghdas F, Taheri M. The use of a rotational bladder flap for the repair of recurrent mixed trigonal–supratrigonal vesicovaginal fistulas. International Journal of Gynaecology & Obstetrics. 2012;119(1):18-20. | Ineligible study design: describes an operative technique and a case series |
| Shoukry MS, Hassouna ME, El-Salmy S, Abdel-Karim AM. Vaginal flap re-enforcement of vesico-vaginal fistula repair. International Urogynecology Journal. 2010;21:829-33. | Ineligible study design: describes an operative technique and a single group |
| Siddiqui ME, Ali A, Ayub M, Masood F, Akbar H, Razzaq A, et al. Comparison of Transvaginal Repair of Vesicovaginal Fistula with and without Martius Flap. Annals of Punjab Medical College. 2021;15(1):73-6. | Ineligible population: less than 80% of total participants had obstetric fistula |
| Singh O, Gupta SS, Mathur RK. Urogenital Fistulas in Women: 5-year Experience at a Single Center. Urology Journal. 2010;7(1):35-9. | Ineligible population: less than 80% of total participants had obstetric fistula |
| Singh V, Sinha RJ, Mehrotra S, Gupta DK, Gupta S. Transperitoneal Transvesical Laparoscopic Repair of Vesicovaginal Fistulae: Experience of a Tertiary Care Centre in Northern India. Current Urology. 2013;7:75-82. | Ineligible study design: describes an operative technique and a single group |
| Streit–Ciećkiewicz D, Nowakowski L, Grzybowska ME, Futyma K. Predictive value of classification systems and single fistula-related factors in surgical management of vesicovaginal fistula. Neurourology & Urodynamics. 2020;40(1):529-37. | Ineligible population: not described as having obstetric fistula |
| Waaldijk K. Immediate Indwelling Bladder Catheterization at Postpartum Urine Leakage — Personal Experience of 1200 Patients. Tropical Doctor. 1997;27(4):227-8. | Ineligible study design: single group |
| Wall LL. Where should obstetric vesico-vaginal fistulas be repaired: At the district general hospital or a specialized fistula center? International Journal of Gynecology & Obstetrics. 2007;99:S28-S31. | Ineligible publication type: editorial |
| Wein AJ, Malloy TR, Carpiniello VL, Murphy JJ. Repair of vesicovaginal fistula by a suprapubic transvesical approach. Surgery, Gynecology & Obstetrics. 1980;150(1):57-60. | Ineligible population: less than 80% of total participants had obstetric fistula |
| Williams G. The Addis Ababa Fistula Hospital: An holistic approach to the management of patients with vesicovaginal fistulae. The Surgeon. 2007;5(1):54-7. | Ineligible study design: not a clinical study; lecture describing procedures of a fistula program |
| Wilson SM, Sikkema KJ, Watt MH, Masenga GG, Mosha MV. Psychological Symptoms and Social Functioning Following Repair of Obstetric Fistula in a Low-Income Setting. Maternal and Child Health Journal. 2016;20:941-5. | Ineligible study design: single group, pre-post |
| Yuh LM, Rothschild JG. Complications and Long-Term Sequelae of Bladder Fistula Repair. Current Bladder Dysfunction Reports. 2016;11:317-24. | Ineligible study design: literature review, not systematic review |
