# Supplementary material for: Interventions for treating obstetric fistula: An evidence gap map
Source: PLOS Glob Public Health. 2023 Jan 26;3(1):e0001481. doi: 10.1371/journal.pgph.0001481 (PMC10021774; doi:10.1371/journal.pgph.0001481)
Supplement: S6 Table — (DOCX) [file pgph.0001481.s008.docx]

**S6 Table: Details of studies with multiple reports**

| Study ID | Primary study report | Other study reports |
| --- | --- | --- |
| Barone 2015 | Barone MA, Widmer M, Arrowsmith S, Ruminjo J, Seuc A, Landry E, et al. Breakdown of simple female genital fistula repair after 7 day versus 14 day postoperative bladder catheterisation: a randomised, controlled, open-label, non-inferiority trial. Lancet. 2015;386(9988):56‐62. | 1. Barone MA, Frajzyngier V, Arrowsmith S, Ruminjo J, Seuc A, Landry E, et al. Non-inferiority of short-term urethral catheterization following fistula repair surgery: study protocol for a randomized controlled trial. BMC Women's Health. 2012;12:5.  2. Ruminjo J, Barone M, Arrowsmith S, Khisa W, Muleta M, Widmer M. Translating randomized clinical trial findings to practice: non-inferiority of short duration bladder catheterization after fistula repair. International Journal of Gynecology and Obstetrics (Var Pagings). 2015;131:E313.  3. ClinicalTrials.gov. 2011. Non-inferiority of Short-term Catheterization Following Fistula Repair Surgery. Trial ID: NCT01428830. 2011. [Accessed 16 February 2022] |
| Castille 2014 | Castille YJ, Avocetien C, Zaongo D, Colas JM, Peabody JO, Rochat CH. Impact of a program of physiotherapy and health education on the outcome of obstetric fistula surgery. International Journal of Gynaecology & Obstetrics. 2014;124(1):77-80. | Castille YJ, Avocetien C, Zaongo D, Colas JM, Peabody JO, Rochat CH. One-year follow-up of women who participated in a physiotherapy and health education program before and after obstetric fistula surgery. International Journal of Gynaecology & Obstetrics. 2015;128(3):264-6. |
| Nardos 2012 | Nardos R, Menber B, Browning A. Outcome of obstetric fistula repair after 10-day versus 14-day Foley catheterization. International Journal of Gynecology and Obstetrics. 2012;118(1):21‐3. | Nardos R, Member B, Browning A. Obstetric fistula repair outcome comparing 10 day vs. 14 day foley catheterization: a prospective randomized study. Female Pelvic Medicine & Reconstructive Surgery. 2011;17(5):S64. |
| Pope 2021 | Pope R, Browning A, Chipungu E, George JOM, Tamimu M, Wilkinson J. Prophylactic Autologous Slings at the Time of Obstetric Fistula Repair: a Randomized Clinical Trial. Female Pelvic Medicine & Reconstructive Surgery. 2021;27(2):78‐84. | ClinicalTrials.gov. Pubococcygeus Versus Rectus Sheath Sling for Goh Class 3 and 4 Vesico-vaginal Fistulas: a Randomized Controlled Trial. Trial ID: NCT03236922. 2017. [Accessed 16 February 2022]. |
| Torloni 2018 | Torloni MR, Riera R, Rogozinska E, Tuncalp O, Gulmezoglu AM, Widmer M. Systematic review of shorter versus longer duration of bladder catheterization after surgical repair of urinary obstetric fistula. International Journal of Gynecology & Obstetrics. 2018;142(1):15-22. | Widmer M, Torloni R, Tuncalp O. Short versus long duration of bladder catheterization after the surgical repair of urinary obstetric fistula: a systematic review protocol. PROSPERO. 2017. |
| Watt 2017 | Watt MH, Mosha MV, Platt AC, Sikkema KJ, Wilson SM, Turner EL, et al. A nurse-delivered mental health intervention for obstetric fistula patients in Tanzania: results of a pilot randomized controlled trial. Pilot & Feasibility Studies. 2017;3:35. | Watt MH, Wilson SM, Sikkema KJ, Velloza J, Mosha MV, Masenga GG, et al. Development of an intervention to improve mental health for obstetric fistula patients in Tanzania. Evaluation and Program Planning. 2015;50:1-9.  ClinicalTrials.gov. Development of Mental Health Treatment for Obstetric Fistula Patients in Tanzania. Trial ID: NCT01934075. 2013. [Accessed 16 February 2022]. |
