# Supplementary material for: Interventions for treating obstetric fistula: An evidence gap map
Source: PLOS Glob Public Health. 2023 Jan 26;3(1):e0001481. doi: 10.1371/journal.pgph.0001481 (PMC10021774; doi:10.1371/journal.pgph.0001481)
Supplement: S7 Table — (DOCX) [file pgph.0001481.s009.docx]

**S7 Table: Reasons for Studies Awaiting Classification**

| **Study reference** | **Reason for awaiting classification** |
| --- | --- |
| Anoukoum T, Attipou KK, Agoda-Koussema LK, Akpadza K, Ayite EA. Epidemiological, aetiological and treatment aspects of obstetrical fistula in Togo. [French]. Progres en Urologie. 2010;20:71-6. | Full text is in a language other than English and not possible to exclude on abstract. Translation required to determine eligibility. |
| Bansal A, Singh V, Sankhwar SN, Goel A, Sinha RJ, Singh BP, et al. Experience of tertiary care centre in repairing vesico-vaginal fistula with or without interposition flap: a prospective randomized study. Indian Journal of Urology. 2016;32:S113. | Abstract only. Not enough information within the abstract to be able to determine eligibility. |
| Baraket O, Moussa M, Chennoufi B, Bouchouha S. [Les fistules recto-vaginales d'origine obstetricale: aspects epidemiologiques et approaches therapeutiques. Etude multicentrique tunisienne]. Tunisie Medicale. 2014;92(11):686-9. | Full text is in a language other than English and not possible to exclude on abstract. Translation required to determine eligibility. |
| Barone M, Frajzyngier V, Ruminjo J, Asiimwe F, Hamidou Barry T, Bello A, et al. Determinants of fistula repair post-operative outcomes: A prospective cohort study. International Journal of Gynecology and Obstetrics. 2012;3:S167. | Abstract only. Not enough information within the abstract to be able to determine eligibility. |
| Benchekroun A, Lakrissa A, Essakalli HN, Faik M, Abakka T, Hachimi M, et al. Vesicovaginal fistula. Apropos of 600 cases [French]. Journal d'Urologie. 1987;93(3):151-8. | Full text is in a language other than English and not possible to exclude on abstract. Translation required to determine eligibility. |
| Borowka A, Witeska A. [Results of surgical treatment of vesicovaginal fistula by the intraperitoneal-transvesical approach]. Ginekologia Polska. 1981;52(8):739-42. | Full text is in a language other than English and not possible to exclude on abstract. Translation required to determine eligibility. |
| Bouya PA, Nganongo WI, Lomin D, Iloki LH. [Retrospective study of 34 urogenital fistulas of obstetricalal origin]. Gynecologie, Obstetrique & Fertilite. 2002;30(10):780-3. | Full text is in a language other than English and not possible to exclude on abstract. Translation required to determine eligibility. |
| Brandt FT, Albuquerque CDC. Tratamento de fístula vesico-cervicovaginal e vesicovaginal pela técnica do auto-enxerto de mucosa vesical: em modelo experimental e em mulheres. Jornal Brasileiro de Ginecologia. 1998;108(3):59-62. | Full text is in a language other than English and not possible to exclude on abstract. Translation required to determine eligibility. |
| Bup-Wan KIM, Sung-Choon LEE. Clinical Observation on the Management of Vesicovaginal Fistulas. Korean Journal of Urology. 1982;24(1):665-9. | Full text is in a language other than English and not possible to exclude on abstract. Translation required to determine eligibility. |
| Casaretto E, Llayora R, Olivato C, Reche F, Santiago A. Transposición perineal del músculo recto interno: uso en el sinus perineal persistente y en la fístula rectovaginal. Revista Argentina de Cirugía. 1998;75(3):84-6. | Full text is in a language other than English and not possible to exclude on abstract. Translation required to determine eligibility. |
| Castro MAD, de Oliveira NJ, Fernandes FS, Ruiz AB, Franco KC, Silva GRF, et al. Atualizaçäo em fístulas uretrovaginais e vesicovaginais. Brazilian Journal of Medical and Biological Research. 1997;72(1):101-2, 5-6, 8. | Full text is in a language other than English and not possible to exclude on abstract. Translation required to determine eligibility. |
| Cerdan Santacruz C, Cerdan Miguel J. Rectovaginal fistulas: A permanent therapeutic challenge [Spanish]. Progresos de Obstetricia y Ginecologia. 2017;60:150-9. | Full text is in a language other than English and not possible to exclude on abstract. Translation required to determine eligibility. |
| Chen C, Yin L. Retrospective research minimally invasive treatment of mid-low rectovaginal fistula. Diseases of the Colon and Rectum. 2019;62:e325. | Abstract only. Not enough information within the abstract to be able to determine eligibility. |
| Clearwater WL, Rochat CH, Aholou R, Hughes-Hogan L, Foma Jean De Dieu Y, Banks E, et al. Patient factors and clinical outcomes inwomen with obstetric fistula treated in Benin, West Africa. International Urogynecology Journal. 2019;30:S338-S9. | Abstract only. Not enough information within the abstract to be able to determine eligibility. |
| Corbetta JP, Deparci A, González O, Casal JM, Verdinelli J, Ale R, et al. Fístulas urinarias en la práctica urológica. La Prensa Médica Argentina. 2005;92(8):542-7. | Full text is in a language other than English and not possible to exclude on abstract. Translation required to determine eligibility. |
| Crestani A, Dal Moro F. [Surgical treatment of rectourinary fistulas: review of the literature]. Urologia (Treviso). 2015;82(1):30-5. | Full text is in a language other than English and not possible to exclude on abstract. Translation required to determine eligibility. |
| Diallo AB, Sy T, Bah MD, Diallo TM, Barry MS, Bah I, et al. [Obstetrical vesico-vaginal fistula in Guinea: Data analysis of three sites of treatment at Engender Health ONG]. Progres en Urologie. 2016;26(3):145-51. | Full text is in a language other than English and not possible to exclude on abstract. Translation required to determine eligibility. |
| Docquier J. [Urogenital fistulas of obstetrical origin. Apropos of 421 cases]. Acta Urologica Belgica. 1988;56(4):535-44. | Full text is in a language other than English and not possible to exclude on abstract. Translation required to determine eligibility. |
| Drissi M, Karmouni T, Tazi K, El Khader K, Koutani A, Ibn Attya A, et al. Vesicouterine fistulas: An experience of 17 years. [French]. Progres en Urologie. 2008;18(3):173-6. | Full text is in a language other than English and not possible to exclude on abstract. Translation required to determine eligibility. |
| Falandry L. [Treatment of post-partum urogenital fistulas in Africa. 261 cases observed in 10 years]. Progres en Urologie. 1992;2(5):861-73. | Full text is in a language other than English and not possible to exclude on abstract. Translation required to determine eligibility. |
| Falandry L. [Vesicovaginal fistula in Africa. 230 cases]. Presse Medicale. 1992;21(6):241-5. | Full text is in a language other than English and not possible to exclude on abstract. Translation required to determine eligibility. |
| Fischer W. [Long-term analysis of causes, sites and results of treatment of urogenital fistulas at the Charite Gynecologic Clinic]. Zentralblatt fur Gynakologie. 1990;112(12):747-55. | Full text is in a language other than English and not possible to exclude on abstract. Translation required to determine eligibility. |
| Fischer W, Lamm D. [Urogenital fistulas in the Berlin University Gynecology Clinic over a 30-year period (1941 to 1970)]. Zentralblatt fur Gynakologie. 1972;94(47):1603-22. | Full text is in a language other than English and not possible to exclude on abstract. Translation required to determine eligibility. |
| Frank VA. [Magnetic obturation of undeveloped vesicovaginal fistulas]. Urologiia i Nefrologiia. 1986(1):47-9. | Full text is in a language other than English and not possible to exclude on abstract. Translation required to determine eligibility. |
| González-Contreras QH, Castañeda-Argáiz R, Rodríguez-Zentner HA, Tapia-Cid de León H, Mejía-Ovalle RR, Espinosa-de Los Monteros A. Interposición de músculo gracilis para reparar fístulas ano y rectovaginales recurrentes. Cirugia y Cirujanos. 2009;77(4):319-21. | Full text is in a language other than English and not possible to exclude on abstract. Translation required to determine eligibility. |
| Hong L, Bingshu LI, Min HU, Yanxiang C, Xuexian XU. The clinical effects of the transvaginal vesicovaginal fistula repair operation mediated by the Foley catheter. Chinese Journal of General Practitioners. 2011;6:256-8. | Full text is in a language other than English and not possible to exclude on abstract. Translation required to determine eligibility. |
| Hongmei XI, Yingli LIN. Study on the effect of action-oriented health education in the patients with permanent urinary bladder fistula. Chongqing Medicine. 2016(36):5116-7,20. | Full text is in a language other than English and not possible to exclude on abstract. Translation required to determine eligibility. |
| Hull TL, Sapci I, Lightner AL. Gracilis Flap Repair for Reoperative Rectovaginal Fistula. Diseases of the Colon & Rectum. 2021;25:25. | Full text is in a language other than English and not possible to exclude on abstract. Translation required to determine eligibility. |
| In-Geun SEO. Early Repair of Rectovaginal Fistula with Simple Primary Closure of the Anal Opening. Journal of the Korean Medical Association. 1997:900-5. | Full text is in a language other than English and not possible to exclude on abstract. Translation required to determine eligibility. |
| Jianhui WU, Shiqiang Y, Yong XU, Hongshun MA. A retrospective clinical study of urogenital fistula caused by gynecological and obstetrical surgery. Chinese Journal of Urology. 2014;35(9):686-90. | Full text is in a language other than English and not possible to exclude on abstract. Translation required to determine eligibility. |
| Jinhua LI, Wenchun LI, Ge C. Clinical comparative study of two kinds of extra-dissection and loose seton operation in the treatment of complex anal fistula complicated with abscess. Chinese Journal of Primary Medicine and Pharmacy. 2018;25(18):2394-6. | Full text is in a language other than English and not possible to exclude on abstract. Translation required to determine eligibility. |
| Kaeser CT, Stachowicz A, Karram M. 13: Surgical management of recurrent rectovaginal fistula. American Journal of Obstetrics and Gynecology. 2019;220:S771. | Abstract only. Not enough information within the abstract to be able to determine eligibility. |
| Kpatcha TM, Wangala P, Botcho G, Tchandana M, Nembuzu D, Aboubakari AS. [Epidemiologic, anatomoclinic and therapeutic profil of urogenital and rectovaginal fistula in TOGO]. Progres en Urologie. 2020;30(11):597-603. | Full text is in a language other than English and not possible to exclude on abstract. Translation required to determine eligibility. |
| Krzeski T, Witeska A, Borowka A. [Results of surgical treatment of vesicovaginal fistula]. Polski Tygodnik Lekarski. 1981;36(7):257-8. | Full text is in a language other than English and not possible to exclude on abstract. Translation required to determine eligibility. |
| Massoudnia N. [Surgical treatment of vesico-vaginal fistulas]. Munchener Medizinische Wochenschrift. 1970;112(31):1429-31. | Full text is in a language other than English and not possible to exclude on abstract. Translation required to determine eligibility. |
| Meziane A, Joual A, Fadaili A, Seddiki S, Bennani S, El Mrini M. [Vaginal flap urethroplasty in the treatment of urethral destruction of obstetric origin]. African Journal of Urology. 2004;10(2):101-3. | Full text is in a language other than English and not possible to exclude on abstract. Translation required to determine eligibility. |
| Montellatto NI, Monti PR, Menezes de Goes G. A posicao genitopeitoral no tratamento da fistula vesicovaginal. Jornal Brasileiro de Urologia. 1984;10(2):79-80. | Full text is in a language other than English and not possible to exclude on abstract. Translation required to determine eligibility. |
| Montoya-Martinez G, Ledesma-Rodriguez AG, Serrano-Brambila E, Moreno-Palacios J. Vesicovaginal fistula: Open approach versus laparoscopic abdominal. Ginecologia y Obstetricia de Mexico. 2013;81:587-92. | Full text is in a language other than English and not possible to exclude on abstract. Translation required to determine eligibility. |
| Oussama B, Makrem M, Badis C, Samy B. Recto-vaginal obstetrical fistulas: Therapeutic attitudes. Multicenter tunisian study. [French]. Tunisie Medicale. 2015;92(11):686-9. | Full text is in a language other than English and not possible to exclude on abstract. Translation required to determine eligibility. |
| Reisenauer C. [Surgical Management of Obstetric Rectovaginal Fistulas: the Gynaecological Point of View]. Zentralblatt fur Chirurgie. 2019;144(4):380-6. | Full text is in a language other than English and not possible to exclude on abstract. Translation required to determine eligibility. |
| Rodríguez Carballo M, Sánchez Falcón A, Prado Rodríguez M. Fístula vesico vaginal: modificación de técnica quirúrgica. Revista Médica Electrónica. 2009;31(6). | Full text is in a language other than English and not possible to exclude on abstract. Translation required to determine eligibility. |
| Sang-Sung LEE, Chun-Il KIM, Sung-Choon LEE. Vesicovaginal fistula: 20years of experience. Korean Journal of Urology. 1991:970-5. | Full text is in a language other than English and not possible to exclude on abstract. Translation required to determine eligibility. |
| Schmiedt E, Carl P. [Transvesical closure of urethrovaginal fistula using plastic surgery]. Urologe (Ausg A). 1972;11(6):309-13. | Full text is in a language other than English and not possible to exclude on abstract. Translation required to determine eligibility. |
| Sitkovskii NB. [The treatment of atresia of the anus and rectum with fistula into the genital system in girls]. Khirurgiia. 1966;42(7):80-4. | Full text is in a language other than English and not possible to exclude on abstract. Translation required to determine eligibility. |
| Tebeu PM, Fokom-Domgue J, Kengne Fosso G, Tjek Biyaga P, Nelson Fomulu J, Rochat CH. Comparative study of the outcome of surgical management of vesico-vaginal fistulas with and without interposition of the Martius graft: A Cameroonian experience [French]. Progres en urologie : journal de l'Association francaise d'urologie et de la Societe francaise d'urologie. 2015;25:1225-31. | Full text is in a language other than English and not possible to exclude on abstract. Translation required to determine eligibility. |
| Tian XK. [A retrospective study of the operative failure for treatment of urinary fistula (author's transl)]. Chung-Hua Fu Chan Ko Tsa Chih [Chinese Journal of Obstetrics & Gynecology]. 1979;14(3):182-4. | Full text is in a language other than English and not possible to exclude on abstract. Translation required to determine eligibility. |
| Weidi C, Goumei Q, Yangmin W, Xiaoling LI, Wenting ZHU, Chen Z. Effects of information motivation behavior model on self-management behavior and self perceived burden of elderly patients with permanent bladder fistula. Modern Clinical Nursing. 2017;16(5):8-13. | Full text is in a language other than English and not possible to exclude on abstract. Translation required to determine eligibility. |
| Yang Y, Chen YK, Che XY, Wu SL. [Prognostic factors for failure of transvaginal repair of vesicovaginal fistula: A nested case-control study]. Beijing da Xue Xue Bao Yi Xue Ban/Journal of Peking University Health Sciences. 2021;53(4):675-9. | Full text is in a language other than English and not possible to exclude on abstract. Translation required to determine eligibility. |
| Yang Y, Chen YK, Che XY, Wu SL. [Prognostic factors for failure of transvaginal repair of vesicovaginal fistula: A nested case-control study]. Beijing da Xue Xue Bao Yi Xue Ban/Journal of Peking University Health Sciences. 2021;53(4):675-9. | Full text is in a language other than English and not possible to exclude on abstract. Translation required to determine eligibility. |
| Yanqin YOU, Xiaoyu FU, Ke H, Lei S. Application of acellular allograft dermal tissue patch in the repair of transvaginal rectovaginal fistula. Chinese Journal of Obstetrics and Gynecology. 2012;47(12):920-2. | Full text is in a language other than English and not possible to exclude on abstract. Translation required to determine eligibility. |
| You YQ, Fu XY, Huang K, Song L. [Application of acellular allograft dermal tissue patch in the repair of transvaginal rectovaginal fistula]. [Chinese]. Zhonghua Fu Chan Ke Za Zhi. 2012;47:920-2. | Full text is in a language other than English and not possible to exclude on abstract. Translation required to determine eligibility. |
| Yu Wei Z, Hao HU, Peng Xiao Z, Ran Yi SUN, Rui Huan W, Xin Ke XU. Comparison and discussion of different surgical methods used to treat vesicovaginal fistulas. Journal of Peking University (Health Sciences). 2017(6):889-92. | Full text is in a language other than English and not possible to exclude on abstract. Translation required to determine eligibility. |
| Yuke C, Wei YU, Yang Y, Jihong D, Yunxiang X, Shiliang WU. The influences of the fistula's location on the procedure and outcome of a transvaginal vesicovaginal repair. Chinese Journal of Urology. 2016;37(12):892-5. | Full text is in a language other than English and not possible to exclude on abstract. Translation required to determine eligibility. |
| Yuke C, Yang Y, Wei YU, Jihong D, Yunxiang X, Shiliang WU. The surgical treatment for complex vesicovaginal fistula. Chinese Journal of Urology. 2017;38(10):737-40. | Full text is in a language other than English and not possible to exclude on abstract. Translation required to determine eligibility. |
| Zacher W, Knorr B. [Variations in the etiology and therapy of gynecologic-urologic fistulas treated in our clinic]. Zeitschrift fur Urologie und Nephrologie. 1976;69(11):825-9. | Full text is in a language other than English and not possible to exclude on abstract. Translation required to determine eligibility. |
| Zanini P, Angeli A. [Early treatment of vesicovaginal fistulas caused by obstetric injury]. Minerva Ginecologica. 1976;28(12):1021-32. | Full text is in a language other than English and not possible to exclude on abstract. Translation required to determine eligibility. |
| Zhanfei SHE, Yi LYU. Research progress of the treatment of rectovaginal fistulas. Chinese Journal of Gastrointestinal Surgery. 2014;17(12):1250-4. | Full text is in a language other than English and not possible to exclude on abstract. Translation required to determine eligibility. |
| Zhang WY, Hu H, Zhang XP, Sun YR, Wang HR, Xu KX. Comparison and discussion of different surgical methods used to treat vesicovaginal fistulas [Chinese]. Beijing Da Xue Xue Bao. 2017;49(5):889-92. | Full text is in a language other than English and not possible to exclude on abstract. Translation required to determine eligibility. |
