# Supplementary material for: Interventions for treating obstetric fistula: An evidence gap map
Source: PLOS Glob Public Health. 2023 Jan 26;3(1):e0001481. doi: 10.1371/journal.pgph.0001481 (PMC10021774; doi:10.1371/journal.pgph.0001481)
Supplement: S8 Table — (DOCX) [file pgph.0001481.s010.docx]

**S8 Table: Characteristics of ongoing studies**

| **Study ID** | **Design** | **Inclusion criteria** | **Exclusion criteria** | **Intervention** | **Comparator** | **Outcomes** |
| --- | --- | --- | --- | --- | --- | --- |
| Ctri 2021/ 09/036394 | RCT | Women with surpraregional vesicovaginal fistulae; not previously undergone repair | Not consenting to trial; history of irradiation or genito-urinary tuberculosis; requiring ancillary procedure that can affect operating time and blood loss; complex multiple fistulae | Laparoscopic Miklos Moore extravesical technique | Laparoscopic Mini O’Conor intravesical technique | Primary: operative time  Secondary: amount of anticholinergics required; early postoperative pain score; need for analgesics; laparoscopic suturing time taken; success rate of procedure |
| NCT 03029130 | RCT | Women with vesicovaginal fistula; at time of initial catheter removal (7 or 14 days post-repair) have demonstrable fistulous leak on dye test | HIV; concomitant bladder stones; one or more ureters outside the bladder; urethrovaginal fistula; multiple fistulae; dye leak/ fistulous leak present at end of surgery; radiation-induced fistula; fistula caused by cancer or infection; continence procedures being performed; rectovaginal fistula; pregnancy; fistula breakdown of > 2 cm identified on postoperative dye test | Foley catheter extension for 14 days | Immediate discharge; no catheter extension | Primary: fistula closure (subjective and objective)  Secondary: urinary incontinence; closure and continence at hospital discharge; complications |
| NCT 04267757 | RCT | Rectovaginal fistula caused by obstetric issues; female; > 20 years old | Other causes of rectovaginal fistula (e.g. Crohn’s disease, malignant fistula); complex and recurrent fistula | Rectovaginal fistula repair with Martius flap | Rectovaginal fistula repair without Martius flap | Primary: recurrence in rectovaginal fistula  Secondary: NR |
| NCT 04676568 | RCT | All patients with vesicovaginal fistula | Ureterovaginal fistula | Extravesical surgical repair | Transvesical surgical repair | Primary: operative time; complications (e.g. bladder spasms, bleeding); recurrence of symptoms  Secondary: NR |
| NR: not reported; RCT: randomised controlled trial | | | | | | |
