# Supplementary material for: Interventions for treating obstetric fistula: An evidence gap map
Source: PLOS Glob Public Health. 2023 Jan 26;3(1):e0001481. doi: 10.1371/journal.pgph.0001481 (PMC10021774; doi:10.1371/journal.pgph.0001481)
Supplement: S9 Table — (DOCX) [file pgph.0001481.s011.docx]

**S9 Table: Heat maps of risk of bias assessments across included studies**

| **Summary of risk of bias assessments for RCTs** | | | | | | | |
| --- | --- | --- | --- | --- | --- | --- | --- |
| **Study ID** | Random sequence generation | Allocation concealment | Blinding of participants / personnel | Blinding of outcome assessors | Attrition bias | Selective reporting | Other bias |
| Barone 2015 |  |  |  |  |  |  |  |
| Watt 2017 |  |  |  |  |  |  |  |
| Pope 2021 |  |  |  |  |  |  |  |
| Nardos 2012 |  |  |  |  |  |  |  |
| Shaker 2011 |  |  |  |  |  |  |  |
| Umoiyoho 2017 |  |  |  |  |  |  |  |
| Safan 2009 |  |  |  |  |  |  |  |
| Assessed using the Cochrane ‘Risk of bias’ tool. Key: green = low risk; yellow = unclear risk; red = high risk. Studies ordered by most low-risk domains to least low-risk domains. | | | | | | | |

| **Summary of risk of bias assessments for cohort studies** | | | | | | | | | | | | |
| --- | --- | --- | --- | --- | --- | --- | --- | --- | --- | --- | --- | --- |
| **Study ID** | Were groups similar and from similar populations? | Were exposures similarly measured for assignment? | Were exposures measured in a valid and reliable way? | Were confounders identified? | Were strategies to deal with confounders identified? | Were groups/ participants initially free of the outcome? | Were outcomes measured in a valid and reliable way? | Was the length of follow-up reported and sufficient? | Was follow-up complete and dropout reasons described? | Were strategies to deal with loss to follow-up complete? | Were appropriate statistical analyses used? |  |
| Nardos 2009 |  |  |  |  |  |  |  |  |  |  |  |  |
| Raassen 2008 |  |  |  |  |  |  |  |  |  |  |  |  |
| Kirschner 2010 |  |  |  |  |  |  |  |  |  |  |  |  |
| Ojewola 2018 |  |  |  |  |  |  |  |  |  |  |  |  |
| Hategekimana 2006 |  |  |  |  |  |  |  |  |  |  |  |  |
| Castille 2014 |  |  |  |  |  |  |  |  |  |  |  |  |
| Waaldijk 2004 |  |  |  |  |  |  |  |  |  |  |  |  |
| Browning 2006 |  |  |  |  |  |  |  |  |  |  |  |  |
| Shaikh 2011 |  |  |  |  |  |  |  |  |  |  |  |  |
| Mubiyaki 2017 |  |  |  |  |  |  |  |  |  |  |  |  |
| Pope 2018 |  |  |  |  |  |  |  |  |  |  |  |  |
| Tayler-Smith 2013 |  |  |  |  |  |  |  |  |  |  |  |  |
| Kirschner 2016 |  |  |  |  |  |  |  |  |  |  |  |  |
| Jalbani 2006 |  |  |  |  |  |  |  |  |  |  |  |  |
| Mazier 1995 |  |  |  |  |  |  |  |  |  |  |  |  |
| Falih 2021 |  |  |  |  |  |  |  |  |  |  |  |  |
| Nardos 2008 |  |  |  |  |  |  |  |  |  |  |  |  |
| Browning 2008 |  |  |  |  |  |  |  |  |  |  |  |  |
| Kubba 1991 |  |  |  |  |  |  |  |  |  |  |  |  |
| Assessed using the JBI Checklist for Cohort Studies. Key: green = yes; yellow = unclear risk; red = no; white = not applicable. Studies ordered by most low-risk domains to least low-risk domains. | | | | | | | | | | | | |

| **Summary of risk of bias assessments for case-control studies** | | | | | | | | | | |
| --- | --- | --- | --- | --- | --- | --- | --- | --- | --- | --- |
| **Study ID** | Were groups comparable? | Were cases / controls matched appropriately? | Were cases / controls identified with same criteria? | Were exposures measured in a valid and reliable way? | Was exposure measured the same for cases/ control? | Were confounding factors identified? | Were strategies to deal with confounders identified? | Were outcomes measured in a valid and reliable way? | Was the length of follow-up long enough? | Were appropriate statistical analyses used? |
| Browning 2006 |  |  |  |  |  |  |  |  |  |  |
| Assessed using the JBI Checklist for Case Control Studies. Key: green = yes; yellow = unclear risk; red = no; white = not applicable | | | | | | | | | | |

| **Summary of risk of bias assessments for systematic reviews** | |
| --- | --- |
| **AMSTAR-2 Item** | **Study ID** |
|  | Torloni 2018 |
| Did the research questions and inclusion criteria for the review include components of PICO? |  |
| Critical domain: Did the report of the review contain an explicit statement that the review methods were established prior to the conduct of the review and did the report justify any deviations from protocol? |  |
| Did the review authors explain their selection of the study designs for inclusion in the review? |  |
| Critical domain: Did the review authors use a comprehensive literature search strategy? |  |
| Did the review authors perform study selection in duplicate? |  |
| Did the review authors perform data extraction in duplicate? |  |
| Critical domain: Did the review authors provide a list of excluded studies and justify the exclusions? |  |
| Did the review authors describe the included studies in adequate detail? |  |
| Critical domain: Did the review authors use a satisfactory technique for assessing risk of bias (RoB) in individual studies that were included in the review? |  |
| Did the review authors report on the sources of funding for the studies included in the review? |  |
| Critical domain: If meta-analysis was performed, did the review authors use appropriate methods for statistical combination of results? |  |
| If meta-analysis was performed, did the review authors assess the potential impact of RoB in individual studies on the results on the meta-analysis or other evidence synthesis? |  |
| Critical domain: Did the review authors account for RoB in primary studies when interpreting/discussing the results of the review? |  |
| Did the review authors provide a satisfactory explanation for, and discussion of, any heterogeneity observed in the results of the review? |  |
| Critical domain: If they performed quantitative synthesis did the review authors carry out an adequate investigation of publication bias (small study bias) and discuss its likely impact on the review? |  |
| Did the review authors report any potential sources of conflict of interest, including any funding received for conducting the review? |  |
| Overall confidence | Moderate |
| Assessed using AMSTAR-2. Key: green = yes; yellow = unclear; red = no. Studies ordered by most low-risk domains to least low-risk domains. | |
