# Supplementary material for: Interventions for treating obstetric fistula: An evidence gap map
Source: PLOS Glob Public Health. 2023 Jan 26;3(1):e0001481. doi: 10.1371/journal.pgph.0001481 (PMC10021774; doi:10.1371/journal.pgph.0001481)
Supplement: Attachment — Submitted filename: Response to Reviewers.docx [file pgph.0001481.s012.docx]

**PGPH-D-22-01398**
**Interventions for treating obstetric fistula: an evidence gap map**
**PLOS Global Public Health
Editor and peer review comments and responses from authors
23 November 2022**

**Editor comments**

**1.** Please review your reference list to ensure that it is complete and correct. If you have cited papers that have been retracted, please include the rationale for doing so in the manuscript text, or remove these references and replace them with relevant current references. Any changes to the reference list should be mentioned in the rebuttal letter that accompanies your revised manuscript. If you need to cite a retracted article, indicate the article’s retracted status in the References list and also include a citation and full reference for the retraction notice.

**Response**: We have reviewed the reference list and have not found any papers that have been retracted or revised after publication.

**2**. Please send a completed 'Competing Interests' statement, including any COIs declared by your co-authors. If you have no competing interests to declare, please state "The authors have declared that no competing interests exist". Otherwise please declare all competing interests beginning with the statement "I have read the journal's policy and the authors of this manuscript have the following competing interests:"

**Response**: We have changed the competing interests statement at the bottom of the manuscript to “The authors have declared that no competing interests exist” as we have no conflicts of interest to declare.

**3**. Please amend your detailed Financial Disclosure statement. This is published with the article. It must therefore be completed in full sentences and contain the exact wording you wish to be published.

a. State what role the funders took in the study. If the funders had no role in your study, please state: “The funders had no role in study design, data collection and analysis, decision to publish, or preparation of the manuscript.”
b. If any authors received a salary from any of your funders, please state which authors and which funders.

**Response**: We have added the following statement to the end of the manuscript, in the Financial Disclosure section: “The funders had no role in study design, data collection and analysis, decision to publish, or preparation of the manuscript.”

**4**. Please provide separate figure files in .tif or .eps format only and remove any figures embedded in your manuscript file. Please also ensure that all files are under our size limit of 10MB.

**Response**: We have re-saved all figures in the manuscript as .tif files and re-uploaded these to Editorial Manager as requested.

**5**. We noticed that you used "unpublished data" in the manuscript. We do not allow these references, as the PLOS data access policy requires that all data be either published with the manuscript or made available in a publicly accessible database. Please amend the supplementary material to include the referenced data or remove the references.

**Response**: We are unclear what is meant by this comment. We have not stated that we have used unpublished data within the manuscript. The only reference to unpublished data comes in the ‘Limitations’ section of the Discussion, where we state: “However, grey literature searching was not undertaken for this review, potentially resulting in omissions of small or unpublished studies”. We therefore do not believe any action is required here.

**6**. We have noticed that you have uploaded Supporting Information files, but you have not included a list of legends. Please add a full list of legends for your Supporting Information files after the references list.

**Response**: Thank you for this comment. We have now added a list of the supplementary information contained within the supporting information file at the end of the manuscript, below the references.

**Peer reviewer 1**

**1**. Excellent choice of topic and a well-executed review. Despite the scope of the review being quite broad, the authors rationalized all every step, which contained the information from being too generalized.

**Response**: Many thanks for this kind comment on our work.

**2**. The authors have mentioned the PICO framework. The population of interest was from LMICs. However, survey respondents and studies from HICs are also included. Please clarify.

**Response**: Thank you for this comment. We do note that there were many individuals from HICs who responded to the survey; the survey was targeted at patients or other stakeholders with an interest in fistula and it is possible that some of them were based in HICs but may have for example, worked extensively in LMICs. However, we do not know for whom this was the case as the survey was anonymous apart from the collection of some specific demographic information. As such, we have added the following to the strengths and limitations of the review in the Discussion: “a limitation of the survey is the small number of responses, and the high proportion of respondents who were from high-income countries, despite our attempts to distribute it widely. This may limit the survey’s applicability to different key stakeholders.”

The reviewer is correct in identifying that studies from HICs are included. They are, however, not correct in assuming that our population of interest was restricted to studies from LMICs.  As defined in our PICO framework, stated in S3 table and manuscript paragraph ‘Eligibility criteria’: “Eligible participants were women of any age, living in any setting, with fistula described as being of obstetric aetiology and reported in studies and systematic reviews where at least 80% of the population had fistula of obstetric aetiology.” Hence, although small in number, inclusion of studies undertaken in HICs is appropriate according to our PICO. We feel our current text clarifies this point.

**3**. 'there is currently little to guide patients and practitioners regarding what may be the most effective treatment options for women with obstetric fistula' Surgical correction is scientifically proven to be the most effective treatment option. Even according to your own results (Survey 24/25 and surgical intervention 17/20) However, what is yet to established is a more holistic management approach. Such as adjunct therapies of surgery combined with psychological rehabilitation. So, I would suggest rephrasing this part for clarity.

**Response**: Respectfully, we disagree with the reviewer regarding whether the effectiveness of surgery is proven in the context of this work. The evidence map is not designed to demonstrate effectiveness like a traditional systematic review, but rather to highlight where gaps in evidence may exist, as well as facilitate the potential production of primary and secondary research. We therefore believe that rephrasing our conclusions in this regard does not reflect the purpose of the methodology; the current phrasing highlights the overall lack of evidence in the area.

We appreciate the comment regarding more holistic approaches to treatment and management; one included study (Tayler-Smith et al 2013) did describe catheterisation, physical therapy and surgery (the method of the latter not being described) but most studies assessed surgery alone. We have therefore added the following line into the Implications for research: “Most studies within the evidence map (71%) only reported on surgical interventions; further research into physical therapies, psychological therapies and more “holistic” approaches to treatment and management of obstetric fistula should also be conducted.”

**4**. 'Although there is some evidence surrounding different surgical interventions, there is currently little surrounding psychological interventions, catheter insertion, lifestyle interventions and physical therapies.' I would suggest removing catheter insertion as it is a proven technique for treating early diagnosed, simple fistulas.

**Response**: Respectfully, we wish to rebut this comment. There were only four studies identified for the evidence map that considered bladder catheterisation; one of these was a recent systematic review (Torloni et al 2018) that only included two RCTs. Additionally, we ran our searches in February 2022 to identify this evidence. Given this, we feel that there is currently not enough high-certainty, methodologically rigorous evidence to suggest that catheter insertion is effective in treating obstetric fistulas.

**5**. In implications or further research, I would suggest mentioning management and prevention of obstructed labor. As that is the most prevalent cause in LMICs and is avertible with better labor management.

**Response**: We appreciate the comment from the reviewer and recognise that research into preventing obstructed labour would also be of importance. However, as the purpose of this particular work was to assess the breadth of the evidence on treating and managing obstetric fistula, we feel that commenting on the need for research into preventing obstructed labour, which we have not searched for or assessed in any way, is less relevant to this current work.

**Peer reviewer 2**

**1.** In the manuscript "Interventions for treating obstetric fistula:an evidence gap map", Johnson et. al. address an important public health problem of obstetric fistula that impacts more than 50,000 women annually. The authors first survey stakeholders to guide the framework of creating an evidence map,followed by analysis of several quantitative primary studies and review to produce the first evidence map assessing interventions (surgical and non-invasive interventions) for treating obstetric fistula. The study highlighted a paucity of robust evidence to inform patients, practitioners and policy-makers about interventions for treating obstetric fistula, particularly with regard to non-surgical interventions.

Overall, this study is extremely relevant and valuable to the field of obstetric fistula research and it paves the way for future research in this area. The study is well designed and I believe the manuscript is suitable for publication if the authors address a few minor concerns:

**Response**: Many thanks for this kind comment on our work.

**2**. My primary concern is the small sample size of patients (n=4) in the initial survey. While clinicians and academicians may provide a medical/statistical picture of the condition, the patients are the primary stakeholders here and it would be extremely important to have more patient responders. As the survey provides the foundation to structure outcomes for the evidence map, it becomes important to include more patients or perhaps weigh their responses higher than the rest. The authors have mentioned this factor in their limitations, but it is necessary to highlight this in the results section as well.

**Response**: Many thanks for this comment; we do recognise that the small number of patients who responded to the survey is one of the key limitations of the work. As noted, we have already mentioned this in the Discussion but we also added a short line in the Results to highlight the limited responses from patients as well.

**3**. Similarly, the survey does not include spouses/partners and immediate family members of the patients. They would undoubtedly provide more insight as the condition has a significant impact on the patient’s social and community life, and family support does play an important role in guiding successful public health interventions.

**Response**: Thank you for this comment. In the ‘Strengths and limitations’ we have added the following: “Additionally, only four respondents were patients and no respondents identified as immediate family members of patients”.

**4**. The manuscript suffers from long length, and some sections should be condensed. Particularly, it would be important to emphasize the main findings regarding the lack of non-surgical interventions in current literature and practices - It would be useful to emphasize this in the abstract as well as in individual sections.

**Response**: The abstract is currently at the maximum of 300 words that is allowed by PLOS Global Public Health; however, we have already stated “Most studies (71%) assessed surgical interventions alone.” We feel this draws attention to the fact there is currently little evidence surrounding non-surgical interventions.

With regards to the length of the article overall, we previously attempted to remove anything that seemed superfluous or condensed wording as we are aware this is a long article. However, because of the methodology involved (both a survey and an evidence gap map), and the need to adhere to the Campbell Collaboration reporting standards for evidence gap maps (reported in the supplementary material), we believe that the length is necessary in order to transparently explain the methods used, the results and how the survey feeds into the evidence map.

**5**. The authors mention the need for further robust research is also needed to help address the evidence gaps identified by their study. It would be useful to include the quantitative measures that future studies can include - for example, what would be the best way to evaluate improvements in mental health, and how perhaps improvement in quality of life can be assessed.

**Response**: We have mentioned that there is a need for a core outcome set in the topic area within the Discussion: “a core outcome set would also assist in standardising reporting across trials; these should be published and accessible through a platform such as the Core Outcome Measures in Effectiveness Trials (COMET) website”. The development of a core outcome set in the area would help to establish the best measures for outcomes of interest to key stakeholders including, as mentioned, improvements in mental health.

**6**. The figure legends are either missing (mostly titles are present) or are too short. Please add them and expand on each. Additionally, figure resolutions are not clear after conversion to pdf, the authors should take note of this.

**Response**: We are sorry that these legends did not show; we previously included the legends for Figures 4, 5a and 5b with a key explaining some of the features. We have also expanded on these to note that studies may appear more than once in the evidence map if that study reports on more than one intervention or outcome measure of interest. Additionally, we have added a further legend for Figure 2, to explain the colour coding.

**7**. Figures 4, 5a and 5b are not very intuitive to understand. While the key for the colors are mentioned in the legend, I would suggest the authors include the color key on top of the figures itself so that readers can easily interpret them.

**Response**: We appreciate this comment and have added the legend in the corner of these figures to reiterate the information.

**8**. Minor comment: On Line 122: “such as charities” is repeated twice

**Response**: Thank you for spotting this error; we have now removed the second instance of this phrase.
